# Synthesis, bioactivity assessment, molecular docking and ADMET studies of new chromone congeners exhibiting potent anticancer activity

**DOI:** 10.1038/s41598-024-59606-2

**Published:** 2024-04-26

**Authors:** Heba M. Abo-Salem, Sahar S. M. El Souda, Heba I. Shafey, Khairy M. A. Zoheir, Khadiga M. Ahmed, Kh. Mahmoud, Karima F. Mahrous, Nagwa M. Fawzy

**Affiliations:** 1 Chemistry of Natural Compounds Department, National Research Centre, Dokki, Giza, 12622 Egypt; 2 Cell Biology Department, National Research Center, Dokki, Giza, 12622 Egypt; 3 Pharmacognosy Department, National Research Centre, Dokki, Giza, 12622 Egypt; 4 Chemistry of Natural and Microbial Products Department, National Research Center, Dokki, Giza, 12622 Egypt

**Keywords:** Medicinal chemistry, Organic chemistry

## Abstract

In consideration of the chromones' therapeutic potential and anticancer activity, a new series of chromanone derivatives have been synthesized through a straightforward reaction between 6-formyl-7-hydroxy-5-methoxy-2-methylchromone (**2**) and various organic active compounds. The cytotoxic activity of the newly synthesized congeners was investigated against MCF-7 (human breast cancer), HCT-116 (colon cancer), HepG2 (liver cancer), and normal skin fibroblast cells (BJ1). The obtained data indicated that compounds **14b**, **17**, and **19** induce cytotoxic activity in the breast MCF7, while compounds **6a**, **6b**, **11** and **14c** showed highly potent activity in the colon cancer cell lines. Overall, the results demonstrate that the potential cytotoxic effects of the studied compounds may be based on their ability to induce DNA fragmentation in cancer cell lines, down-regulate the expression level of CDK4 as well as the anti-apoptotic gene Bcl-2 and up-regulate the expression of the pro-apoptotic genes P53 and Bax. Furthermore, compounds **14b** and **14c** showed a dual mechanism of action by inducing apoptosis and cell cycle arrest. The docking studies showed that the binding affinity of the most active cytotoxic compounds within the active pocket of the CDK4 enzyme is stronger due to hydrophobic and H-bonding interactions. These results were found to be consistent with the experimental results.

## Introduction

Currently, cancer is referred to as a “lifestyle disease,” sprinkling from developed to developing countries and showing no discernible differences regarding caste, color, age, or religion. The World Health Organization estimates that cancer is considered the leading cause of mortality worldwide, accounting for roughly 10 million deaths in 2020, or approximately one in every six^1^. Cancer is a multifactorial disease, that results from the mutation of various genes that regulate cell activity in response to specific environmental factors. It is distinguished by uncontrolled cell proliferation that produces expansive masses of aberrant cells that invade and disrupt adjacent normal tissues^2–4^. As first-line cancer treatments, many chemotherapeutics, radiotherapeutics, and immunomodulation agents are available.

In addition to being extremely costly, these drugs also have numerous serious side effects, such as alopecia, nausea, vomiting, and occasionally bone marrow depression, which can result in morbidity^5,6^. For cancer therapy, therefore, new approaches to diagnosis, treatment, and prevention are desperately needed.

Chromones, also known as 1-benzopyran-4-ones, are occur naturally in a wide range of plants, and present in varying amounts in a typical human diet^7,8^. Natural and synthetic chromones showed remarkable biological activities, among them the ability to suppress HIV^9^ and have anticancer^7,8^, anti-microbial^10^, anti-inflammatory^11^, antimalarial^12^, antioxidant, neuroprotective^13^, anti-ulcer effects and so on^8^.

Regarding anti-cancer activity, chromones exhibit activity toward various kinds of tumor cells, and the antiproliferative mechanisms include cytotoxicity, immunomodulation, anti-angiogenesis, chemoprevention, and anti-metastasis^14^.

**Figure 1:**
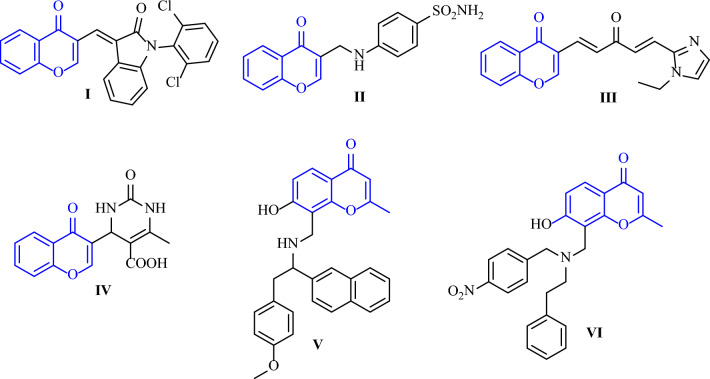
Some biologically active chromone derivatives.

Figure 1 displays the structures of some biologically active chromones. Compound **I** demonstrated potent cytotoxic activity against various cancer cell lines, including leukaemia, colon, prostate, and melanoma^15^. Chromone derivative **II**, in turn, induced apoptosis in both lung and breast cancer cell lines and showed selectivity for isoforms IX and XII of human carbonic anhydrase (hCA)^16^. Chromone attached to 1-alkyl-1H-imidazole-2-yl (**III**) demonstrated excellent activity against prostate cancer cell lines, and 4H-chromone-1,2,3,4-tetrahydropyrimdine-5-carboxylates (**IV**) displayed significant activity against leukaemia cell lines without showing toxicity to normal cell lines^8^. Additionally, Nam et al. developed chromone derivatives **V** and **VI**, which are analogues of lavendustin and both showed prominent activities against A-549 and HCT-15 cell lines^17^.

Because of their therapeutic behaviours and low toxicity, chromones are considered to be an attractive source for the development of new pharmaceuticals. In our ongoing efforts to develop novel chemopreventive treatments for cancer disorders and according to the fact that chromone moiety plays a significant role in the pharmacophores of numerous biologically active compounds with a variety of therapeutic uses^18–22^. The current study seeks to synthesize new chromone derivatives and evaluate their anticancer efficacy starting from the naturally occurring visnagin.

## Result and discussion

Visnagin (**1**) was recognized as a “hit” (active) from the natural products repository screening in the search for new leads for curative cancer treatment. Additionally, it acts as a precursor in the synthesis of many bioactive compounds. Visnagin (**1**) was converted to 6-formyl-7-hydroxy-5-methoxy-2-methylchromone (**2**) by oxidation using 10% potassium dichromate^23^, which underwent further reactions with various organic reagents. The presence of both aldehydic and hydroxyl groups in 2 makes chemical modifications easier and more versatile.

**Figure 2:**
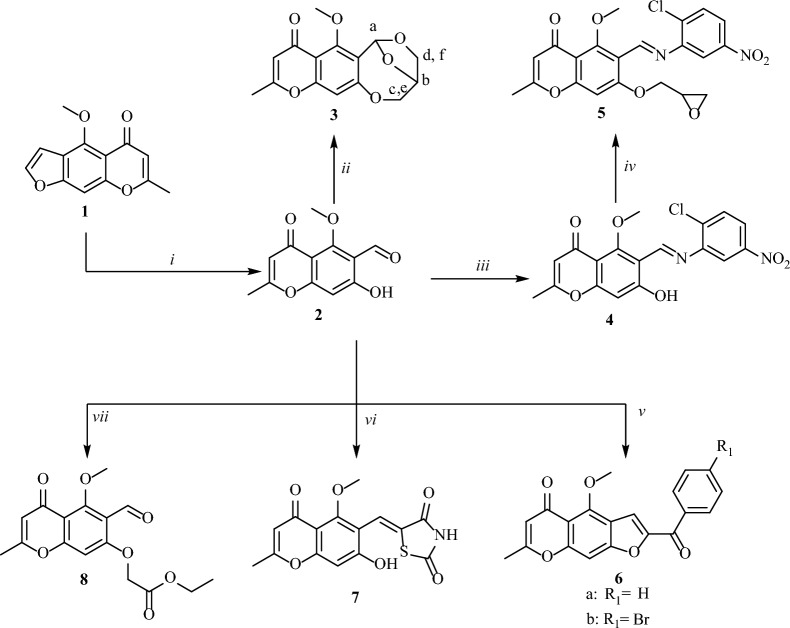
Synthesis of chromone derivatives **3**–**8**; reagent and conditions (i) sulfuric acid (10%), potassium dichromate (10%); (ii) epichlorohydrine, TEA, reflux 3 h.; (iii) 2-chloro-5-nitroaniline, EtOH, gl. AcOH, 2 h.; (iv) epichlorohydrin, acetone, K_2_CO_3_ (v) phenacyl bromides, acetone, K_2_CO_3_; (vi) thiazolidine-2,4-dione, EtOH, piperidine; (vii) bromo ethyl acetate, acetone, K_2_CO_3_.

**Figure 3:**
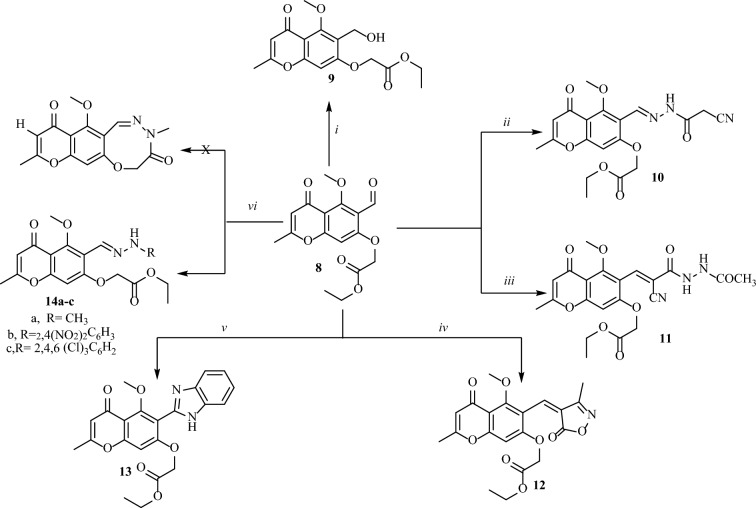
Synthesis of chromone derivatives **9**–**14**: reagent and conditions (i) NaBH_4_, MeOH, dil HCl; (ii) 2-cyanoacetic acid hydrazide, EtOH, gl. AcOH; (iii) N-acetyl-2-cyanoacetohydrazide, EtOH, TEA; (iv) ethyl acetoacetate, NH_2_OH.HCl, anh.CH_3_COONa, EtOH; (v) o-phenylenediamine, p-TsOH, DMF; (vi) hydrazine derivatives, EtOH, gl. AcOH.

**Figure 4:**
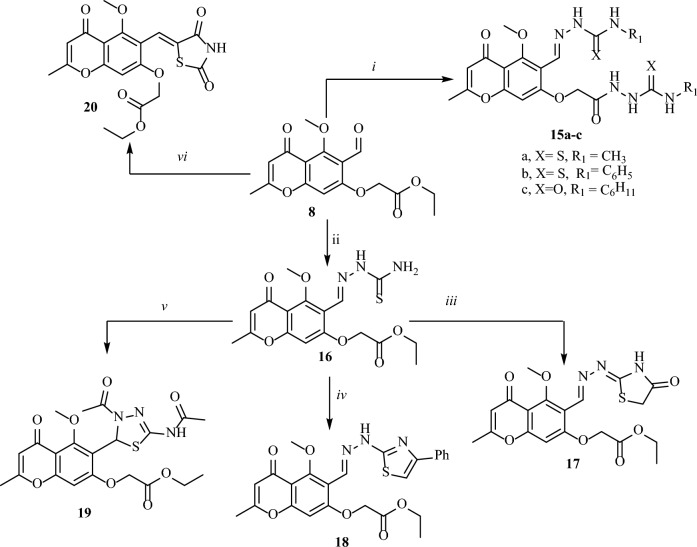
Synthesis of chromone derivatives **15**–**20**: reagent and conditions (i) NH_2_NH_2_.H_2_O, PhNCS; (ii) thiosemicarbazide, EtOH, gl.AcOH; (iii) bromo ethyl acetate, aceone, K_2_CO_3_; (iv) phencyl broide, acetone, K_2_CO_3_; (v) acetic anhydride; (vi) thiazolidine-2,4-dione, ethanol, piperidine.

In the context of our objective to synthesize bioactive chromone derivatives, Figs. 2, 3, and 4 summarized a set of efficient reactions starting from 6-formyl-7-hydroxy-5-methoxy-2-methylchromone (**2**). First, the behaviour of epichlorohydrin toward **2** and its Schiff's base have been studied (Fig. 2). When **2** reacted with epichlorohydrin (1-chloro-2,3-epoxypropane; ECH) in the presence of triethyl amine afforded the corresponding 7-methoxy-10-methyl-3,4-dihydro-2H-3,6-epoxy[1,5]dioxocino[3,2-g]chromen-8(6H)-one (**3**) through interaction of both carbonyl and hydroxyl groups with two mole of ECH (Fig. 2). By blocking the carbonyl group of 2 via its reaction with primary aromatic amine, the obtained Schiff's base **4** then, reacted with ECH afforded the corresponding oxirane derivative **5** (Fig. 2).

Cyclization reaction of **2** with phenacyl bromide derivatives gave the corresponding furo[3,2-g]chromen-5-ones (**6**). Additionally, condensation of **2** with thiazolidine-2,4-dione afforded (Z)-5-((7-hydroxy-5-methoxy-2-methyl-4-oxo-4*H*-chromen-6-yl)methylene) thiazolidine-2,4-dione **(7)** (Fig. 2).

A new starting compound ethyl 2-((6-formyl-5-methoxy-2-methyl-4-oxo-4*H*-chromen-7-yl)oxy)acetate (**8**) had been furnished through the reaction of **2** with ethyl bromoacetate and was used as a building block for many chroman-4-one derivatives. Compound **8** was prepared according to the reported method^24^ and was obtained in high yield, in an extremely pure state and used without further purification (Fig. 2).

Reduction of **8** using sodium borohydride led to the formation of ethyl 2-((6-(hydroxymethyl)-5-methoxy-2-methyl-4-oxo-4*H*-chromen-7-yl)oxy)acetate (**9**).

Acid and/or base-catalyzed condensation reaction of **8** with 2-cyanoacetic acid hydrazide or *N*- acetyl-2-cyanoacetohydrazide in absolute ethanol afforded the corresponding cyanohydrazone derivatives **10** and **11**, respectively (Fig. 3).

Furthermore, one-pot three-component reaction of **8** with ethyl acetoacetate and hydroxylamine hydrochloride afforded (Z)-ethyl 2-((5-methoxy-2-methyl-6-((3-methyl-5-oxoisoxazol-4(5*H*)-ylidene)methyl)-4-oxo-4*H*-chromen-7-yl)oxy)acetate (**12**). While the reaction of **8** with *o*-phenylenediamine using *p*-toluenesulfonic acid as a catalyst led to the formation of ethyl 2-((6-(1*H*-benzo[d]imidazol-2-yl)-5-methoxy-2-methyl-4-oxo-4*H*-chromen-7-yl)oxy)acetate (**13**).

On the other hand, compound **8** was reacted with one mole of methyl hydrazine and/or phenylhydrazine derivatives to obtain the corresponding oxadiazocine derivatives (**X**) but the HNMR data (experimental part) indicated that the hydrazone derivatives **14** were obtained (Fig. 3).

Additionally, a one-pot three-component reaction of **8** with hydrazine hydrate and methyl isothiocyanate, phenyl isothiocyanate or cyclohexyl isocyanate yielded the corresponding carbothioamide and carboxamide derivatives, respectively (**15**) (Fig. 4).

Furthermore, the reaction of **8** with thiosemicarbazide gave the corresponding thiosemicarbazone **16** which under cyclization with ethyl bromoacetate, phenacyl bromide or acetic anhydride afforded the corresponding thiazole and thiadiazole derivatives **17–19**. Finally, ethyl 2-((6-formyl-5-methoxy-2-methyl-4-oxo-4H-chromen-7-yl)oxy)acetate (**8**) was reacted with thiazolidine-2,4-dione yielded the corresponding thiazolidine **20** (Fig. 4).

The chemical structures of the newly synthesized compounds were confirmed based on their IR, ^1^H, ^13^C NMR and mass spectroscopy (Experimental part, Figs. s1–s67).

### Evaluation of in vitro cytotoxic activity

The in vitro *MTT* assay was utilized to assess the cytotoxic activity of the newly synthesized chromone congeners **3–20** on various cancer cell lines including human breast cancer (MCF-7), human colon cancer (HCT-116), human liver cancer (HepG2) as well as human normal Skin fibroblast cells (BJ1). After 48 h of exposure to a single dose concentration of 100 μM, the percentage of cell death was computed in relation to the cells that were left untreated.

According to the obtained results, most of the studied chromone congeners demonstrated selective cytotoxicity against the cancer cell lines MCF-7 and HCT-116, with compounds **3**, **6a**, **7**, **9**, **13**, **14b, 14c**, **15a**, **17**, **18, 19** and **20** showing potent cytotoxic activity against MCF-7 cells ranging from 100 to 65.2%, as compared to the reference positive control drug doxorubicin (DOX) (Table 1).

**Table 1:** Anti-proliferative activity of the newly synthesized compounds against human carcinoma cell lines and normal skin fibroblast cells (BJ-1) at 100 µg/mL.

Compd. no.	Growth inhibition (%)				Compd. no.	Growth inhibition (%)			
	MCF-7	HCT-116	HepG-2	BJ-1		MCF-7	HCT-116	HepG-2	BJ-1
**3**	93.5	100	37.5	95.8	**14a**	24.5	94.2	39.5	2.3
**4**	58.6	85.3	54.2	18.3	**14b**	83.2	75.6	60.6	15.7
**5**	71.6	81.3	54.2	51.6	**14c**	100	85.6	93.5	25.6
**6a**	79.5	96.3	100	7.8	**15a**	70.7	3.5	11.5	6.8
**6b**	36.2	97.5	76.3	2.3	**15b**	43.5	74.3	14.6	2.8
**7**	76.1	100	95.2	93.5	**15c**	35.6	58.3	1.5	63.2
**9**	65.2	12.9	13.5	3.8	**16**	3.4	11.3	37.8	3.6
**10**	3.2	28.5	52.9	5.6	**17**	69.5	31.2	47.2	17.6
**11**	2.8	100	39.1	17.2	**18**	92.6	77.5	70.3	19.8
**12**	16.8	2.5	40.2	3.8	**19**	81.6	36.5	32.8	11.5
**13**	89.6	93.5	74.6	65.3	**20**	100	100	85.6	90.6
**Doxorubicin**	100	100	100	26		100	100	100	26

Regarding, HCT-116 cell lines all compounds showed strong cytotoxic activity ranging from 100 to 75.6%, in comparison to doxorubicin (DOX), except for compounds **9**, **10**, **12**, **15a**, **16**, **17**, and **19**, which exhibited low cytotoxicity ranging from 58.3 to 2.5%, as presented in Table 1**.**

Meanwhile, for HepG2 cancer cell lines, only compounds **6a-b, 7, 13, 14b, 14c, 18** and **20** exhibited potent cytotoxic activity ranging from 100 to 60.6% as compared to doxorubicin (Table 1).

For the most potent compounds that displayed minimal toxicity towards BJ-1 cells, the concentration needed for 50% inhibition of cell viability (IC50) has been computed (Table 2). The data obtained revealed that, in comparison to the reference drug doxorubicin, which had an IC50 of 26.1 µg/mL, compounds **14b**, **17** and **19** induced moderate cytotoxicity against the MCF-7 cancer cell line, with IC50s of 43.1 and 54.8 µg/mL (Table 2).

**Table 2:** IC_*50*_ of the highly anti-proliferative active compounds against human Colon and Human breast cancer cell lines.

Compd.	IC50 (µg/mL)	
	MCF7	HCT116
**4**	–	67.2
**6a**	75.5	35.1
**6b**	–	18.6
**11**	–	29.2
**14b**	43.1	57.7
**14c**	–	53.3
**15a**	73.9	63.7
**15b**	71.5	–
**15c**	64.4	–
**17**	54.8	–
**18**	70.6	58.2
**19**	54.8	–
**Doxorubicin**	26.1	37.6

Interestingly, compounds **6a**, **6b** and **11** demonstrated more effectiveness against HCT-116 cancer cell line than doxorubicin (IC50 = 37.6 µg/mL). They exhibited a descending order of activity (6b < 11 < 6a), with IC50 values of 18.6, 29.2, and 35.1 µg/mL, in that order. While, compounds **14b**, **14c** and **18** displayed moderate cytotoxicity with IC50 values of 57.7, 53.3 and 58.2 µg/mL, respectively (Table 2). Based on these findings, further studies were conducted to investigate the cellular mechanisms underlying the potent cytotoxic effect of certain chromone congeners against MCF-7 and HCT-116.

### Evaluation of DNA fragmentation and gene expression

Based on the preliminary cytotoxic screening, the selectivity of compounds **14b**, **17**, and **19** in the breast cancer cell line (MCF7) and compounds **6a**, **6b**, **11** and **14c** in the colon cancer cell line (HCT116) were identified. Therefore, these compounds were investigated further to determine their mode of action. The main mechanism that controls carcinogenesis is the equilibration of proliferation to programmed cell death. Several vital mechanisms were involved, and different assays related to these two main processes were studied.

### DNA fragmentation

The DNA fragmentation was examined in breast cancer cell lines (MCF-7) and colon cancer cell lines **(**HCT-116) using diphenylamine reaction procedure as well as by DNA gel electrophoresis laddering assay as described in the experimental section.

**Figure 5:**
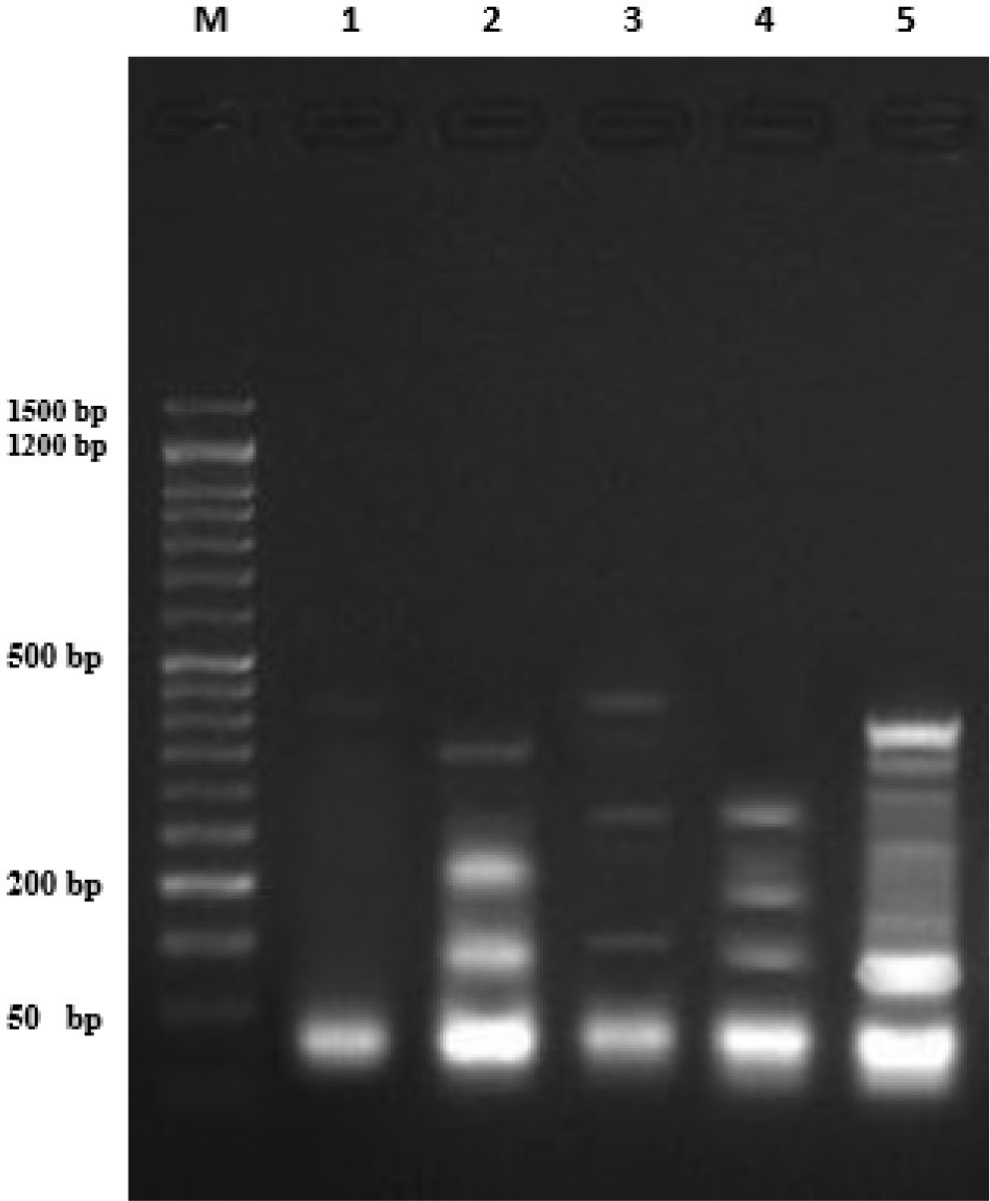
DNA fragmentation detected with Agarose gel in breast cancer cell line (MCF-7) treated with different treatments. M: represent DNA marker, Lane 1: represents MCF-7 negative control (− ve), Lane 2: represents Compound **14b**, Lane 3: represents Compound **17**, Lane 4: represents Compound **19**, Lane 5: represents MCF-7 positive control (Dox) group.

In the breast cancer cell lines (MCF-7), treatments with three different compounds **14b**, **17**, and **19** at IC50 concentration were investigated. The results demonstrated that the negative control cells had significantly lower (*P* < 0.01) DNA fragmentation rates than the treated groups. Compound **14b** had the highest fragmentation rate, while compound **17** had the lowest as displayed in Table 3 and Fig. 5.

**Table 3:** DNA fragmentation detected in breast cancer cell line (MCF-7) samples in different treatments.

Treatment	DNA fragmentation % (M ± SEM)	Change	Inhibition %
Cancer cell lines (− ve)	12.01 ± 0.73^**d**^	0.00	0.00
14b	37.09 ± 0.70^**a**^	25.0	13.64
17	21.88 ± 0.84^**c**^	9.8	− 55.46
19	30.48 ± 0.86^**b**^	18.4	− 16.36
Cancer cell lines (+ ve)	34.09 ± 0.69^**ab**^	22.0	0.00

Means with different superscripts (^a, b, c^) between groups in the same column are significantly different at *P* < 0.05; Cancer cell lines (− ve): negative control (cancer cells without any treatment); Cancer cell lines (+ ve): positive control (cancer cells treated with doxorubicin).

**Figure 6:**
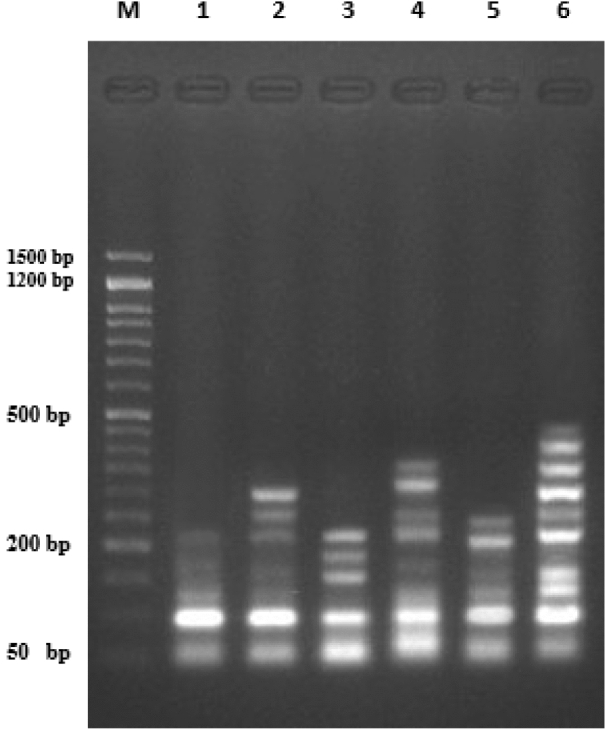
DNA fragmentation detected with Agarose gel in colon cancer cell line (HCT116) treated with different treatments. M: represent DNA marker, Lane 1: represents HcT116 negative control (− ve), Lane 2: represents compound **6a**, Lane 3: represents Compound **6b**, Lane 4: represents Compound **14c**, Lane 5: represents compound **11**, Lane 6 represents HCT116 positive control (Dox) group.

On the other hand, in the colon cancer cell lines (HCT-116) four different treatments with compounds **6a**, **6b**, **11** and **14c** were examined. The negative control had significantly lower (*P* < 0.01) DNA fragmentation rates compared to the treated groups. The positive control treated with Doxo showed the highest fragmentation rate and compounds **6a** and **14c** had higher fragmentation rates than the other tested compounds (Table 4, Fig. 6).

**Table 4:** DNA fragmentation detected in colon cancer cell line (HCT116) samples in different treatments.

Treatment	DNA fragmentation % (M ± SEM)	Change	Inhibition %
Cancer cell lines (− ve)	14.20 ± 0.67^**d**^	0.00	0.00
6a	30.56 ± 0.92^**b**^	16.40	− 25.11
6b	21.49 ± 0.74^**c**^	7.30	− 66.67
11	24.76 ± 0.92^**c**^	10.60	− 51.60
14c	33.93 ± 0.52^**ab**^	19.70	− 10.05
Cancer cell lines (+ ve)	36.10 ± 0.73^**a**^	21.90	0

Means with different superscripts (^a, b, c^) between groups in the same column are significantly different at *P* < 0.05; Cancer cell lines (− ve): negative control (cancer cells without any treatment); Cancer cell lines (+ ve): positive control (cancer cells treated with doxorubicin).

### Gene expression

The families of pro- and anti-apoptotic play a key factor in beating cancer growth their gene expression was assayed.

### Effects of chromone congeners on mRNA expression of P53, BAX, BCL2 and CDK4 on breast cancer (MCF7)

**Figure 7:**
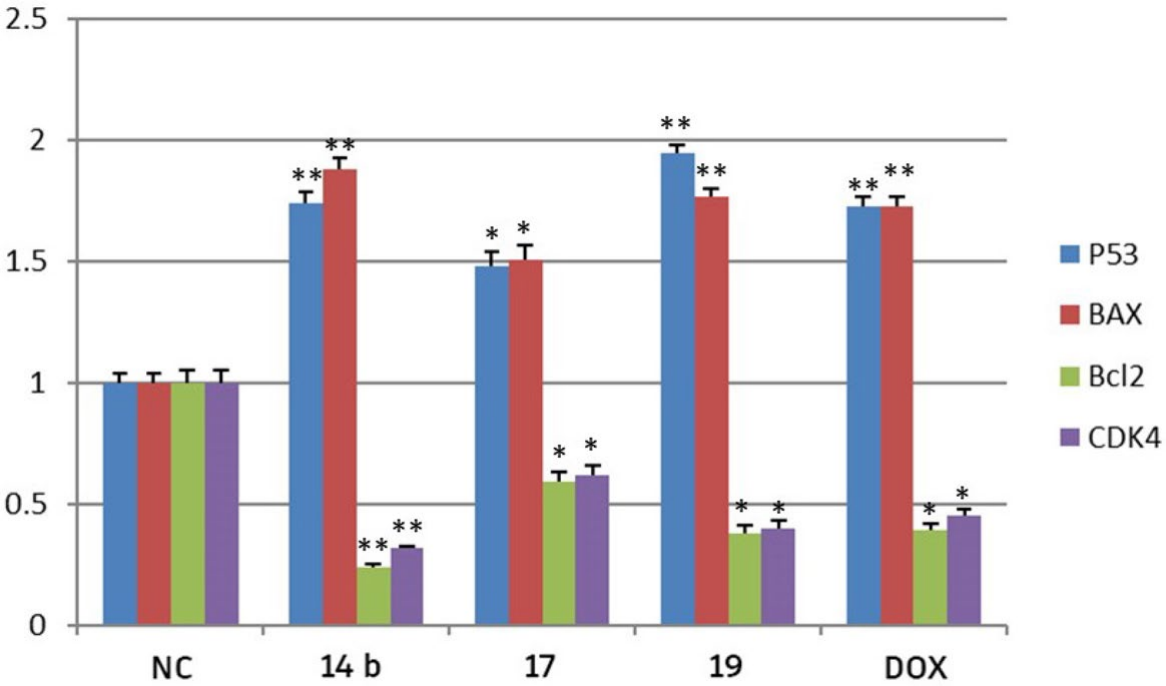
The RT-qPCR validation of mRNA expression for P53, BAX, BCL2, CDK4, in MCF7, Breast cancer cell line (mcF7) among groups of control, DOX; NC: normal control (cancer cells without any treatment); Error bars represents standard error of mean (SEM). Means comparisons were performed by using One-Way ANOVA test. *Significant differences.

The result indicated that the mRNA levels of P53 and Bax were up-regulated, while Bcl-2 and CDK4 were down-regulated in breast cancer (MCF7) cell lines treated with compounds **14b**, **17**, **19** and DOX as compared to the untreated cells. Moreover, the effect of compounds **14b** and **19** was significantly more potent than DOX (Fig. 7).

### Effects of chromone congeners on mRNA expression of P53, BAX, BCL2 and CDK4 on colon cancer (HCT116)

**Figure 8:**
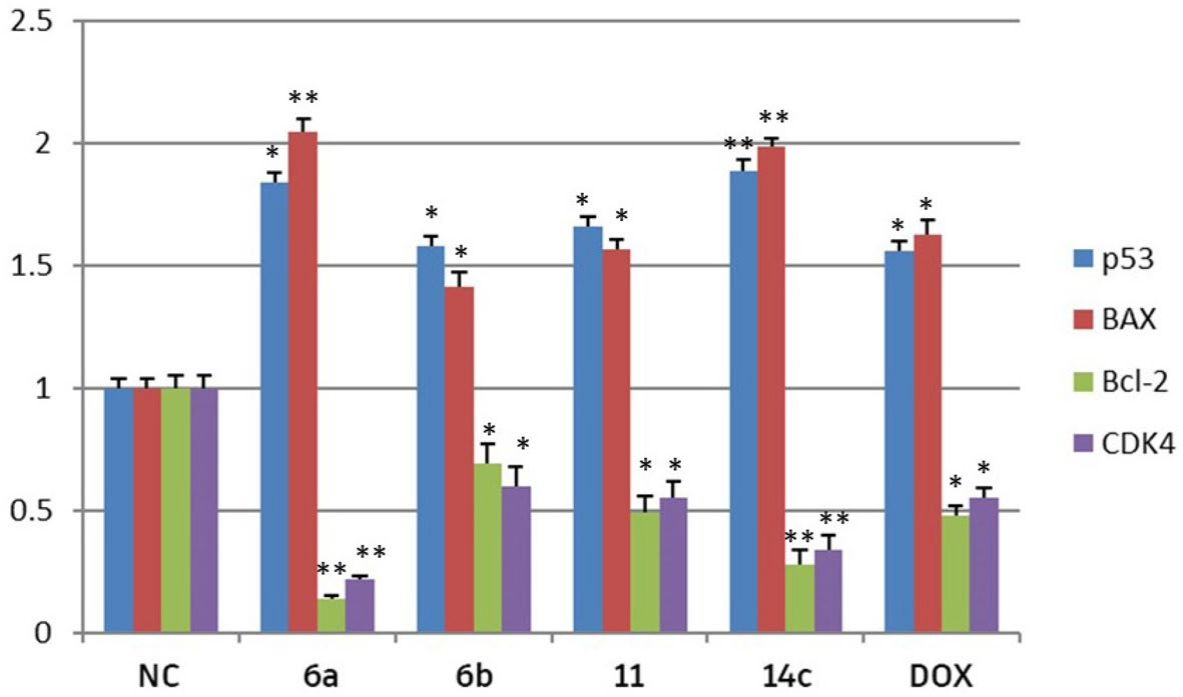
The RT-qPCR validation of mRNA expression for P53, BAX, BCL2, CDK4, in MCF7, Colon cancer cell line (HcT116) among groups of control, DOX; NC: normal control (cancer cells without any treatment); Error bars represents standard error of mean (SEM). Means comparisons were performed by using One-Way ANOVA test. *Significant differences.

The result revealed that treating HCT116 with compounds **6a**, **6b**, **11**, **14c**, and DOX resulted in increased mRNA levels of both P53 and Bax. However, these treatments also led to lower levels of CDK4 and Bcl-2 mRNA when compared to the negative control. Compounds **6a** and **14c** had significantly higher mRNA expression in P53 and Bax than DOX. Moreover, they also led to significant CDK4 and Bcl-2 down-regulation than DOX (Fig. 8). On the other hand, the down-regulation of **11** was found to be similar to that of DOX as displayed in Fig. 8.

Recently, the inhibition of CDKs (cyclin-dependent kinases) that regulate the cell cycle, such as CDK4/6, is a crucial goal for cancer researchers to prevent inappropriate cell division and promote inhibitory barriers. In the transition of cells into the S phase, CDK4 and CDK6 play a critical role as mediators and are essential for the initiation, and survival of several cancer types^25^.

Our results indicate that all of the tested compounds were able to down-regulate CDK4 mRNA levels in treated cancer cells. It was observed that compounds **6a**, **14b**, **14c** and **19** demonstrated a higher efficacy in comparison to DOX, whereas compounds **17** and **6b** showed a lower efficacy than DOX. On the other hand, tumors generally show elevated Bcl-2 expression^26,27^.

The pro-apoptotic proteins in the Bcl-2 family, such as Bax and Bak, play an important role in promoting the release of cytochrome c and ROS, which are important signals in the apoptosis cascade. These pro-apoptotic proteins are activated by BH3-only proteins and inhibited by the function of Bcl-2 and its relative Bcl-Xl^28^. Therefore, down-regulating Bcl-2 and up-regulating Bax can be a hopeful approach to control tumorigenesis. Our studied compounds were found to suppress Bcl-2 and activate Bax, which could contribute to the apoptosis mechanism. Furthermore, the up-regulation of P53 can also be vital for the activation of apoptosis^29^.

It is known that P53 suppresses tumors by inhibiting cell proliferation through the activation of P21 protein, as well as by initiating apoptosis. The mechanism of P53's action is both transcriptionally dependent and independent^30^. Also, P53 plays a significant role in various cell signaling mechanisms, such as cell-cycle arrest, DNA repair, differentiation, and cell death (apoptosis). Thus, the ability of the tested compounds to up-regulate P53 can help activate apoptosis. It is worth mentioning that cancers in patients with changes or clampdown of P53 function are not responsive to conservative chemotherapeutic drugs, but rather respond to new genotoxic chemotherapeutics that act via the P53 pathway.

### Flow cytometer analysis

Annexin V and propidium iodide staining are commonly used method for identifying apoptotic cellular death. In the presence of Ca^2+^ ions, annexin V has a strong binding affinity for phosphatidylserine a membrane phospholipid that is translocated from the inner to the outer side of the cell membrane during apoptosis. However, propidium iodide has the ability to bind DNA can only enter into necrotic or late apoptotic cells.

The effectiveness of various compounds on cancer cell lines can be ascertained using this technique for detecting apoptosis.

### Effects of compounds on MCF cells

The apoptotic rate of compounds **14b**, **17**, **19** and DOX in the Breast cancer cell line (MCF7) was determined using the Annexin V–FITC/PI Double Staining Kit.

**Figure 9:**
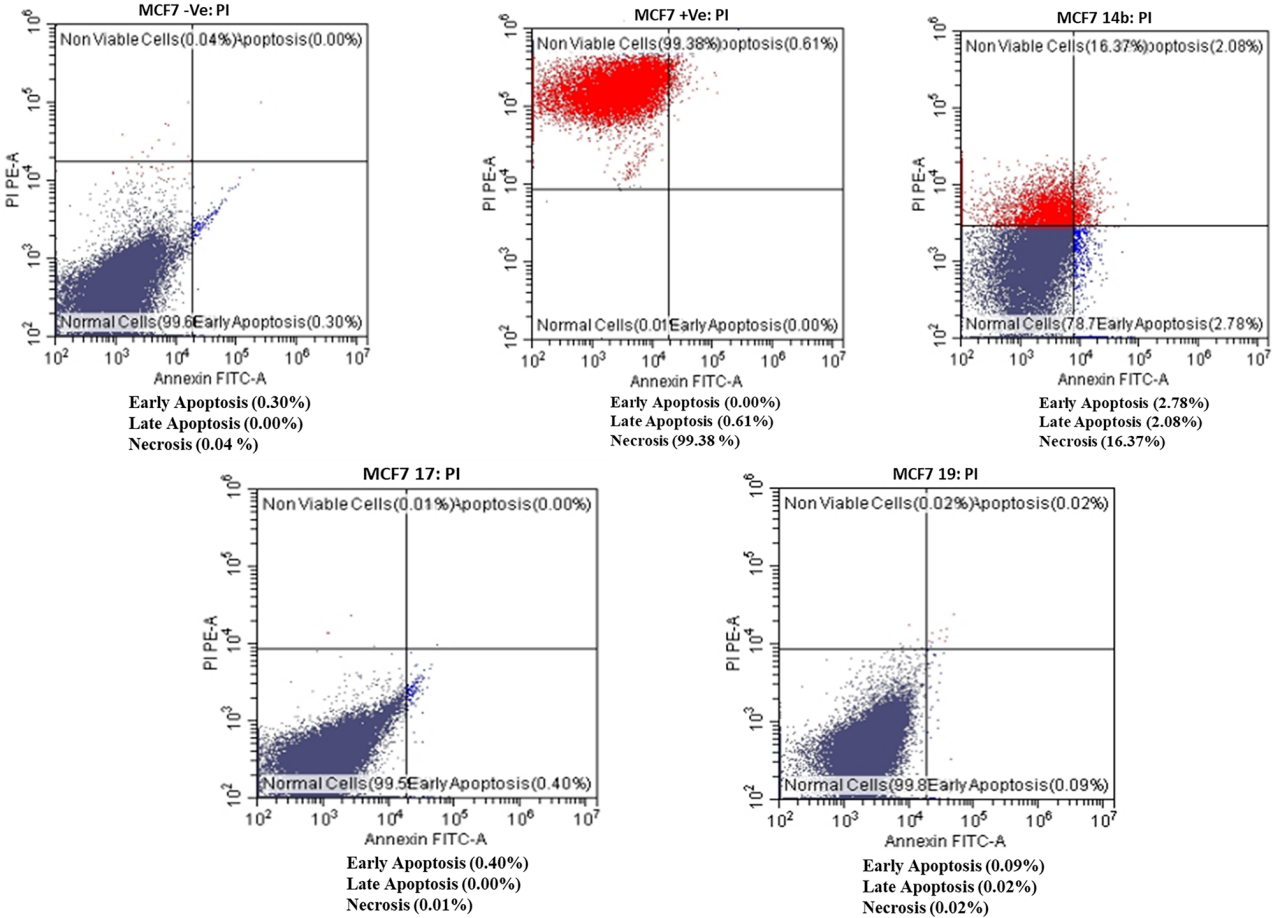
Flow cytometry analysis using Annexin V FITC and propidium iodide (PI) for apoptosis measurements for compound **14b, 17, 19** and **DOX** in MCF cells.

Compound **14b** showed high significant increase in necrosis (16.37%) compared to the normal control (0.04%). In addition, early and late apoptosis were 2.08 and 2.78% respectively when compared with normal control (0.30%). Whereas, compounds **17** and **19**, did not show any significant differences with normal control, but DOX showed highly increasing necrosis (99.83%) when compared with normal control (0.04%) Fig. 9.

### Effects of compounds on HCT116 cells

**Figure 10:**
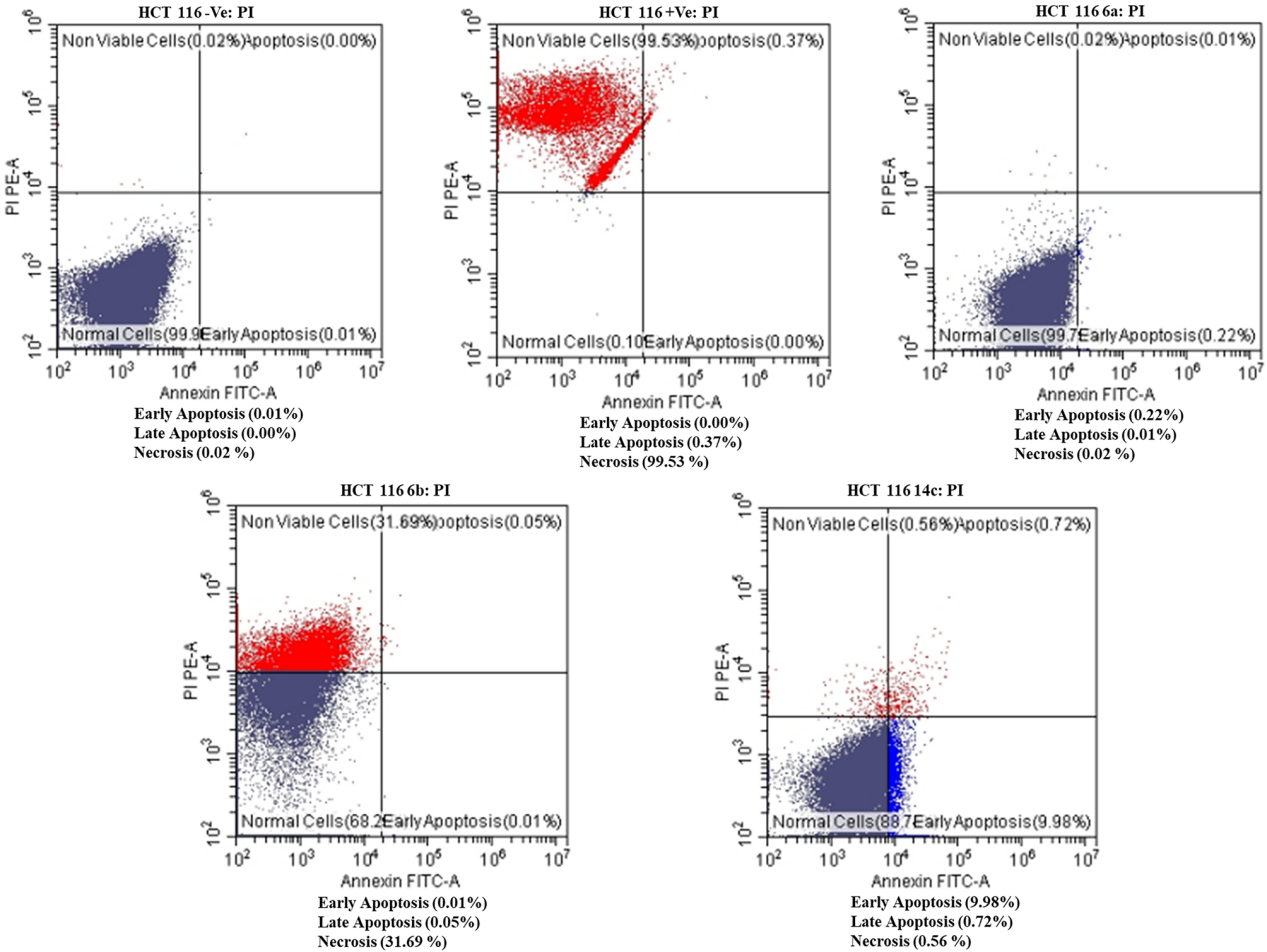
Flow cytometry analysis using Annexin V FITC and propidium iodide (PI) for apoptosis measurements for compound **6a, 6b, 14c** and **DOX** in HCT116 cells.

The apoptotic rate of compounds **6a**, **6b**, **14c** and DOX in colon cancer **(**HCT116**)** was determined using the Annexin V–FITC/PI Double Staining Kit. The results revealed that only compound **6b** and DOX showed a highly significant increase in necrosis (31.69 and 99.53% respectively) when compared with normal control (0.02%). While the apoptotic rate of **14c** in colon cancer (HCT116) recorded no significant differences in necrotic cells (0.56%) when compared with normal control (0.02%) and the early apoptosis was significantly highly increased (9.98%) in these treated cells when compared with normal control (0.01%). Additionally, compounds **6a** did not show significant necrosis in the cells (0.02%) when compared with negative control (0.02%) Fig. 10.

### Effects of compounds 14b and 14c on cell cycle arrest

**Figure 11:**
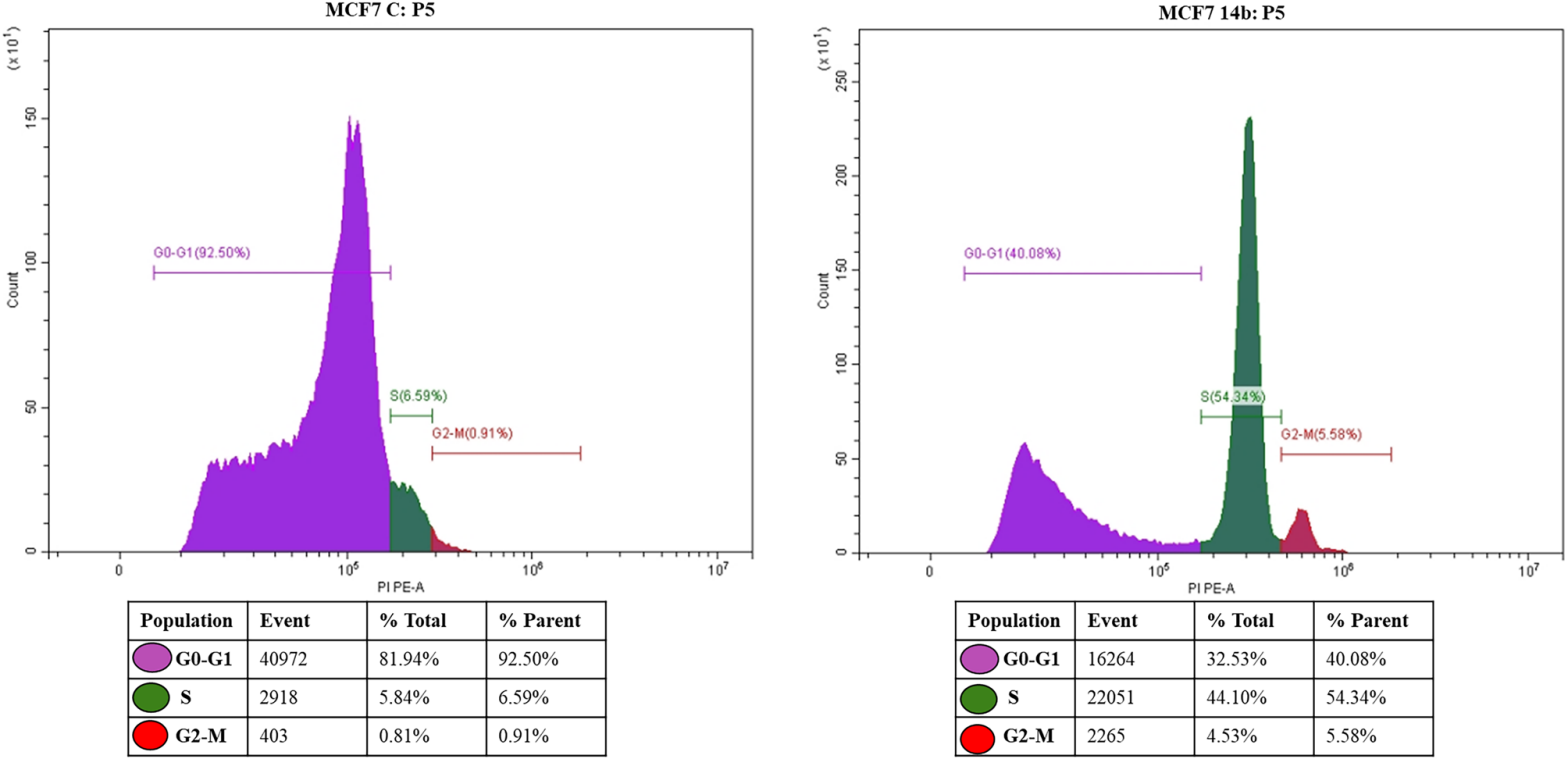
Effect of compound **14b** on cell cycle arrest in MCF cells.

**Figure 12:**
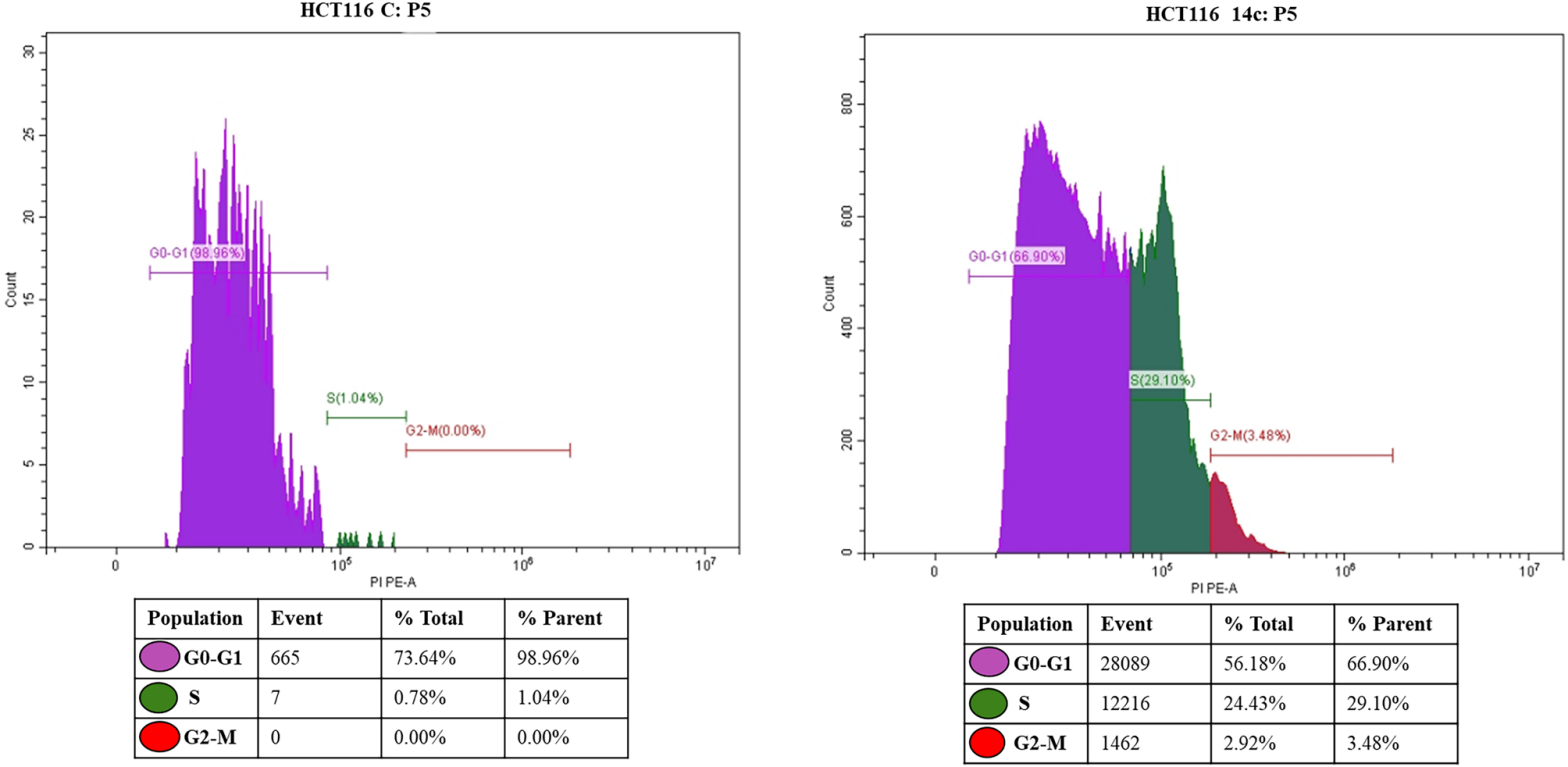
Effect of compound **14c** on cell cycle arrest HCT 116 Cells.

Furthermore, the impact of compounds **14b**, and **14c** on the cell cycle arrest and proliferation of both MCF7 and HCT116 cell lines were investigated after 24 h of treatment utilizing cellular DNA flow cytometry and the untreated cells were used as a negative control for comparison (Figs. 11, 12). The results showed that in the MCF7 control group, the majority of cells were in the G0/G1 phase (81.94%), with only 5.84, and 0.81% in the S and G2- M phase, respectively. Upon treatment with compound **14b**, there was a significant decrease in the G0/G1 phase (32.53%), and a significant increase in the S phase (44.1%). Additionally, there was an increase in the G2-M phase (4.53%) in treated MCF7cells compared to the control (0.81%) Fig. 11.

On the other hand, the study conducted on the HCT116 cell line showed that in the negative control group, 73.64% of HCT116 cells were in the G0-G phase, whereas only 0.78% and 0.00% of cells in S and G2-M phases, respectively. However, upon treatment with compound **14c,** the number of cells in the G0–G1 phase decreased significantly (56.18%) while the number of cells increased significantly in the S phase (24.43%) and G2-M phase (2.92%) as compared to the normal control (Fig. 12).

It is important to note that the G1 phase is the growth phase where everything is ready for DNA synthesis, while the S phase is the synthesis phase, and the G2 phase is the growth and preparation stage for mitosis. An agent that can arrest cell division in cancer cells in either the S or G2 phase is considered an anti-cancer agent. The potency of this agent is measured by the number of cells in the S and G2 phases, and there is an inverse relationship between the potency of anticancer activities and the number of cells in the S and G2 phases.

Furthermore, cell cycle checkpoints in the S and G2 phases of the cell cycle are the major checkpoints and play an important role in cell cycle progress. Based on these findings, we hypothesize that both **14b** and **14c** have anticancer activities against MCF7 and HCT116 cancer cells, respectively. Additionally, our tested compounds upregulated the P53 expression, which causes cell cycle arrest via p21 activation^31^. Therefore, we propose that the resulting cell cycle arrest may be due to the increased expression of P53.

### In silico studies

#### Molecular docking

**Figure 13:**
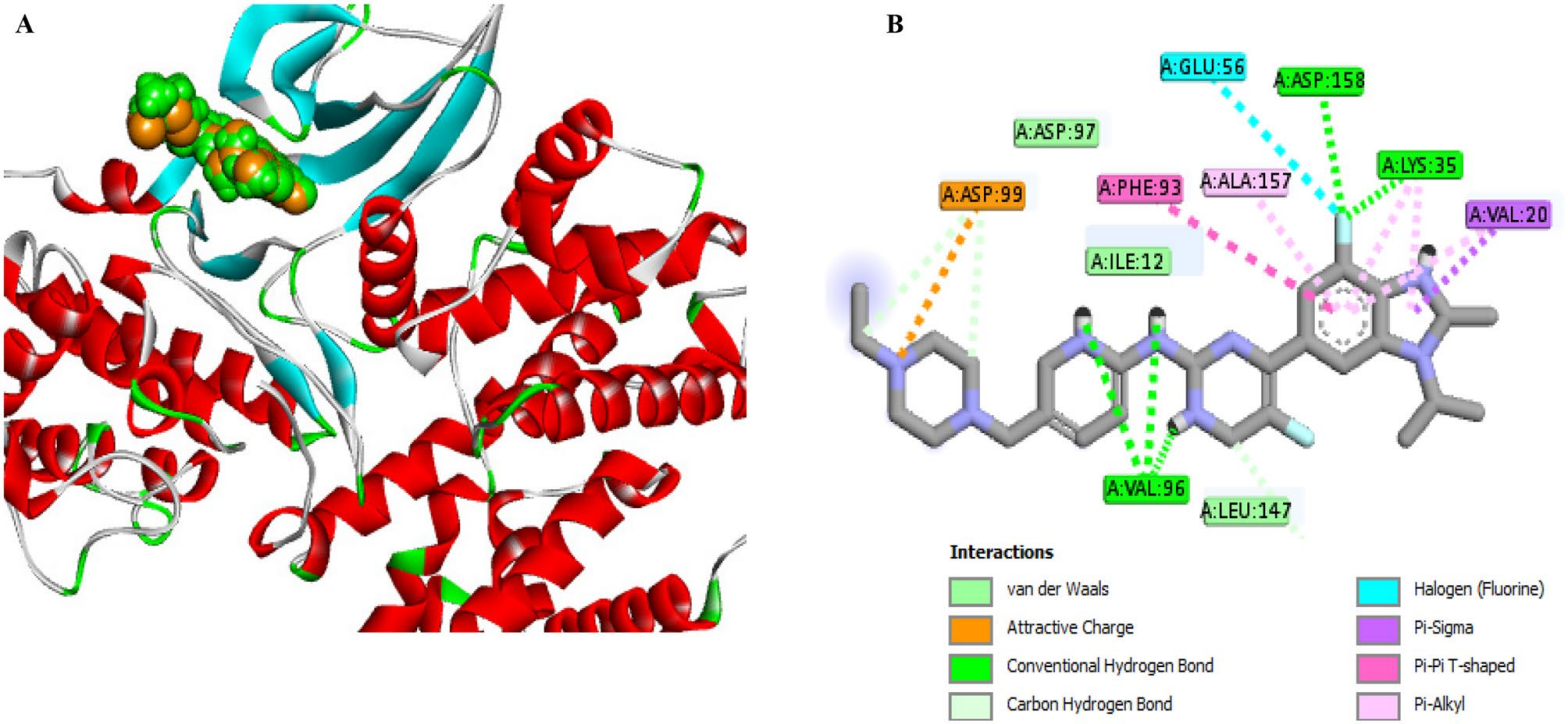
**A** The 3D conformations of the native ligand, **abemaciclib** (green) and re-docked ligand (brown) within the active site of CDK4-Cyclin D3 **(PDB ID: 7SJ3**); showed that they were superimposed in the same position. **B** The 2D conformations of the re-docked **abemaciclib** within the active site of CDK4-Cyclin D3 **(PDB ID: 7SJ3).**

The molecular docking technique was utilized to comprehend the binding interaction of the most active synthesized compounds **6a**, **6b**, **11**, **14b**, **14c**, **17** and **19** with the binding sites of CDK4 (PDB ID:7SJ3). PyRx tools Autodock vina (version 8) were used to execute the docking technique. The lowest energy of binding (LEB) was calculated for each compound, which reflects the binding affinity of the compound. Additionally, hydrogen bonds and hydrophobic bonds such as carbon-hydrogen, van der Waals, Pi-sigma, alkyl, Pi-alkyl, etc. were evaluated^32^. To validate the docking protocol, the co-crystallized ligand was re-docked into the enzyme's active site, and it was found that the re-docked ligand overlapped with the native ligand at the same position with 0 Å RMSD, indicating the reliability of the docking protocol (Fig. 13).

**Figure 14:**
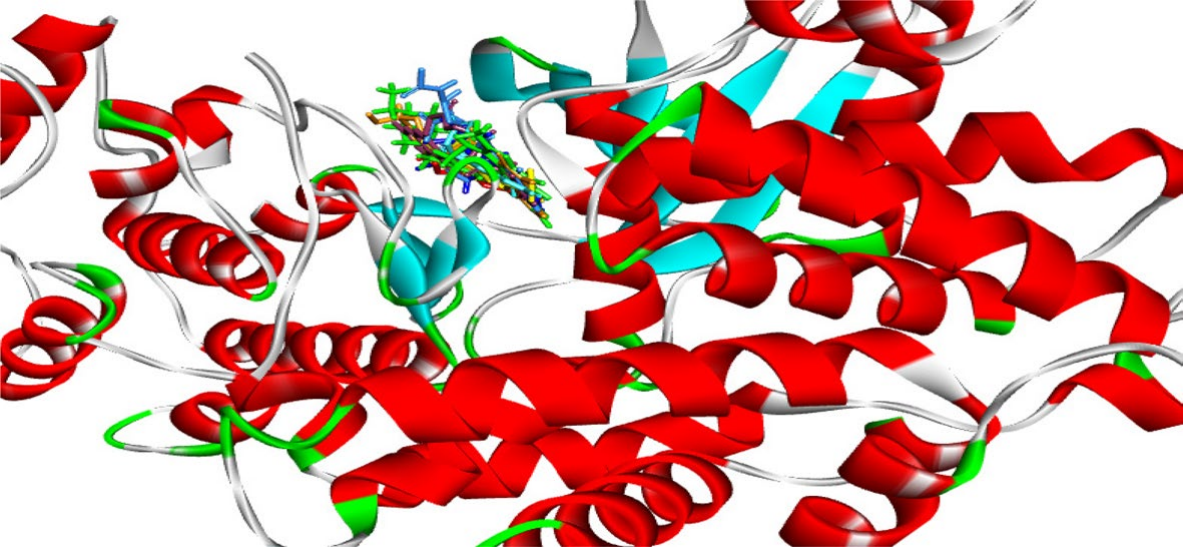
The 3D conformations of the native ligand (green), re-docked ligand (brown) and all docked compounds a within the active site of CDK4-Cyclin D3 **(PDB ID: 7SJ3)**; showed that they were superimposed in the same position.

**Figure 15:**
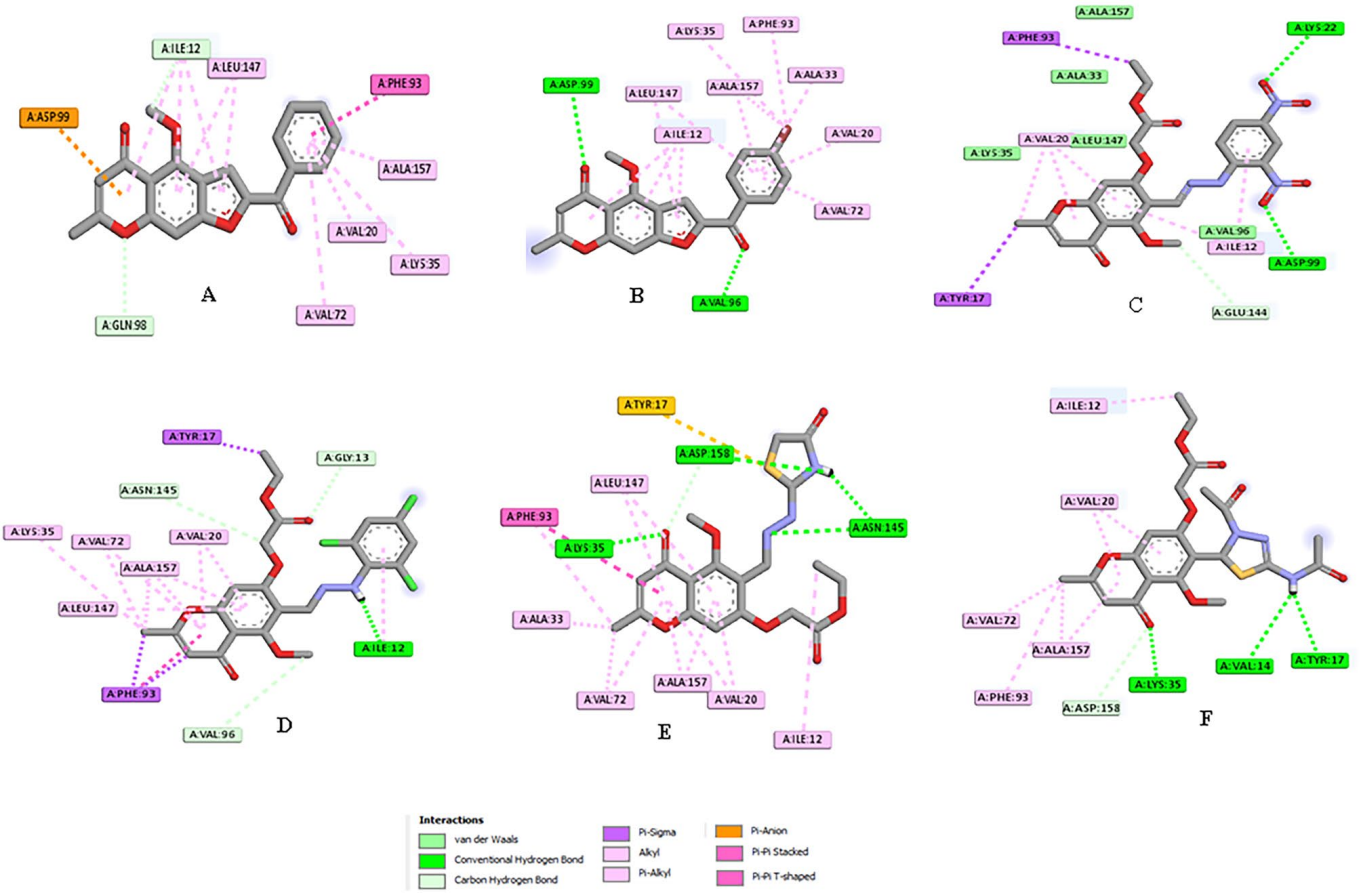
The 2D conformations of all docked compounds within the active site of CDK4-Cyclin D3 **(PDB ID: 7SJ3); A** compound **6a; B** compound **6b; C** compound **14b; D** compound **14c; E** compound **17; F** compound **19.**

The binding affinity and interaction modes of the compounds with the target enzyme are summarized in Table 5. The results showed that all the compounds were able to effectively bind to the active site of CDK4 and displayed good binding energy ranging from -9.8 to 8.7, except compound **11**, (− 7.7 kcal/mol) compared to the native ligand of − 11.3 kcal/mol (Table 5, Figs. 14, 15).

**Table 5:** Molecular docking result of the newly synthesized compounds **6a**, **6b**, **11**, **14b**, **14c**, **17** and **19** inside the CDK4 active pocket (PDB ID: 7SJ3).

Comp. no.	Score (Kcal/mol)	Type of interaction and the amino acid residues involved	
Co-crystalline ligands	− 11.3	Conventional H-bonds Lys35, Val96, Asp158; Carbon H-bonds Asp99, Leu147; Halogen Glu56; Pi-Sigma Val20, Pi-Pi T-shaped Phe93, Pi-Alkyl Val20, Lys35, Ala157, Attractive charge Asp99 and van der Waals Glu194, Ala33, Leu147, Ile12, Asp97	
**6a**	− 9.8	Carbon H-bonds Ile12, Gln98; Pi-anion Asp99, Pi-Pi stacked Phe9, Alkyl and Pi-Alkyl Val20, Val72, Ala33, Phe93, Lys35, Ala157, Ile12, Leu147	
**6b**	− 9.7	Conventional H-bonds Asp99, Val96; Alkyl and Pi-Alkyl Leu147, Ile12, Ala157, Lys35, Phe93, Ala33, Val20, Val72	
**11**	− 7.7	Conventional H-bond Asp99; Carbon hydrogen bond Gly13, Gln98 and Pi-Anion Asp99, Pi-sigma Phe93	
**14b**	− 8.9	Conventional H-bonds (Lys22, Asp99); Carbon H-bond Glu144; Pi-sigma Phe93, Tyr17, Alkyl and Pi-Alkyl Val96, Ile 12 and van der Waals Val20, Ala33, Leu147, Lys35, Ala157	
**14c**	− 8.8	Conventional H-bonds Ile12; Carbon H-bonds Gly13, Asn145, Val96; Pi-sigma Phe93, Tyr17; Pi-Pi T-shaped Phe93, Alkyl and Pi-Alkyl Val20, Val72, Ala157, Leu147, Lys35	
**17**	− 8.9	Conventional H-bonds Asn145, Asp158, Lys35; Carbon hydrogen bond Asp185, Pi-Sulfur Tyr17, Pi-Pi T-shaped Phe93; Alkyl and Pi-Alkyl Ile12, Vl20, Ala157, Phe93, Ala33, Val72, Leu147	
**19**	− 9.1	Conventional H-bonds Tyr17, Val14, Lys35; Carbon hydrogen bond Asp158, Alkyl and Pi-Alkyl Ile12, Ala157, Val72, Phe93, Val20

By analyzing the binding pocket of the re-docked abemaciclib, we have found it displayed multiple interactions including three conventional H-bonds with Lys35, Val96, Asp158 as well as hydrophobic interactions with Asp99, Leu147, Glu56, Val20, Phe93, Lys35, Ala157, Glu194, Ala33, Leu147, Ile12 and Asp97 residues (Table 5, Fig. 13B).

Concerning the new active molecules, compound **6a** showed the best docking score value of -9.8 kcal/mol and stabilized in the active pocket through thirteen hydrophobic interactions with amino acid of CDK4 active site Ile12, Gln98, Asp99, Phe9, Val20, Val72, Ala33, Phe93, Lys35, Ala157, Ile12, and Leu147 residues, respectively (Table 5, Fig. 15A). Whereases, the existence of bromine has had a positive impact on the binding of compound **6b** to the enzyme active pocket. This can be seen through the formation of two hydrogen bonds with the amino acid Asp99, Val96 and thirteen hydrophobic interactions with Leu147, Ile12, Ala157, Lys35, Phe93, Ala33, Val20, and Val72 (Fig. 15B).

On the other hand, the presence of ester and two nitro groups in compound **14b** enhanced the binding interaction with the CDK4 active site which formed two H-bonds (strongest interaction) with Lys22, Asp99 residues as well as established carbon H-bond, Pi-sigma Alkyl and Pi-Alkyl and van der Waals interactions with Glu144, Phe93, Tyr17, Val96, Ile 12 Val20, Ala33, Leu147, Lys35, Ala157 residues (Fig. 15C). Compound **14c** with ester and trichloro phenyl moieties demonstrated one H-bond and eleven hydrophobic interactions (Fig. 15D).

Furthermore, the 4-oxothiazolidine ring in compound **17** played a key role in its stabilization in the active pocket, forming two H-bonds with Asn145, Asp158 and a Pi-Sulfur interaction with Tyr17. Additionally, the carbonyl group of chromone moieties formed an H-bond with the catalytic amino acid Lys35, as well as the ester and chromone moieties established twelve hydrophobic interactions (Fig. 15E).

Finally, compound **19** revealed a good binding energy of − 9.1 kcal/mol and formed a stable complex with the active pocket through the formation of three bonds, one carbon H bond and seven hydrophobic interactions (Fig. 15F).

#### Drug likeliness

The drug-likeness properties of our hit compounds **6a**, **6b**, **11**, **14b**, **14c**, **17** and **19** were studied using free online Swiss ADME software (http://www.swissadme.ch/index.php) and were analyzed according to Lipinski’s rule of five (Ro5) and Veber's rule^33,34^. The result indicated that compounds **6a**, **6b** and **17** were in line with Lipinski’s Ro5 and Veber's rule except compound **17** violated Veber's rule by 1; the TPSA increased the limited value ≤ 140 (Table 6). While compounds **14b**, **14c**, **11**, **19** and doxorubicin violated Lipinski’s Ro5 and Veber rule by 1 or 2 (Table 6).

**Table 6:** Physicochemical properties of the most active synthesized compounds **6a**, **6b**, **11**, **14b**, **14c 17** and **19** using SwissADME online server.

Comp. no.	MW g/mol	Log p	HBA	HBD	TPSA Å^2^	MR	nRB	Drug likeness	
								No. Lipinski violation	No. Veber violation
**6a**	334.32	1.22	5	0	69.65	93.56	3	0	0
**6b**	492.11	2.42	5	0	69.65	108.99	3	0	0
**14b**	500.42	− 0.36	11	1	191.00	131.26	11	2	2
**14c**	513.76	2.67	7	1	99.36	128.65	9	1	0
**11**	443.41	− 0.76	10	0	148.50	111.87	11	1	2
**17**	433.44	0.03	9	1	154.09	115.29	8	0	1
**19**	477.49	− 0.01	9	1	162.04	127.64	10	1	1

*MW* molecular weight, Log *P* lipophilicity (log octanol/water partition coefficient), *HBA* hydrogen bond acceptor, *HBD* hydrogen bond donor, *MR* molar reactivity, *TPSA* topological polar surface area. Drug likeness (Lipinski Pfizer filter) limits are “Yes, drug-like” for MW ≤ 500, Log p (MLOGP) ≤ 4.15, HBA ≤ 10, and HDD ≤ 5. Veber GSK filter for nRB ≤ 10, **TPSA** ≤ 140 Å^2^.

**Figure 16:**
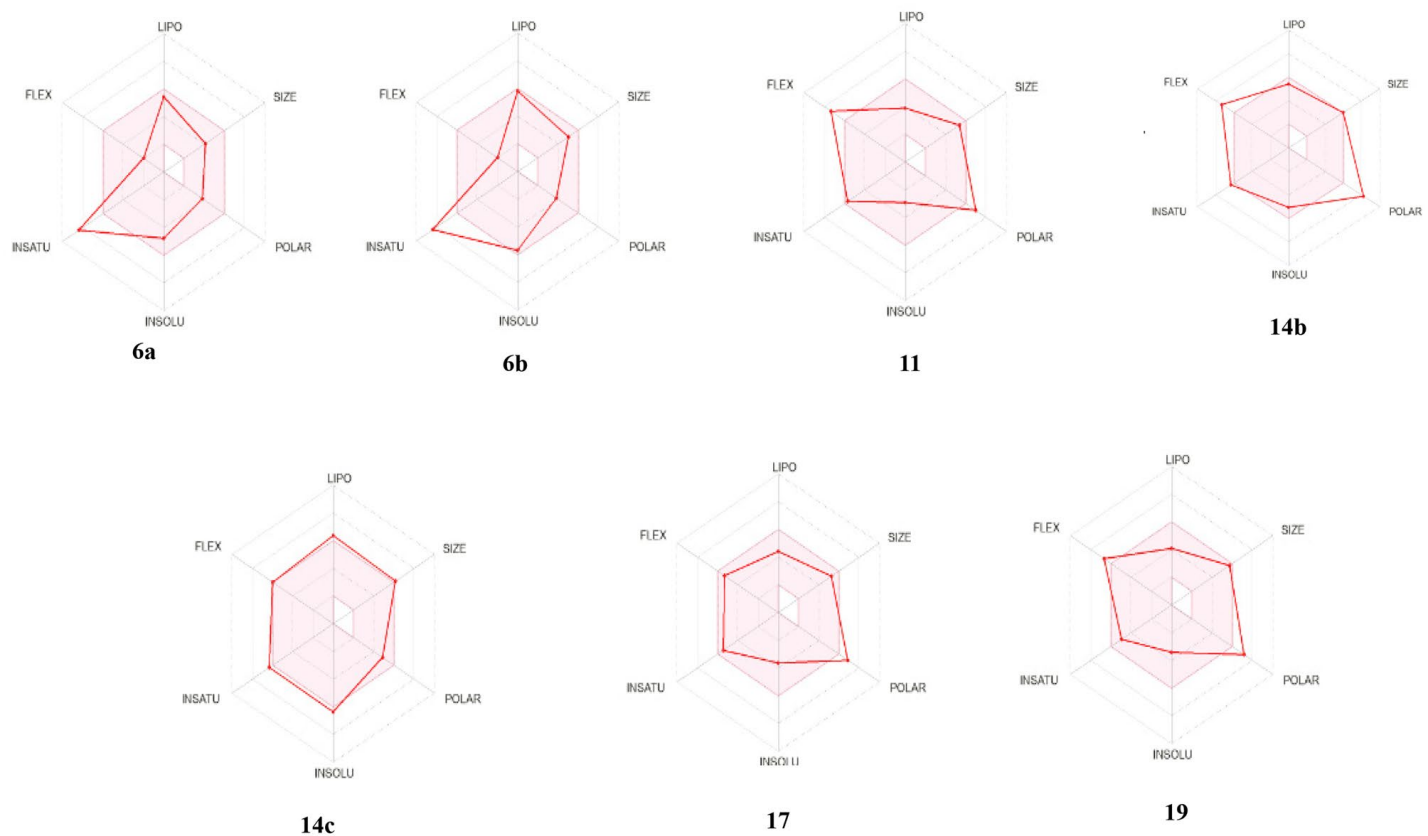
Bioavailability radar chart of the potent chromone congers **6a, 6b, 11, 14b, 14c, 17** and **19**. The ideal value for each oral bioavailability factor was shown in the pink region, and the expected ones for the assessed molecules were shown as red lines the colored zone is the typical physiochemical space for oral bioavailability.

Based on the bioavailability radar chart, it was observed that compound **14c** falls within the optimal range (pink area) for the six major variables, namely lipophilicity, size, polarity, solubility, saturation, and flexibility. This result indicates a good likelihood of oral bioavailability for this compound. Conversely, compounds **6a** and **6b** are not expected to be orally bioavailable as they were far from the ideal saturation range. Moreover, compounds **11**, **14b**, **17** and **19** were found to be slightly outside the ideal polarity and flexibility ranges (Fig. 16).

On the other hand, The ADMET profile of our hit compounds **6a**, **6b**, **11**, **14b**, **14c**, **17** and **19** was evaluated by Admetsar2 (http://lmmd.ecust.edu.cn/admetsar2) Table 7.

**Table 7:** Prediction of some of the ADMET end points of the most active synthesized compounds **6a**, **6b**, **11**, **14b**, **14c** **17** and **19** using the AdmetSAR server.

Parameters	6a	6b	11	14b	14c	17	19
Absorption							
Water solubility^a^ [Log S (log mol/L)]	− 3.21	− 3.76	− 3.5	− 4.07	− 4.49	− 3.51	− 3.51
Human intestinal absorption	+	+	+	+	+	+	+
Caco-2 permeability	+	+	–	–	–	–	–
Human oral bioavailability	+	+	–	+	+	+	–
Distribution							
Blood–Brain Barrier	+	+	+	+	+	+	–
P-glycoprotein inhibitor	+	+	+	+	+	+	+
P-glycoprotein substrate	–	–	+	+	–	+	+
Plasma protein binding (100%)	0.797	1.06	0.77	1.004	0.87	0.82	0.76
Metabolism							
CYP3A4 substrate	–	+	+	+	+	+	+
CYP2C9 substrate	–	–	–	–	–	–	–
CYP2D6 substrate	–	–	–	–	–	–	–
CYP3A4 inhibition	+	+	–	+	+	+	+
CYP2C9 inhibition	–	+	–	+	+	+	+
CYP2C19 inhibition	+	+	+	+	+	+	+
CYP2D6 inhibition	+	+	–	–	–	–	–
CYP1A2	+	+	–	+	+	–	–
CYP inhibitory promiscuity	+	+	+	+	+	+	+
Excretion							
Renal OCT2 inhibitor	–	–	–	–	–	–	–
Toxicity							
Carcinogenesis (binary)	–	–	–	–	–	–	–
AMES toxicity	–	+	–	+	–	–	–
Human Ether-a-go-go-Related Gene inhibition	+	+	+	+	+	+	–
Acute oral toxicity C	III	III	III	III	III	III	III
Acute oral toxicity (mol/kg)	2.33	2.57	3.46	2.6	2.12	3.32	1.97
Hepatotoxicity	–	–	–	+	+	–	–
Skin sensitization	–	–	–	–	–	–	–

^a^Log S: solubility: log S > − 10 (insoluble); − 10 to − 6 (weakly soluble); − 6 to − 4 (moderately soluble), from − 4 to − 2 (soluble); -2–0 (extremely soluble); and greater than zero (very soluble).

According to the predicted data, the tested compounds were found to be absorbed in the human intestine, water-soluble and showed oral bioavailability except for compounds **11** and **19**. Only compounds **6a** and **6b** were predicted to have Caco2 permeability. Moreover, all hits had a positive effect on P-gp inhibitors and could break the blood–brain barrier, which could be a promising avenue for future research in exploring bioactive molecules targeting nervous system diseases except compound 9 (Table 7).

The molecule's ability to inhibit or substrate cytochrome P450 (CYP450) served as a representative of the metabolism criteria. Some isoforms are responsible for 90% of the oxidative stress, while substrates of CYP2C9, 2D6, 3A4, and inhibitors of CYP1A2, 2D6, 2C9, 2C19, 3A4 indicate the likelihood of Drug-drug interaction phenomenon with other drugs. In our records, the tested compounds displayed inhibition effects on some CYP450 isoforms^35^ (Table 7).

The OCT2 (organic cation transporter 2) is the first step in the renal secretion of many cationic drugs, and its inhibitors may alter the way drugs accumulate in the kidney and cause nephrotoxicity^36^. None of the tested compounds inhibited OCT2. Ultimately, the anticipated toxicity profile of the investigated compounds showed that the majority of them had negative skin sensitization and no Ames negative hepatotoxicity (Table 7).

## Conclusion

Twenty new chromone derivatives have been synthesized and their cytotoxic activity was tested against human breast cancer (MCF-7), colon (HCT-116) and liver cancer cell lines (HepG2) as well as normal skin fibroblast cells (BJ1). The result displayed that Compounds **14b**, **17**, and **19** showed cytotoxic activity against MCF-7, whereas compounds **6a**, **6b**, **11** and **14c** exhibited highly potent activity toward HCT-116 cancer cell lines. The potential cytotoxic effects of these compounds may be due to their ability to induce DNA fragmentation in cancer cell lines, down-regulate the expression level of CDK4 as well as the anti-apoptotic gene Bcl-2 and up-regulate the expression of the pro-apoptotic genes P53 and Bax. Additionally, compounds **14b** and **14c** showed a dual mechanism of action via apoptosis and cell cycle arrest induction. The molecular docking study was carried out to understand the binding interaction of the most active synthesized compounds with the binding sites of CDK4. Compounds **6a**, **14b**, **17**, and **19** showed good binding energy and formed stable complexes with the enzyme active pocket. Moreover, the bioavailability radar chart, showed that compound **14c** falls within the optimal range for the six major variables, namely lipophilicity, size, polarity, solubility, saturation, and flexibility.

## Materials and methods

### Chemistry

#### General information

All reagents and solvents were of commercial grade. Visnagin (Sigma-Aldrich ChemieGmeH,Taufkirchen, Germany). Melting points were determined on the digital melting point apparatus (Electro thermal 9100, Electro thermal Engineering Ltd., serial No. 8694, Rochford, United Kingdom) and are uncorrected. The reaction progress was monitored by thin-layer chromatography (TLC) using silica gel plates (POLYGRAM SILG/UV254, 0.20 mm), which were visualized under UV light 254 and 365 nm. The IR spectra were detected utilizing the Brukur-5000 FTIR spectrometer. The ^1^H and ^13^C NMR spectra were recorded using a JEOL-ECA-50 NMR instrument at 500 and 125 MHz, respectively, using TMS as the internal standard, National Research Center, Egypt. Hydrogen coupling patterns are described as (s) singlet, (d) doublet, (t) triplet, (q) quartetand (m) multiple. Chemical shifts were defined as parts per million (ppm) relative to the solvent peak. Mass spectra (EI) were identified on Finnegan MatSSQ 7000 mode: EI, 70Ev (Thermo Inst. Sys. Inc., USA). 6-formyl-7-hydroxy-5-methoxy-2-methyl chromone (**2**), and ethyl 2-((6-formyl-5-methoxy-2-methyl-4-oxo-4H-chromen-7-yl)oxy)acetate (**8**) were prepared according to the reported methods^23,24^.

##### General procedure for the preparation of 7-methoxy-10-methyl-3,4-dihydro-2H-3,6-epoxy[1,5]dioxocino[3,2-g]chromen-8(6H)-one (3)

A mixture of (**2**, 0.23 g, 0.001 mol), epichlorohydrin (1 mL) and triethyl amine (0.5 mL) was heated under reflux for 3 h. (TLC, n-hexane/ethyl acetate, 3:1). The reaction mixture was cooled to room temperature followed by quenching with ice water. The organic layer was extracted using ethyl acetate, and dried over anhydrous sodium sulphate. The solvent was evaporated under a vacuum; the product was obtained as oil pasty and was produced in an extremely pure state that required no further purification.

Brown paste oil, m.p.65–7 °C; yield**:**89%; IR (cm^−1^) 1650 (C=O), 1582 (C=C), 1167,1112 (C–O); ^1^H NMR (500 MHz, DMSO-*d*6) δ 6.92 (s, 1H, CH pyranone ring), 6.51 (s, 1H, CH benzene ring), 6.02 (s, 1H, Ha), 4.76 (m, 1H, Hb), 4.42 (d, *J* = 12.5 Hz, 1H, Hc), 4.12 (d, *J* = 6.2 Hz, 1H, Hd), 3.94 (d, *J* = 12.8 Hz, 1H, He), 3.78 (t, *J* = 10.2, 6.5 Hz, 1H, Hf), 3.70 (s, 3H, OCH_3_), 2.25 (s, 3H, CH_3_).^13^C NMR (500 MHz, DMSO-D6) δ175.66, 164.96, 161, 79, 158.41, 158.00, 125.11, 113.29, 111.75, 106.34, 98.15, 75.42, 75.31, 66.27, 63.89, 19.80; m/z: 290 (M^+^) for C_15_H_14_O_6_ (290.27).

##### General procedure for the preparation of (E)-6-(((5-chloro-2-nitrophenyl)imino)methyl)-7-hydroxy-5-methoxy-2-methyl-4H-chromen-4-one (4)

A mixture of **(2**, 0.23 g, 0.001 mol) and 2-nitro-5-chloro aniline (0.001 mol) in absolute ethanol containing a few drops of glacial acetic acid was heated under reflux for 2 h. The solid formed on hot was filtered off, and air dried. The product was pure enough and used in the second step without further purification.

Yellow ppt, m.p.154–6 °C; yield: 95%; %; IR (cm^−1^) 3410 (OH), 1705 (C=O), 1645 (C=N), 1585 (C=C); ^1^H NMR (500 MHz, DMSO-*d*6) δ 14.52 (s, 1H, OH), 9.20 (s, 1H, CH=N), 8.47 (s, 1H, Ar–H), 8.14 (dd, 1H, *J* = 45.5, 12.3 Hz, Ar–H), 7.85 (d, 1H, *J* = 8.6 Hz, Ar–H), 6.75 (s, 1H, CH pyranone ring), 6.03 (s, 1H, CH benzene ring), 3.92 (s, 3H, OCH_3_), 2.27 (s, 3H, CH_3_).

##### General procedure for the preparation of (E)-6-(((2-chloro-5-nitrophenyl)imino)methyl)-5-methoxy-2-methyl-7-(oxiran-2-ylmethoxy)-4H-chromen-4-one (5)

A solution of Schiff's base **4** (0.01 mol) and epichlorohydrin (0.015 mol) in dry acetone (10 mL) containing anhydrous potassium carbonate (0.02 mol) was heated under reflux for 6 h. After the reaction was completed (as monitored by TLC, n-hexane/ethyl acetate 3:1), the mixture was cooled to room temperature and quenched with ice water. The organic layer was extracted using ethyl acetate, and dried over anhydrous sodium sulphate. The solvent was evaporated under vacuum and the product was crystallized from methanol.

Brown oil; yield: 75%; IR (cm^−1^) 1731 (C=O), 1648 (C=N), 1598 (C=C), 1190, 1246, 1128, 1086, 1032 (C–O); ^1^H NMR (500 MHz, DMSO-*d*6) δ 7.54 (s, 1H, Ar–H); 7.04 (d, *J* = 1.8 Hz, 1H, Ar–H); 6.93 (s, 1H,Ar–H); 6.60 (d,* J* = 9.2, 2.0 Hz, 1H, Ar–H); 6.52 (s, 1H, CH pyrone ring); 6.04 (s, 1H CH benzene ring); 4.77 (d, *J* = 5.5 Hz, 1H, CH_2_); 4.42 (dd, *J* = 13.1, 2.0 Hz, 1H, CH_2_); 4.13 (d, *J* = 7.0 Hz, 1H, CH); 3.94 (d, *J* = 13.1 Hz, 1H, CH_2_), 3.79 (t, *J* = 6.5 Hz, 1H, CH_2_); 3.71 (s, 3H, OCH_3_), 2.26 (s, 3H, CH_3_); ^13^C NMR(125 MHz, DMSO-*d*6) δ 175.67, 164.98, 161.83, 158.45, 158.03, 128.11, 125.14, 118.19, 116.09, 113.33, 111.78, 106.37, 98.16, 75.44, 75.30, 66.28, 63.91, 19.83; m/z:444/446 (M^+^/M^+^ + 2) for C_21_H_17_ClN_2_O_7_ (444.82).

##### General procedure for the preparation of 2-substituted-benzoyl-4-methoxy-7-methyl-5H-furo[3,2-g]chromen-5-one (6)

A mixture of **2** (0.01 mol), phenacyl bromide derivatives (0.01 mol) and anhydrous potassium carbonate (0.02 mol) in dry acetone (10 mL) were refluxed for 6–10 h. After the reaction was completed as monitored by TLC (n-hexane/ethyl acetate 3:1), the mixture was poured onto ice water. The Precipitate formed was filtered off, washed with water, air dried re-crystallized from methanol. In the case of compound **6a,** the product was obtained after the mixture was separated using ethyl acetate and then dried over anhydrous sodium sulphate. The solvent was evaporated under vacuum and the product was obtained in an extremely pure state that required no further purification.

**2-benzoyl-4-methoxy-7-methyl-5H-furo[3,2-g]chromen-5-one (6a):** off white ppt, m.p.185–7 °C; yield: 90%; IR (cm^−1^) 1730, 1655 (C=O), 1608, 1574 (C=C), 1198,1148, 1107, 1036 (C–O–C); ^1^H NMR(500 MHz, DMSO-*d*6) δ 7.98 (d, *J* = 7.3 Hz, 2H, Ar–H), 7.95 (s, 1H, CH furan ring), 7.72–7.69 (m, 1H, Ar–H), 7.58 (t, *J* = 7.2 Hz, 2H, Ar–H), 7.52 (s, 1H, CH pyrone ring), 6.00 (s, 1H, CH benzene ring), 4.12 (s, 3H, OCH_3_), 2.28 (s, 3H, CH_3_). ^13^C NMR(125 MHz, DMSO-*d*6) δ 183.33 (C=O), 176.66 (C=O), 164.68, 158.17, 156.33, 152.01, 136.95, 133.80, 129.74, 129.37, 116.48, 110.98, 95.41, 62.15 (OCH_3_), 19.83( CH_3_); m/z: 334 (M^+^, 8.67) for C_20_H_14_O_5_ (334.33).

**2-(4-Bromobenzoyl)-4-methoxy-7-methyl-5H-furo[3,2-g]chromen-5-one (6b):** off white ppt, m.p. 213–5 °C; yield: 80%; IR (cm^−1^) 1725, 1653 (C=O), 1618, 1588 (C=C), 1187, 1135, 1091, 1080 (C–O–C); ^1^H NMR(500 MHz, DMSO-*d*6) δ 7.99 (m, 3H, Ar–H), 7.64–7.63 (m, 2H, Ar–H), 7.51 (s, 1H, CH benzene ring), 6.01 (s, 1H, CH pyranone ring), 4.13 (s, 3H, OCH_3_), 2.28 (s, 3H, CH_3_); ^13^C NMR (125 MHz, DMSO-*d*6) δ 182.18, 176.65, 164.68, 158.27, 158.21, 156.40, 151.53, 138.71, 135.58, 131.65, 129.48, 116.98, 116.88, 111.01, 95.35, 62.12, 19.83; m/z: 411/413 (M^+^/M^+^ + 2) for C_20_H_13_ BrO_5_ (413.22).

##### General procedure for the preparation of (Z)-5-((7-hydroxy-5-methoxy-2-methyl-4-oxo-4H-chromen-6-yl)methylene) thiazolidine-2,4-dione (7)

To a solution of **2** (1 mmol) and thiazolidine-2,4-dione (0.13 g, 1 mmol) in absolute ethanol (10 mL), a few drops of piperidine were added and the mixture was stirred under reflux for 12 h. The progress of the reaction was monitored by TLC (n-hexane/EtOAc, 3:1). The mixture was cooled to room temperature and quenched with ice water. The Precipitate formed was filtered off, washed with water, air-dried and re-crystallized from methanol.

Yellow ppt, m.p.271 dec.; yield: 60%. IR (cm^−1^) 3465 (OH); 3289 (NH);1694, 1614 (3C=O), 1588, 1517 (C=C) & 1186,1163, 1084, 1065 (C–O–C). ^1^H NMR (500 MHz, DMSO-*d*6) δ 12.10 (s, 1H, OH, exchangeable D_2_O), 9.04 (s, 1H, NH, exchangeable D_2_O), 7.49 (s, 1H, CH=C), 6.73 (s, 1H, CH pyranone ring), 6.01 (s, 1H, CH benzene ring), 3.88 (s, 3H, OCH_3_), 2.26 (s, 3H, CH_3_). m/z: 333 (M^+^) for C_15_H_11_NO_6_S (333.31).

##### General procedure for the preparation of ethyl 2-((6-formyl-5-methoxy-2-methyl-4-oxo-4H-chromen-7-yl)oxy)acetate (8)

A solution of **1** (1 mmol), ethyl bromoacetate (1 mmol) in dry acetone (10 mL) containing anhydrous potassium carbonate (1 mmol) was refluxed for 12 h (TLC, n-hexane/ethylacetate 3:1). The organic layer was extracted using ethyl acetate, dried over anhydrous sodium sulphate. The solvent was evaporated under vacuum and a white solid was obtained in an extremely pure state and used without further purification.

Off white ppt, m.p.85–92 °C; yield: 90%; 1H NMR (500 MHz, DMSO-*d*6) δ 10.28 (s, 1H, CHO), 7.00 (s, 1H, CH pyranone ring), 6.07 (s, 1H, CH benzene ring ), 5.01 (s, 2H, OCH_2_), 4.15 (dd, *J* = 12.2, 7.2 Hz, 2H, COOCH_2_), 3.80 (s, 3H, OCH_3_), 2.27 (s, 3H, CH_3_), 1.20 (t, 3H, CH_3_).

##### General procedure for the preparation of ethyl 2-((5-methoxy-6-(methoxymethyl)-2-methyl-4-oxo-4H-chromen-7-yl)oxy) acetate (9)

To a solution of **8** (1.44 mmol) in methanol (10 mL) was added NaBH_4_ (30 mg, 72 mmol) at 5–0 °C. The reaction was stirred for 15 min at the same temperature, then left stirring for 2 h. at room temperature. After all, compound **8** was consumed as indicated by TLC (n-hexane/ethyl acetate 3:1), and the reaction mixture was quenched by adding 2 mL of cold water, followed by aqueous 5% HCl until the solution became acidic to pH paper. The mixture was extracted with ethyl acetate and the organic layer was dried over anhydrous sodium sulphate. The solvent was evaporated under vacuum and the solid formed was collected and recrystallized from ethanol.

Brown ppt, m.p. 99–101; yield:63%; IR (cm^−1^) 3195 (OH), 1734, 1654 (C=O), 1599 (C=C), 1186, 1120, 1085, 1011 (C–O–C);^1^H NMR (500 MHz, DMSO-d6) δ 6.86 (s, 1H, CH pyrone ring); 5.98 (s, 1H, CH benzene ring), 4.50 (s, 2H, CH_2_O), 4.14 (s, 2H, CH_2_O), 3.74 (s, 3H, OCH_3_), 3.72 (s, 2H, CH_2_CO), 2.25 (s, 3H, CH_3_); 2.25 (s, 1H, OH exchangeable D_2_O), 1.03 (t, 3H, CH_3_); m/z: 322 (M^+^) for C_16_H_18_O_7_ (322.31).

##### General procedure for the preparation of (E)-ethyl 2-((6-((2-(2-cyanoacetyl)hydrazono)methyl)-5-methoxy-2-methyl-4-oxo-4H-chromen -7-yl)oxy)acetate (10)

A mixture of **8** (0.01 mol) and 2-cyanoacetic acid hydrazide and (0.01 mol) in absolute ethanol (15 mL) containing a few drops of glacial acetic acid was serried under reflux for 2 h. The solid formed on hot was filtered off, washed with ethanol, and air dried. The title compounds were obtained in an extremely pure state that required no further purification.

Off white ppt, m.p. 237–9; yield**:** 92%; IR (cm^−1^) 3180 (NH), 2262 (CN), 1742, 1679, 1665 (C=O), 1607 (C=N), 1594 (C=C), 1190, 1164, 1130, 1109, 1086 (C–O–C); 1H NMR (500 MHz, DMSO-d6) δ 11.73 (s, 1H, NH, exchangeable D_2_O), 8.23 (s, 1H, CH=N), 6.96 (s, 1H, H-pyranone), 6.04 (s, 1H, H-benzene ring), 4.97 (s, 2H, OCH_2_), 4.17–4.13 (m, 4H, COOCH_2_, CH_2_CN), 3.77 (s, 3H, OCH_3_, 2.27(s, 3H, CH_3_), 1.21 (t, *J* = 6.9 Hz, 3H, CH_3_). ^13^C NMR (125 MHz, DMSO-*d*6) δ 175.45, 168.33, 165.24, 164.66, 160.30, 159.80, 159.57, 142.10, 138.94, 116.56, 114.83, 111.83, 100.05, 98.33, 66.25, 63.21, 61.47, 24.79, 19.75, 14.53; m/z: 401.1 (M^+^) for C_19_H_19_ N_3_O_7_ (401.4).

##### Ethyl (E)-2-((6-(3-(2-acetylhydrazinyl)-2-cyano-3-oxoprop-1-en-1-yl)-5-methoxy-2-methyl-4-oxo-4H-chromen-7-yl)oxy)acetate (11)

A mixture of **8** (0.01 mol) and 2'-acetyl-2-cyanoacetohydrazide (0.01 mol) in absolute ethanol (15 mL) containing a few drops of triethyl amine was serried under reflux for 2 h. The solid formed on hot was filtered off, washed with ethanol, and air dried. The title compounds were obtained in an extremely pure state that required no further purification.

White ppt, m.p. 211–3; yield: 79%; IR (cm^−1^) 3299, 3241 (NH), 2210 (CN), 1741, 1709, 1697, 1658 (C=O), 1598 (C=N), 1506 (C=C), 1189, 1167, 1127, 1088, 1038 (C–O–C); 1H NMR (500 MHz, DMSO-d6) δ 10.44, 9.93 (2 s, 2H, 2NH, exchangeable D_2_O), 8.06 (s, 1H, CH=C), 7.08 (s, 1H, H-pyranone), 6.10 (s, 1H, H-benzene ring), 5.02 (s, 2H, OCH_2_), 4.17–4.15 (m, 2H, COOCH_2_), 3.71 (s, 3H, OCH_3_), 2.29 (s, 3H, CH_3_), 1.88 (s, 3H, CH_3_), 1.20 (t, *J* = 7.1 Hz, 3H, 2CH_3_). ^13^C NMR (125 MHz, DMSO-*d*6) δ 175.40, 168.87,168.08, 165.02, 160.79, 159.42, 144.17, 138.94, 115.20, 112.85, 112.44, 111.88, 99.98, 98.47, 66.12, 63.17, 61.52, 21.08, 19.81, 18.85, 14.53; m/z 443.1 (M +) for C_21_H_21_N_3_O_8_ (443.4).

##### General procedure for the preparation of (E)-ethyl 2-((5-methoxy-2-methyl-6-((3-methyl-5-oxo-isoxazol-4(5H)-ylidene) methyl) -4-oxo-4H-chromen-7-yl)oxy)acetate (12)

A mixture of ethyl acetoacetate (0.01 mol), hydroxylamine hydrochloride (0.01 mol), and anhydrous sodium acetate (0.01 mol) in absolute ethanol (5 mL) was heated under reflux for 10 min, then **8** was added, and the mixture was further refluxed until the reaction was completed (monitored by TLC). The product formed on hot was filtered off, air dried and re-crystallized from ethanol.

Brown ppt, m.p. 121–3; yield: 65%; IR (cm^−1^) 1710, 1650 (C=O), 1618 (C=N), 1589 (C=C), 1209, 1167, 1112, 1086 (O–C–O); 1H NMR (500 MHz, DMSO-d6) δ 7.08 (s, 1H, CH=C), 6.62 (s, 1H, CH pyrone ring); 6.10 (s, 1H, CH benzene ring), 5.02 (s, 2H, CH_2_O), 4.17 (q, 2H, CH_2_O), 3.71 (s, 3H, OCH_3_), 3.15 (s, 3H, CH_3),_ 2.29 (s, 3H, CH_3),_ 1.20 (t, 3H, CH_3_); m/z: 401(M^+^) for C_20_H_19_NO_8_ (401.37).

##### General procedure for the preparation of ethyl 2-((6-(1H-benzo[d]imidazol-2-yl)-5-methoxy-2-methyl-4-oxo-4H-chromen-7-yl)oxy) acetate (13)

A mixture of **8** (0.5 mmol) and *o*-phenylenediamine (0.5 mmol) was thoroughly mixed in DMF (2 mL), and then *p*-TsOH (0.1 mmol) was added. The solution was heated and stirred at 80 °C for.

appropriate time (monitored by TLC). When the reaction was finished, the solution was cooled to room temperature The reaction mixture was added dropwise with vigorous stirring into a mixture of Na_2_CO_3_ (0.1 mmol) and cold H_2_O (20 mL). The solid precipitated was collected by filtration, washed with water, air dried and re-crystallized from ethanol.

Black ppt, m.p. 129–31; yield:71%; IR (cm^−1^) 3210 (NH), 1672 (C=O), 1622 (C=N), 1589 (C=C), 1189, 1155, 1096 1061 (O–C–O);1H NMR (500 MHz, DMSO-d6) δ 12.15 (s, 1H, NH, exchangeable D_2_O), 8.14–7.07 (m, 4H, Ar–H), 6.78 (s, 1H, CH pyrone ring); 6.08 (s, 1H, CH benzene ring), 4.99 (s, 2H, CH_2_O), 4.15–4.13 (m, 5H, CH_2_O, OCH_3_), 2.28 (s, 3H, CH_3_), 1.19 (t, *J* = 7.1 Hz, 3H, CH_3_); m/z: 408 (M^+^) for C_22_H_20_N_2_O_6_ (M.wt 408.41).

##### General procedure for the preparation of hydrazone derivatives 14a–c

A mixture of **8** (0.01 mol) and hydrazine derivatives (0.01 mol) in absolute ethanol containing a few drops of glacial acetic acid was heated under reflux for 3–4 h. The solid formed was collected by filtration, washed with water, air-dried and re-crystallized from n-hexane–ethyl acetate (3:1).

##### Ethyl (E)-2-((5-methoxy-2-methyl-6-((2-methylhydrazono)methyl)-4-oxo-4H-chromen-7-yl)oxy)acetate (14a)

Yellow ppt, m.p. 102–4; yield: 78%; IR (cm^−1^) 3097 (NH), 1731, 1688 (C=O), 1648 (C=N), 1589 (C=C), 1182, 1122, 1086, 1032 (O–C–O); ^1^HNMR (500 MHz, DMSO-d6) δ 8.10 (s, 1H, CH=N), 6.72 (s, 1H, CH pyranone ring); 6.20 (s, 1H, CH benzene ring), 5.08 (s, 2H, CH_2_O), 4.46 (s, 3H, OCH_3_), 3.33 (d, *J* = 3.7 Hz, 2H, CH_2_); 4.46 (s, 1H, NH exchangeable D_2_O); 4.16 (s, 3H, CH_3_), 2.32 (s, 3H, CH_3_), 1.20 (t,* J* = 6.8 Hz, 3H, CH_3_);m/z: 348 (M^+^) for C_17_H_20_N_2_O_6_ (348.36).

##### Ethyl (E)-2-((6-((2-(2,4-dinitrophenyl)hydrazono)methyl)-5-methoxy-2-methyl-4-oxo-4H-chromen-7-yl)oxy)acetate (14b):

Orange ppt, m.p. 197–9; yield: 95%; IR (cm^−1^) 3285 (NH), 1727, 1705 (C=O), 1651 (C=N), 1604 (C=C), 1185, 1126, 1071, 1049 (O–C–O); ^1^H NMR (500 MHz, DMSO-d6) δ 11.63 (s, 1H, NH, exchangeable D_2_O), 8.77 (d, *J* = 13.9 Hz, 2H, CH=N, Ar–H), 8.25 (d, *J* = 9.5 Hz, 1H), 8.05 (d, *J* = 9.5 Hz, 1H), 6.94 (s, 1H, CH pyranone ring); 6.02 (s, 1H, CH benzene ring), 5.03 (s, 2H, CH_2_O), 4.22 (dd, *J* = 14.2, 7.1 Hz, 2H, CH_2_), 3.78 (s, 3H, OCH_3_), 2.26 (s, 3H, CH_3_), 1.22 (t, *J* = 7.1 Hz, 3H, CH_3_); m/z: 500 (M^+^) for C_22_H_20_N_4_O_10_ (M.wt 500.42).

##### Ethyl(E)-2-((5-methoxy-2-methyl-4-oxo-6-((2-(2,4,6-trichlorophenyl)hydrazono)methyl)-4H-chromen-7-yl)oxy) acetate (14c):

Red ppt, m.p. 193–5; yield: 89%; IR (cm^−1^) 3286 (NH), 1766 (C=O), 1659 (C=N), 15,602 (C=C), 1187, 1126, 1093, 1045 (O–C–O); ^1^H NMR (500 MHz, DMSO-D6) δ 8.33 (s, 1H, CH=N), 7.60 (s, 2H, Ar–H, NH, exchangeable D_2_O), 7.49 (s, 1H, Ar–H), 7.07 (s, 1H, CH pyranone ring), 6.08 (s, 1H, CH benzene ring), 5.08 (s, 2H, CH_2_O), 4.15–4.14 (dd, *J* = 7.9, 4.3 Hz, 2H, CH_2_), 3.77 (s, 3H, OCH_3_), 2.26 (s, 3H, CH_3_), 1.17 (t, *J* = 7.0 Hz, 3H, CH_3_); ^13^C NMR(125 MHz, DMSO-*d*6) δ 175.55, 168.52, 164.94, 160.04, 159.17, 158.43, 139.20, 134.80, 129.19, 128.67, 128.38, 128.25, 113.56, 112.55, 111.80, 100.02, 98.27, 97.95, 66.14, 63.17, 61.62, 19.82, 14.48.

##### General procedure for the preparation of thiosemicarbazone derivatives 15a–c

A mixture of **8** (0.01 mol), hydrazine hydrate (0.01 mol) and methyl isothiocyanate/or phenyl isothiocyanate or cyclohexyl thiocyanate in absolute ethanol was refluxed for 10–12 h. The reaction was monitored at regular intervals by TLC until the disappearance of **8** (n-hexane/EtOAc, 3:1). The reaction mixture was cooled to room temperature and poured onto ice water. The Precipitate formed was filtered off, washed with water, air-dried and re-crystallized from n-hexane/EtOAc.

##### (E)-2-((5-methoxy-2-methyl-7-(2-(2-(2-methylhydrazine-1-carbonothioyl)hydrazinyl)-2-oxoethoxy)-4-oxo-4H-chromen-6-yl)methylene)-N-methylhydrazine-1-carbothioamide (15a)

Yellow ppt, m.p. 168–70; yield: 65%; IR (cm^−1^) 3356, 3278, 3099 (NH), 1705 (C=O), 1660 (C=N), 1592 (C=C), 1281, 1255 (C=S), 1192, 1152, 1125, 1026 (O–C–O); 1H NMR (500 MHz, DMSO-*d*6) δ 12.29, 9.84, 9.06, 8.32 (s, 5NH, exchangeable D_2_O), 7.92 (s, 1H, CH=N), 6.60, 6.51 (m, 2H), 4.67 (s, 2H, OCH_2_), 3.70 (s, 3H, OCH_3_), 2.32 (m, 9H); ^13^C NMR (125 MHz, DMSO-*d*6) δ 211.23, 210.63, 189.53, 164.68, 161.68, 158.27, 158.21, 156.40, 140.65, 116.98, 116.88, 111.01, 104.62, 100.00, 98.69, 84.61, 67.81, 63.68, 63.02, 19.84, 11.15; m/z: 466 (M^+^) for C_18_H_22_N_6_O_5_S_2_ (M.wt 466.47).

##### (E)-2-((5-methoxy-2-methyl-4-oxo-7-(2-oxo-2-(2-(2-phenylhydrazine-1-carbonothioyl) hydrazin -yl)ethoxy)-4H-chromen-6-yl)methylene)-N-phenylhydrazine-1-carbothioamide (15b)

Yellow canary ppt,m.p.274–6 °C; yield: 90%; IR (cm^−1^) 3336, 3220, 3127 (NH), 1705, 1665 (C=O), 1622 (C=N), 1591 (C=C), 1283, 1224 (C=S), 1196, 1166, 1102, 1061 (O–C–O);1H NMR (500 MHz, DMSO-*d*6) δ 12.71, 9.85, 9.06 (s, 5NH, exchangeable D_2_O), 7.91 (s, 1H, CH=N), 7.25–6.37 (m, 12H, Ar–H), 4.67 (s, 2H, OCH_2_), 3.70 (s, 3H, OCH_3_), 2.30 (s, 3H, CH_3_); ^13^C NMR (125 MHz, DMSO-*d*6) δ 202.17, 190.65, 167.58, 166.84, 161.76, 157.23, 156.15, 139.79, 135.40, 128.57, 114.69, 108.01, 106.20, 104.67, 104.54, 100.03, 99.97, 98.70, 67.79, 63.01, 61.95, 29.97; m/z: 590 (M^+^) for C_28_H_26_N_6_O_5_S_2_ (M.wt 590.69).

##### (E)-N-cyclohexyl-2-((7-(2-(2-(cyclohexylcarbamoyl)hydrazinyl)-2-oxoethoxy)-5-methoxy-2-methyl-4-oxo-4H-chromen-6-yl)methylene)hydrazine-1-carboxamide (15c)

Brown ppt, m.p. 205–7; yield: 75%; IR (cm^−1^) br 3215 (NH), 1727, 1710, 1668 (C=O), 1620 (C=N), 1592 (C=C), 1280, 1227 (C=S), 1197, 1164, 1102, 1062 (O-C-O);1H NMR (500 MHz, DMSO-*d*6) δ 12.97, 10.05, 9.24, 8.04 (s, 4NH exchangeable D_2_O); 7.36 (s, 1H, CH=N); 7.36 (s, 1H, NH exchangeable D_2_O); 6.34 (s, 1H, CH pyrone ring); 6.25 (s, 1H, CH benzene ring); 4.50 (s, 2H, OCH_2_); 3.30 (s, 3H, OCH_3_); 2.30 (m, 5H, CH_3_, CH cyclohexane ring); 1.66–1.09 (m, 20H, CH_2_ cyclohexane ring). m/z: 570 M^+^) C_28_H_38_N_6_O_7_(570.65).

##### General procedure for the preparation of (E)-ethyl 2-((6-((2-carbamothioylhydrazono)methyl)-5-methoxy-2-methyl-4-oxo-4H-chromen-7-yl)oxy)acetate (16)

A mixture of **8** (0.01 mol) and thiosemicarbazide (0.01 mol) in absolute ethanol (50 mL) containing catalytic amounts of glacial acetic acid was heated under reflux for 1 h. The solid obtained was filtered on hot, washed with ethanol, and air dried. The thiosemicarbazone was obtained in an extremely pure state and used without further purification.

Off white ppt, m.p.229–31 °C; yield: 97%; IR (cm^−1^) 3443 (NH_2_), 3228 (NH), 1766 (C=O), 1659 (C=N), 1602, 1592 (C=C), 1245 (C=S), 1187, 1126, 1045, 1020 (O-C-O);1H NMR (500 MHz, DMSO-*d*6) δ 11.41 (s, 1H, NH, exchangeable D_2_O), 8.34 (s, 2H, NH_2,_ exchangeable D_2_O), 7.67 (s, 1H, CH=N), 6.94 (s, 1H, CH pyranone ring), 6.02 (s, 1H, CH benzene ring), 4.97 (s, 2H, OCH_2_), 4.19 (dd, *J* = 14.2, 7.1 Hz, 2H COOCH_2_), 3.72 (s, 3H, OCH_3_), 2.26 (s, 3H, CH_3_) 1.22 (t, *J* = 7.1 Hz, 3H, CH_3_). ^13^C NMR (125 MHz, DMSO-*d*6) δ 178.61, 175.38, 168.45, 164.63, 160.16, 159.90, 159.39, 137.42, 114.78, 112.31, 111.78, 98.37, 66.32, 63.37, 61.55, 56.58, 19.77, 14.57; m/z: 393.1(M^+^) for C_17_H_19_N_3_O_6_S (393.4).

##### General procedure for the preparation of ethyl 2-((5-methoxy-2-methyl-4-oxo-6-((E)-((Z)-(4-oxothiazolidin-2-ylidene)hydrazono)methyl)-4H-chromen-7-yl)oxy)acetate (17)

Toa suspension of the thiosemicarbazone **16** (0.01 mol) and anhydrous potassium carbonate (0.02 mol) in dry acetone (10 mL), bromo ethyl acetate (0.01 mol) was added and the resulting mixture was refluxed under stirring for 12 h. The reaction was monitored at regular intervals by TLC until the disappearance of V13 (n-hexane/EtOAc, 3:1). The reaction mixture was cooled to room temperature and poured onto ice water. The Precipitate formed was filtered off, washed with water, air-dried and re-crystallized from n-hexane/EtOAc.

Off white ppt, m.p.209–11 °C; yield: 83%; IR (cm^−1^) 3180 (NH), 1742, 1679, 1665 (C=O), 1607 (C=N), 1594 (C=C), 1190, 1164, 1130, 1086, 1029, 1011 (O-C-O);1H NMR(500 MHz, DMSO-*d*6) δ 11.93 (s, 1H, NH exchangeable D_2_O), 8.47 (s, 1H, CH=N), 6.97 (s, 1H, CH pyranone ring), 6.03 (s, 1H, CH benzene ring), 5.10 (s, 2H, OCH_2_), 4.15 (q, 2H, COOCH_2_), 3.85–3.80 (m, 5H, CH_2_-thiazolidinone, OCH_3_), 2.27 (s, 3H, CH_3_) 1.20 (t, 3H, CH_3_); ^13^C NMR(125 MHz, DMSO-*d*6) δ 175.50, 168.33, 165.78, 164.96, 160.30, 159.80, 159.57, 142.10, 138.94, 116.56, 113.29, 111.75, 98.33, 66.27, 63.89, 61.47, 24.79, 19.80, 14.53; m/z: 433 (M^+^) for C_19_H_19_N_3_O_7_S (M.wt 433.44).

##### General procedure for the preparation of (E)-ethyl 2-((5-methoxy-2-methyl-4-oxo-6-((2-(4-phenylthiazol-2-yl)hydrazono) methyl)-4H-chromen-7-yl)oxy)acetate (18)

A mixture of thiosemicarbazone **16** (0.01 mol) and phenacyl bromide (0.01 mol) in dry acetone (10 mL) containing anhydrous potassium carbonate (0.02 mol) was refluxed under stirring. The reaction was monitored at regular intervals by TLC until the disappearance of **16** (n-hexane/EtOAc, 3:1). The reaction mixture was cooled to room temperature and poured onto ice water. The Precipitate formed was filtered off, washed with water, air-dried and re-crystallized from n-hexane/EtOAc.

Gray ppt, m.p.136–8 °C; yield: 75%; IR (cm^−1^) 3095 (NH), 1746 (C=O), 1649 (C=N), 1589, 1565 (C=C), 1184, 1134, 1112, 1095, 1057, 1024 (O–C–O);1H NMR (500 MHz, DMSO-*d*6) δ 12.15 (s, 1H, NH exchangeable D_2_O), 8.17 (s, 1H, CH=N), 7.82 (s, 1H, thiazole ring), 7.33–7.26 (m, 5H, Ar–H), 6.94 (s, 1H, CH pyranone ring), 6.02 (s, 1H, CH benzene ring), 5.01 (s, 2H, OCH_2_), 3.82 (s, 3H, OCH_3_), 3.72(q, 2H, COOCH_2_), 2.26 (s, 3H, CH_3_) 1.17 (t, 3H, CH_3_); ^13^CNMR(125 MHz, DMSO-*d*6) δ175.69, 168.93, 168.79, 164.60, 160.26, 159.12, 158.82, 137.48, 135.29, 128.02, 126.06, 115.68, 112.92, 111.82, 104.27, 98.08, 84.40, 83.96, 66.12, 63.30, 52.61, 19.77, 14.53. m/z: 493.1 (M^+^) for C_25_H_23_N_3_O_6_S (493.5).

##### General procedure for the preparation ofethyl 2-((6-(5-acetamido-3-acetyl-2,3-dihydro-1,3,4-thiadiazol-2-yl)-5-methoxy-2-methyl-4-oxo-4H-chromen-7-yl)oxy)acetate (19)

The thiosemicarbazone intermediate **16** (1.0 eq.) was added to a stirring solution of acetic anhydride (5.0 eq.) and the reaction mixture was stirred for 10 h at 90 C. The mixture was poured into ice water. The organic layer was extracted using ethyl acetate, and dried over anhydrous sodium sulphate. The solvent was evaporated under vacuum and the solid obtained was re-crystallized from n-hexane/EtOAc.

Brown ppt, m.p 288–90 °C; yield: 62%; %; IR (cm^−1^) 3286 (NH);1733, 1650 (3C=O); 1600 (C=N), 1455 (C=C), 11,903, 1165, 1110, 1175, 1039 (O–C–O); ^1^H NMR (500 MHz, DMSO-*d*6) δ 9.64 (s, 1H, NH exchangeable D_2_O), 7.16 (s, 1H, CH thiadiazole ring), 6.94 (s, 1H, CH pyranone ring), 6.02 (s, 1H, CH benzene ring), 4.02 (s, 2H, OCH_2_), 4.14 (q, 2H, COOCH_2_), 3.81 (s, 3H, OCH_3_), 2.26 (s, 3H, COCH_3_); 1.88 (s, 3H, 1CH_3_); 1.19 (m, 6H, 2CH_3_); m/z: 477.1 (M^+^) for C_21_H_23_N_3_O_8_S (477.5).

##### General procedure for the preparation of(Z)-ethyl 2-((6-((2,4-dioxothiazolidin-5-ylidene)methyl)-5-methoxy-2-methyl-4-oxo-4H-chromen-7-yl)oxy)acetate (20)

To a solution of **8** (1 mmol) and thiazolidine-2,4-dione (0.13 g, 1 mmol)in absolute ethanol (10 mL), a few drops of piperidine was added and the mixture was heated under reflux for 12 h. The progress of the reaction was monitored by TLC (n-hexane/EtOAc, 3:1). The mixture was cooled to room temperature and quenched with ice water. The Precipitate formed was filtered off, washed with water, air-dried and re-crystallized from methanol.

Brown powder, m.p.129–31 °C; yield: 62%; IR (cm^−1^) 3366 (NH), 1742, 1711, 1644 (C=O), 1576, (C=C), 1186, 1116, 1060, 1016 (O–C–O); ^1^H NMR(500 MHz, DMSO-*d*6) δ 9.91 (s, 1H, NH exchangeable D_2_O), 8.07 (s, 1H, CH=C), 7.13 (s, 1H, CH pyranone ring), 6.38 (s, 1H, CH benzene ring), 5.09 (s, 2H, OCH_2_), 4.34 (m, 2H, COOCH_2_), 4.19 (s, 3H, OCH_3_), 2.39 (s, 3H, CH_3_) 1.23 (t, *J* = 7.8 Hz, 3H, CH_3_); ^13^C NMR (125 MHz, DMSO-*d*6) δ175.61, 168.18, 166.62, 162.36, 158.29, 127.85, 125.43, 110.36, 102.85, 97.88, 67.64, 66.21, 61.99, 61.56, 23.07, 20.36, 14.65, 14.55; m/z: 419 (M^+^) for C_19_H_17_NO_8_S (M.wt 419.40).

## Biological assays

### Chemicals

Doxorubicin, DMEM, DMEM-F12, penicillin/streptomycin, trypsin solution and fetal bovine serum were purchased from Lonza, Spain. 3-(4,5-dimethylthiazolyl-2)-2,5-diphenyltetra-zolium bromide (MTT) was obtained from Sigma-Aldrich, St. Louis, MO, USA. Triton X-100 was from Pierce Biotechnology Inc., Rockford, IL, USA.

### Cell lines and cell cultures

HCT-116 (colon), HepG2 (liver), and MCF-7 (breast) cancer cell lines were obtained from Karolinska Institute, Stockholm, Sweden. Cancer cell lines were grown in Dulbecco’s modified Eagle’s medium (DMEM) supplemented with 10% fetal bovine serum (FBS). Cells were cultured in DMEM media supplied with 10% fetal bovine serum, and 100 U/mL penicillin/streptomycin. The cells were maintained at 37 °C in 5% CO_2_.

### Cell proliferation and viability

The cytotoxic activity against the different cancer cell lines was determined according to the method of Thabrew et al.^37^, with slight modifications. The cells were seeded into a 96-well plate at a concentration of 20,000 cells/well for HCT-116 and PC3 cell lines and 10,000 cells/well for HepG2, A549 and MCF-7 cell lines. After 24 h, the media was aspirated and replaced with serum-free media containing the tested compounds (100 μM). The cells were treated for 48 h in triplicates. For the positive and negative controls, doxorubicin (100 μM) and dimethyl sulfoxide (DMSO) (0.5%) were used, respectively. Cell viability in response to treatments was calculated using the MTT [3-(4, 5-dimethylthiazol-2-yl)-2,5-diphenyltetrazolium bromide] assay^38^. The percentage of cytotoxicity was calculated using the following equation:

$$\% {\text{Cytotoxicity}} = \left[ {{1} - \left( {{\text{AVx}}/{\text{AVNC}}} \right)} \right] \times {1}00$$

where AVx denotes the average absorbance of the sample well and AVNC denotes the average absorbance of the negative control well measured at 595 nm with reference at 690 nm.

### Determination of IC50 values

Active compounds possessing cytotoxicity ≥ 60% on different cancer cell lines were selected for dose–response studies at different concentrations. The final tested concentrations were 100, 50, 25, 12.5, and 6.25 μM in triplicates. The IC50 values were calculated using the concentration–response curve fit to the non-linear regression model using Graph Pad Prism® v6.0 (GraphPad Software Inc., San Diego, CA, USA).

### DNA fragmentation assay

#### DNA gel electrophoresis laddering assay

Apoptotic DNA fragmentation was qualitatively analyzed by detecting the laddering pattern of nuclear DNA as described according to^39^. A 100-bp DNA ladder (Invitrogen, USA) was included as a molecular size marker and DNA fragments were visualized and photographed by exposing the gels to ultraviolet transillumination.

#### Diphenylamine reaction procedure

Cancer cell line samples (MCF7 and HCT116) were used to determine the quantitative profile of the DNA fragmentation. MCF7 and HCT116 samples were collected immediately after sacrificing the animals. The cell line samples were lysed in 0.5 mL of lysis buffer containing, 10 mMtris-HCl (pH 8), 1 mM EDTA, 0.2% triton X-100, centrifuged at 10 000 rpm (Eppendorf) for 20 min at 4 °C. The pellets were re-suspended in 0.5 mL of lysis buffer. To the pellets (P) and the supernatants (S), 0.5 mL of 25% tri-chloroacetic acid (TCA) was added and incubated at 4 °C for 24 h. The samples were then centrifuged for 20 min at 10,000 rpm (Eppendorf) at 4 °C and the pellets were suspended in 80 mL of 5% TCA, followed by incubation at 83 °C for 20 min. Subsequently, to each sample, 160 mL of Diphenyl Amine (DPA) solution [150 mg DPA in 10 mL glacial acetic acid, 150 mL of sulfuric acid and 50 mL acetaldehyde (16 mg:mL)] was added and incubated at room temperature for 24 h^40^. The proportion of fragmented DNA was calculated from absorbance reading at 600 nm wavelengths using the formula:

$$\begin{aligned} & \% {\text{ Fragmented DNA}} = {{{\text{[OD}}\left( {\text{S}} \right)} \mathord{\left/ {\vphantom {{{\text{[OD}}\left( {\text{S}} \right)} {\left[ {{\text{OD}}\left( {\text{S}} \right) + {\text{OD}}\left( {\text{P}} \right)} \right]}}} \right. \kern-0pt} {\left[ {{\text{OD}}\left( {\text{S}} \right) + {\text{OD}}\left( {\text{P}} \right)} \right]}} \times {1}00 \\ & \quad \left( {{\text{OD}}:{\text{ optical density}},{\text{ S}}:{\text{ supernatants}},{\text{ P}}:{\text{ pellets}}} \right) \\ \end{aligned}$$

### Statistical analysis

All data were analyzed using the General Linear Models (GLM) procedure of the Statistical Analysis System (1982)^41^ followed by the Scheffé-test to assess significant differences between groups. The values are expressed as mean ± SEM. All statements of significance were based on a probability of *P* < 0.05.

#### Isolation of total RNA and RT-PCR

All of the extractions were conducted on the ice with ice-cold reagents. Total RNA from the different cell lines were isolated using Trizol (Invitrogen; Life Technologies, USA) A high-capacity cDNA reverse transcription kit was used to produce complementary DNA (cDNA, Applied Biosystems, USA)^42^. Table 8 shows the primers that were used in these tests. The relative gene expression method (i.e., ΔΔCT) was used to analyze the real-time PCR data, as explained in Applied Biosystem User Bulletin No. 2. Each sample and gene were normalized using the β-actin gene.

**Table 8:** Primer’s sequence used for *RT-qPCR.*

Genes	F	R	GenBank (accession no)
P53 (Tumor protein)	GGCCCACTTCACCGTACTAA	GTGGTTTCAAGGCCAGATGT	AH002919.2
CDK4 (cyclin-dependent kinases)	TGTATGGGGCCGTAGGAACC	GCAGGGATACATCTCGAGGC	NM_000075.4
BAX (Bcl-2 Associated X-protein)	CTGGATCCAAGACCAGGGTG	CCTTTCCCCTTCCCCCATTC	NR_027882.2
Bcl-2 (B-cell lymphoma 2)	CCTTTGTGGAACTGTACGGC	CCGGCCAACAACATGGAAAG	NM_000633.3
β –actin	TTGCCGACAGGATGCAGAA	GCCGATCCACACGGAGTACT	HQ154074.1

*P53* tumor protein, *CDK4* cyclin-dependent kinases, *BAX* Bcl-2 associated X-protein, *Bcl2* B-cell lymphoma 2.

#### Cell cycle arrest and apoptosis detection

Cellular DNA content was analyzed using a flow cytometer. After 24 h of the culture of HepG2 and treated with naringin and NDN at concentrations of 188.26 and 214.57 µg/mL, respectively, DNA was stained with propidium iodide (PI) (ab139418 PI Flow Cytometry Kit/BD, Sigma, St. Louis, MO) to determine cell cycle distribution. The cell distribution percentage in the G0/G1, G2/M, and S phases was measured, and the cell cycle profile was calculated based on the results. Cells were stained with annexin V–FITC and PI labelling as directed by the manufacturer (BioVision, Annexin V–FITC Apoptosis Detection Kit, USA, Catalog #: K101-25) to determine apoptotic cell populations. A flow cytometry analysis of cell cycle distribution (FACS) was performed using a FACS.

### Molecular docking

The molecular docking was performed using AutoDock Vina in PyRx software version 8^43^. The three-dimensional structure of CDK4-Cyclin D3 bound to abemaciclib was acquired from the RCSB protein data bank in the PDB format utilizing **7SJ3** code (https://www.rcsb.org/structure/7SJ3 access on 12 September 2023). The unwanted co-crystallized ligand and water molecules were removed and the enzyme was prepared using the QuickPrep tool module in the MOE program, saved as pdb and converted to PDBQT format by Autodock vina tools. Our docking protocol was validated by re-docking the co-crystallized ligand,abemaciclib (N-{5-[(4-ethylpiperazin-1-yl)methyl]pyridin-2-yl}-5-fluoro-4-[4-fluoro-2-methyl-1-(propan-2-yl)-1H-benzimidazol-6-yl]pyrimidin-2-amine). The chemical structure of the selected molecules was constructed with the *ChemDraw* ultra 10.0, saved as an SDF file then minimized by applying the MMFF94 force field and converted to a pdbqt file using OpenBable tools involved in Pyrx software. AutoDock Tools was employed to set the size and the centre of the grid box. The size of the CDK4 active site was set at 22.55 × 13.25 × 22.98 Å coordinates in x, y, and z dimensions and centred to x = 13.14, y = -38.18, z = 10.07. PyRx software presents the 9 most suitable docking poses of the ligand–protein complex after the docking is completed and subsequently ranked according to the binding energy. We have selected the first docking pose which is the most suitable pose where the ligands have the lowest binding energy, zero Å root-mean-square deviation (RMSD)and strongly interact with the protein's catalytic cavity and visualized them using BIOVIA Discovery Studio Visualizer to have a great insight into ligand binding position in the protein cavity.

### Drug likeliness and ADMET prediction

The drug likeliness and some ADMET endpoints of the selected active compounds; **6a**, **6b**, **11**, **14b**, **14c**, **17** and **19** were predicted utilizing the SwissADME (http://www.swissadme.ch/index.php) and *admetSAR 2.0* (http://lmmd.ecust.edu.cn/admetsar2) respectively. The physicochemical descriptors calculated, including molecular MW, LogP, HBA, HBD, nRB, MR, TPSA and were analyzed taking into account Lipinski’s rule of five and Veber filter rule.

### Supplementary Information

Supplementary Figures.

## Data Availability

All data generated or analyzed during this study are included in this published article [and its supplementary information files].
