# Supplementary material for: Synthesis, bioactivity assessment, molecular docking and ADMET studies of new chromone congeners exhibiting potent anticancer activity
Source: Sci Rep. 2024 Apr 26;14:9636. doi: 10.1038/s41598-024-59606-2 (PMC11053072; doi:10.1038/s41598-024-59606-2)

Figure s1: ^{1}HNMR (DMSO) spectrum for compound **2**

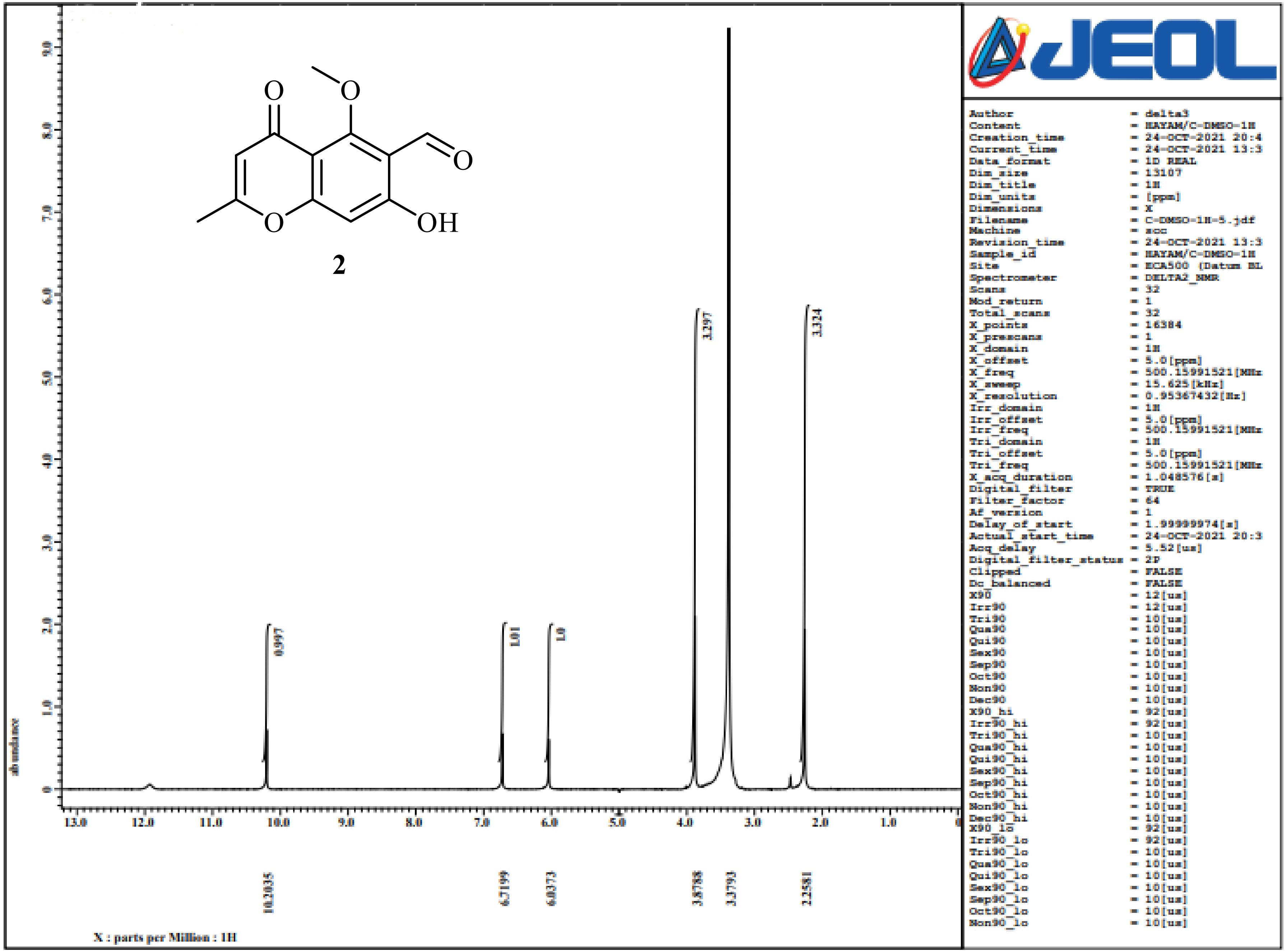

Figure s2: IR spectrum for compound **3**

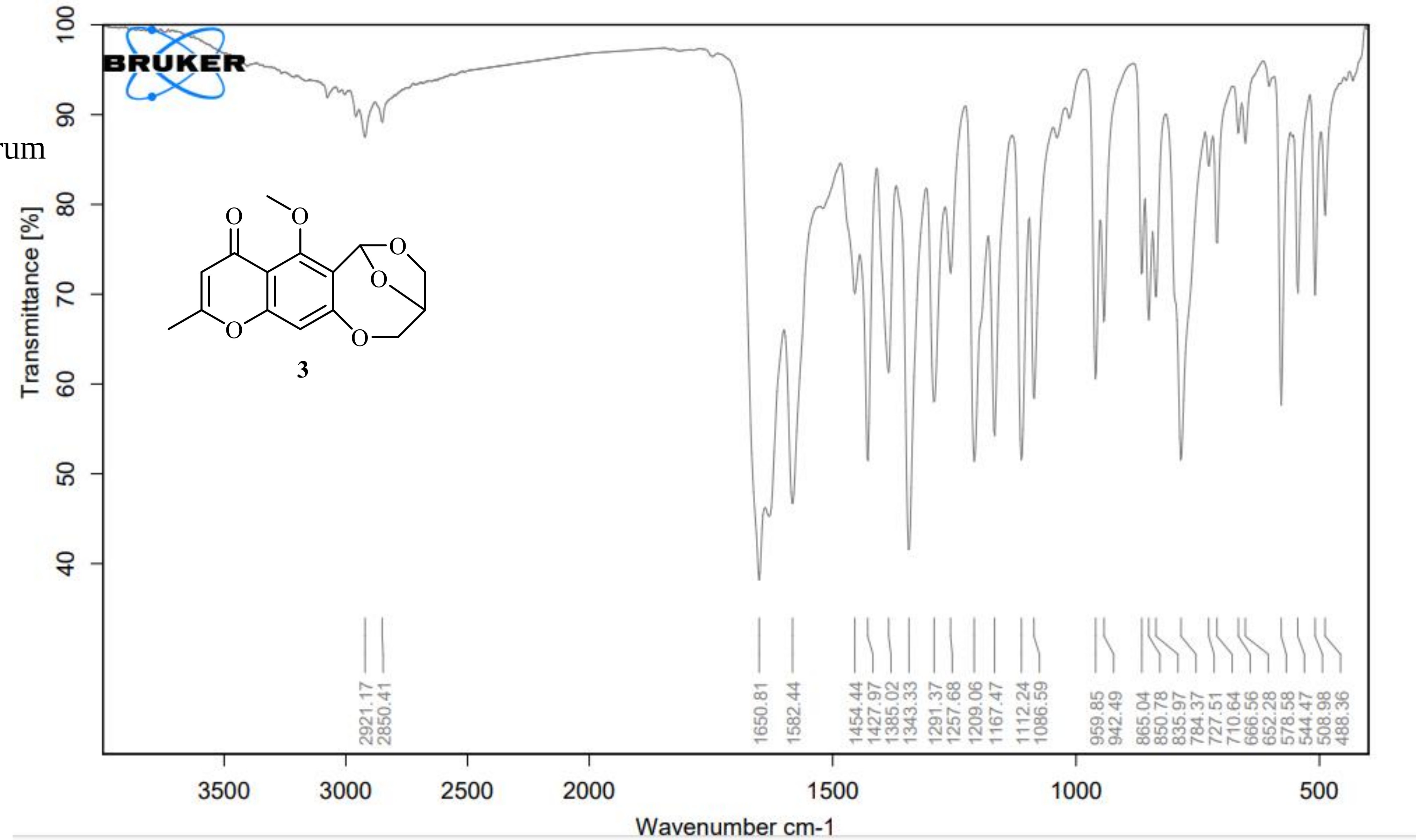

Figure s3: ^{1}HNMR (DMSO) spectrum for compound **3**

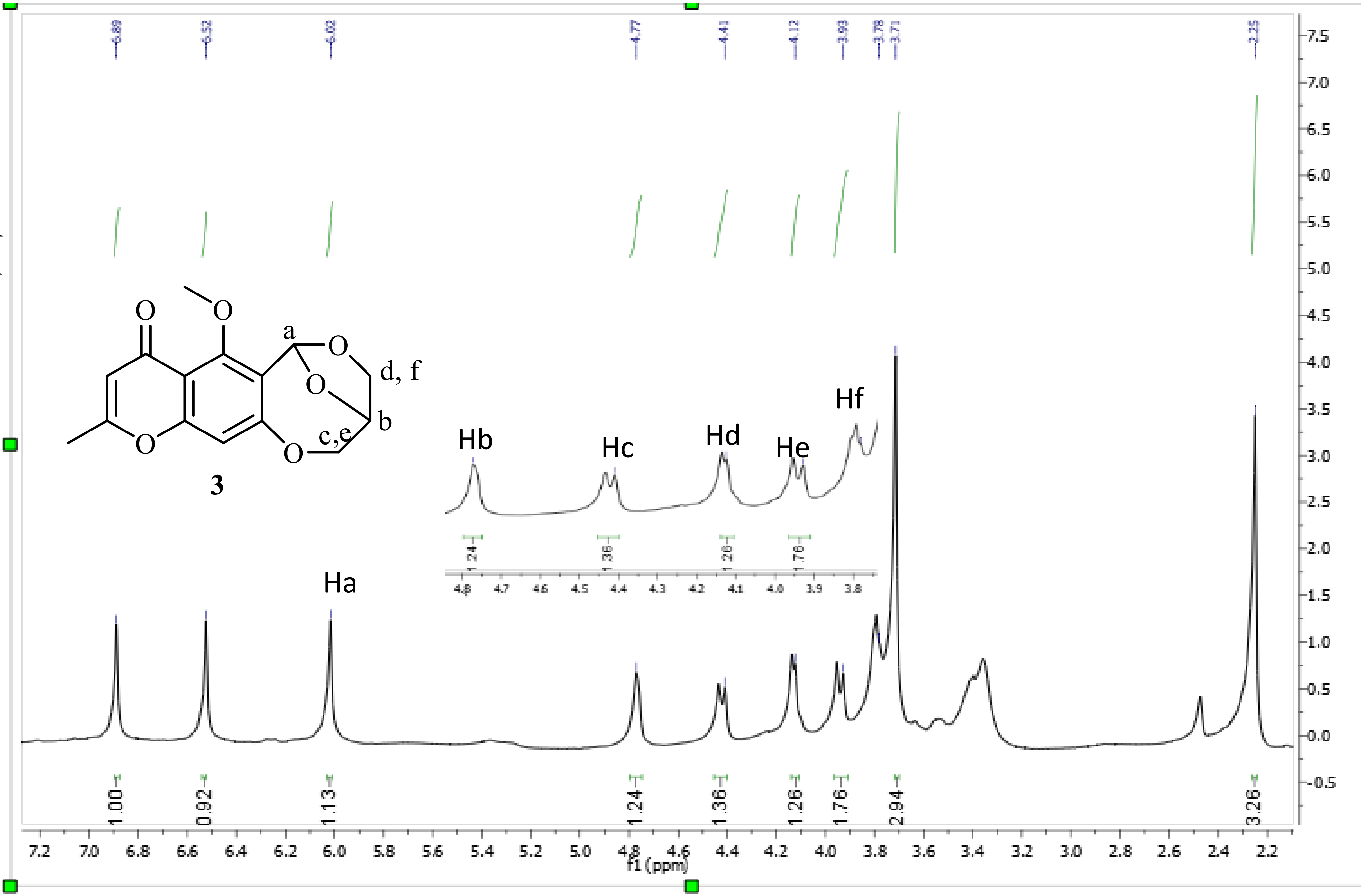

Figure s4: Mass spectrum for compound **3**

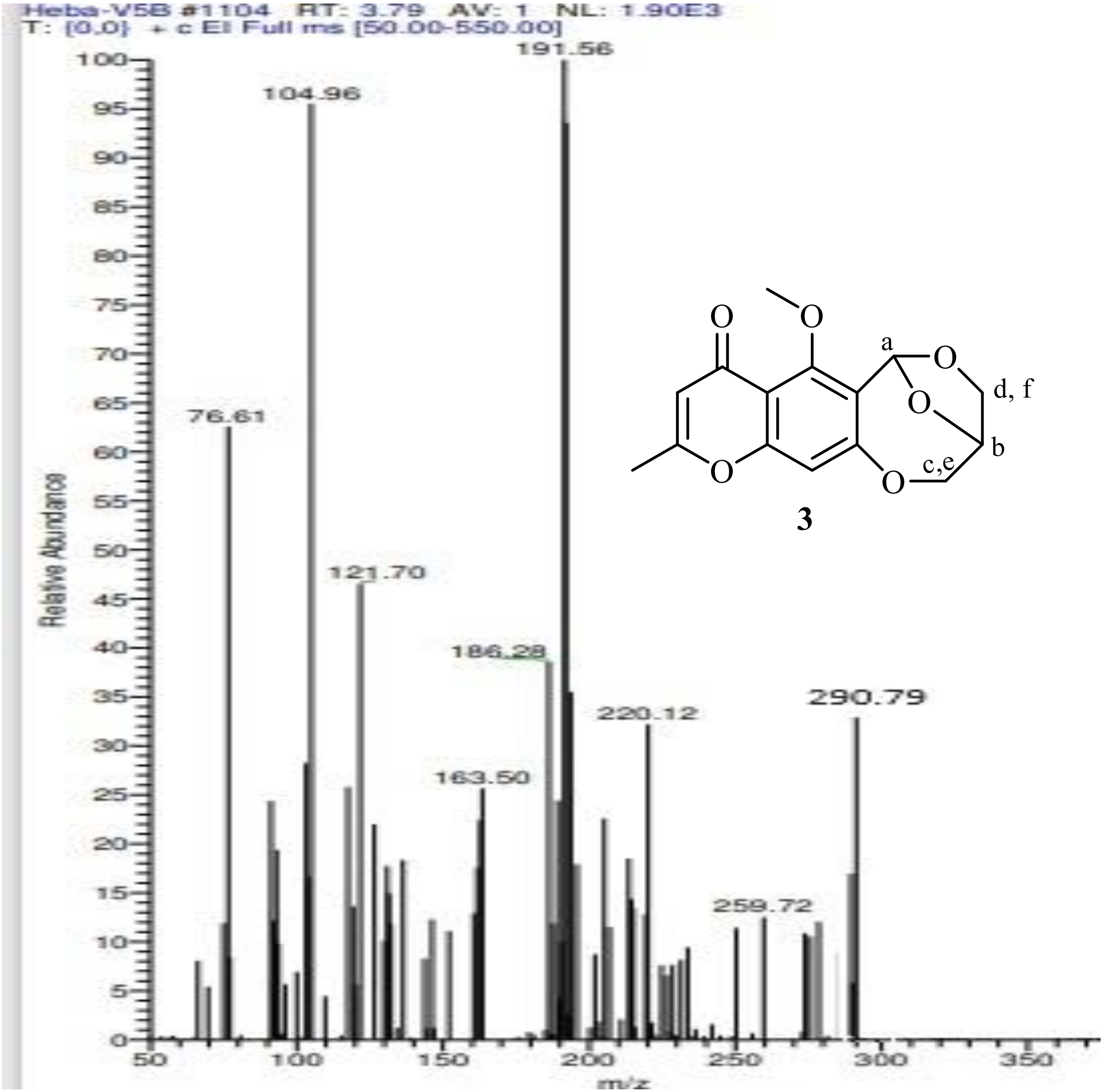

3

Figure s5: ^{1}HNMR (DMSO) spectrum for compound **4**

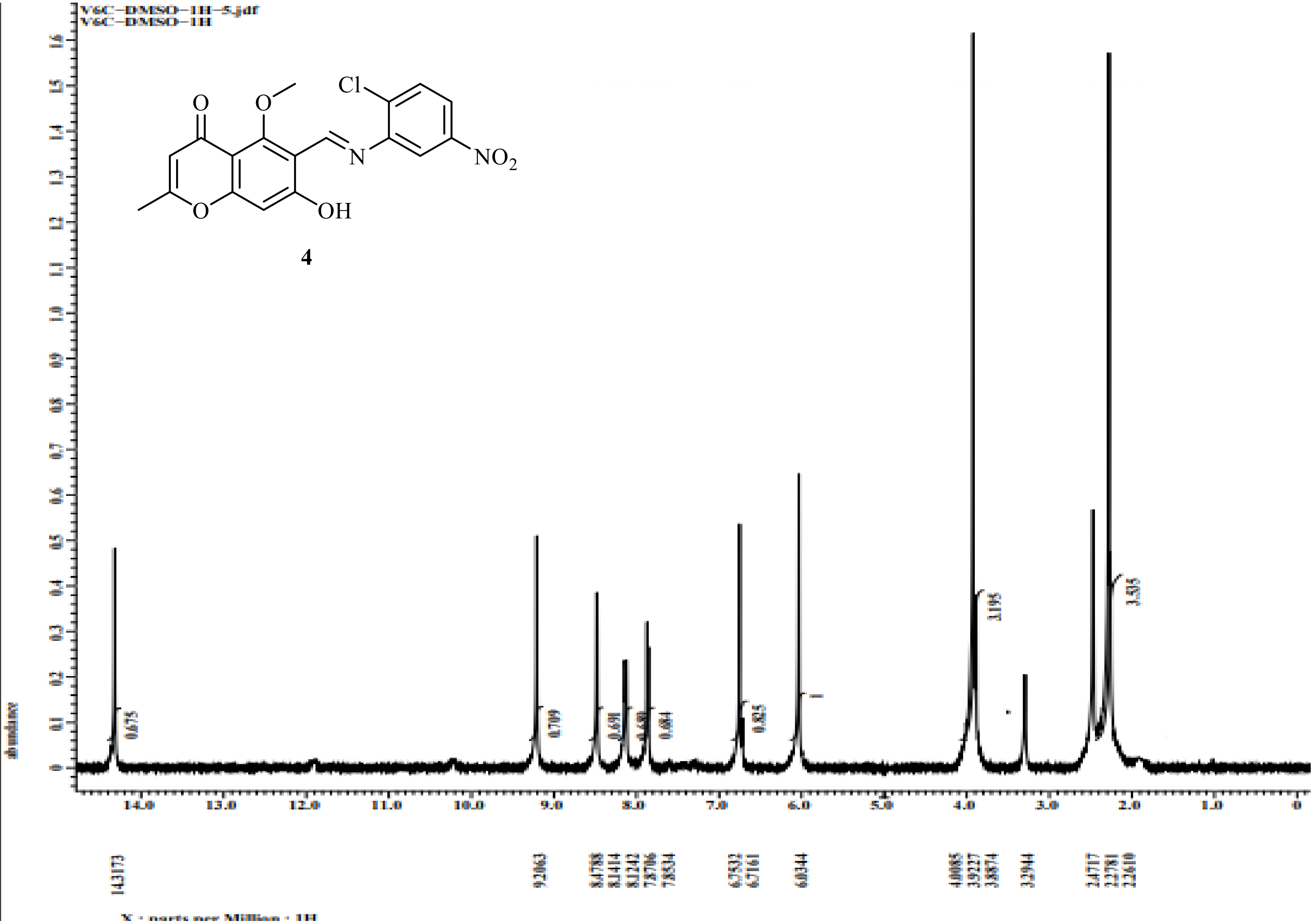

Figure s6: IR spectrum for compound **5**

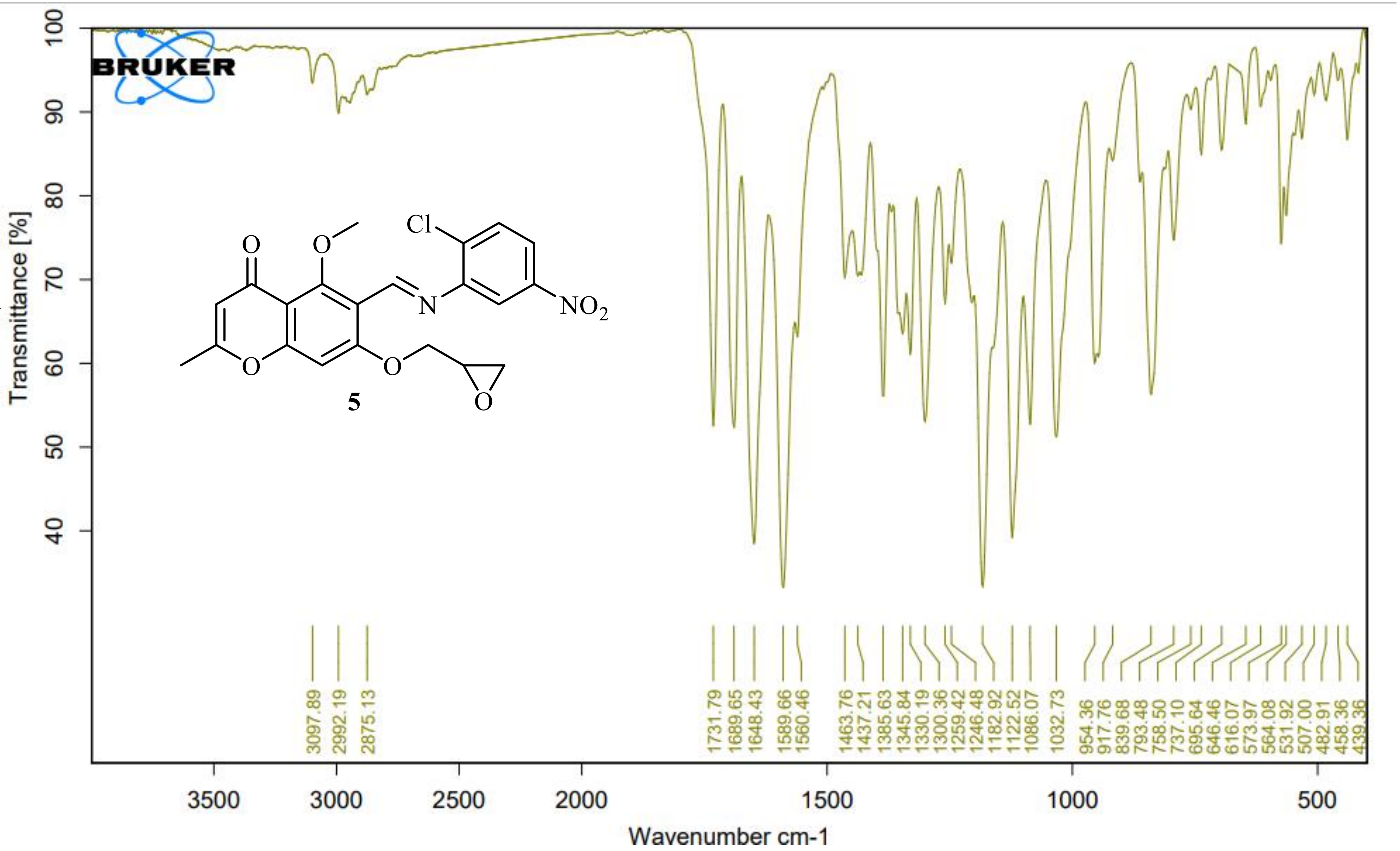

Figure s7a: ^{1}HNMR (DMSO) spectrum for compound **5**

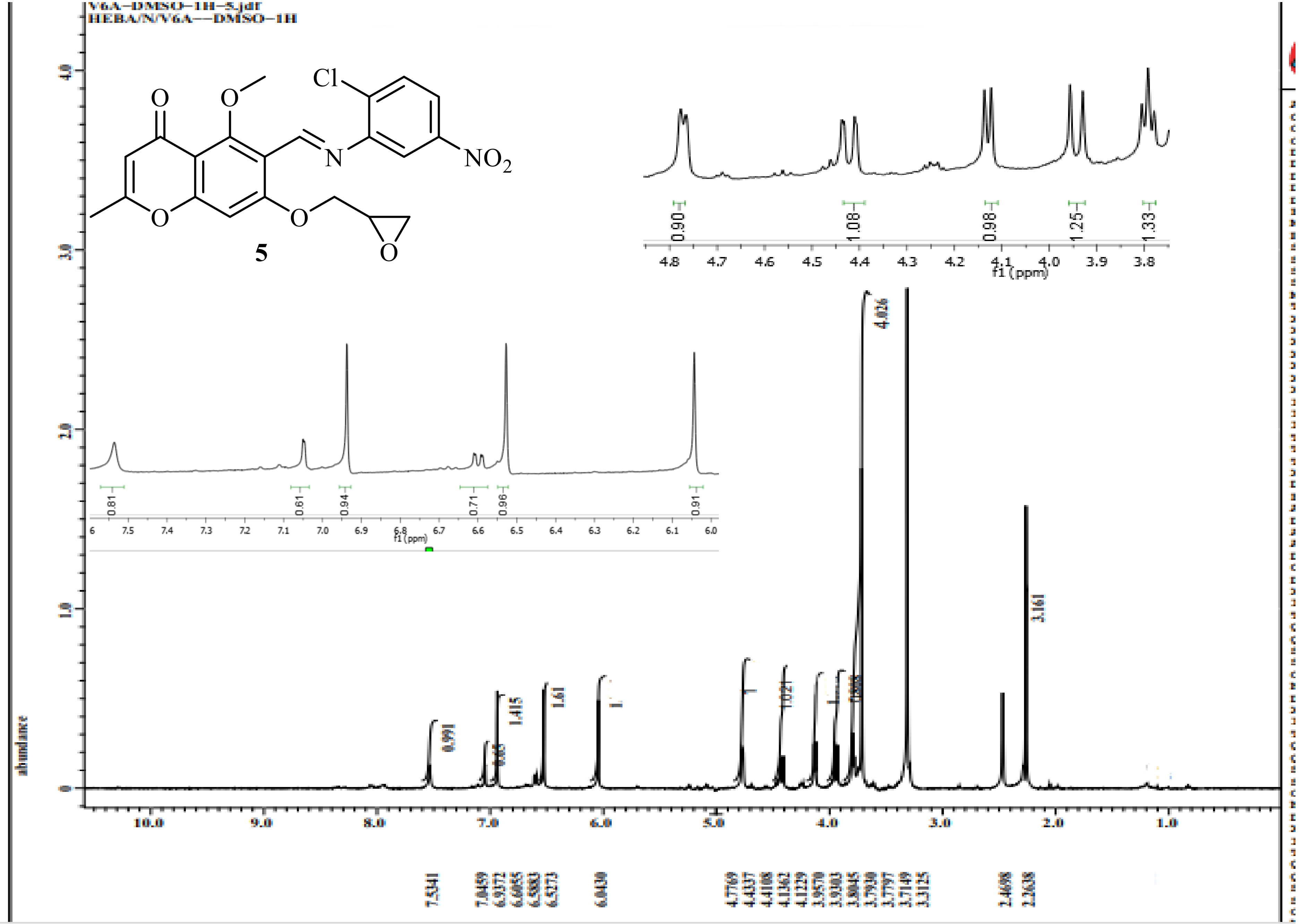

Figure s7b: ^{1}HNMR (DMSO) spectrum for compound **5**

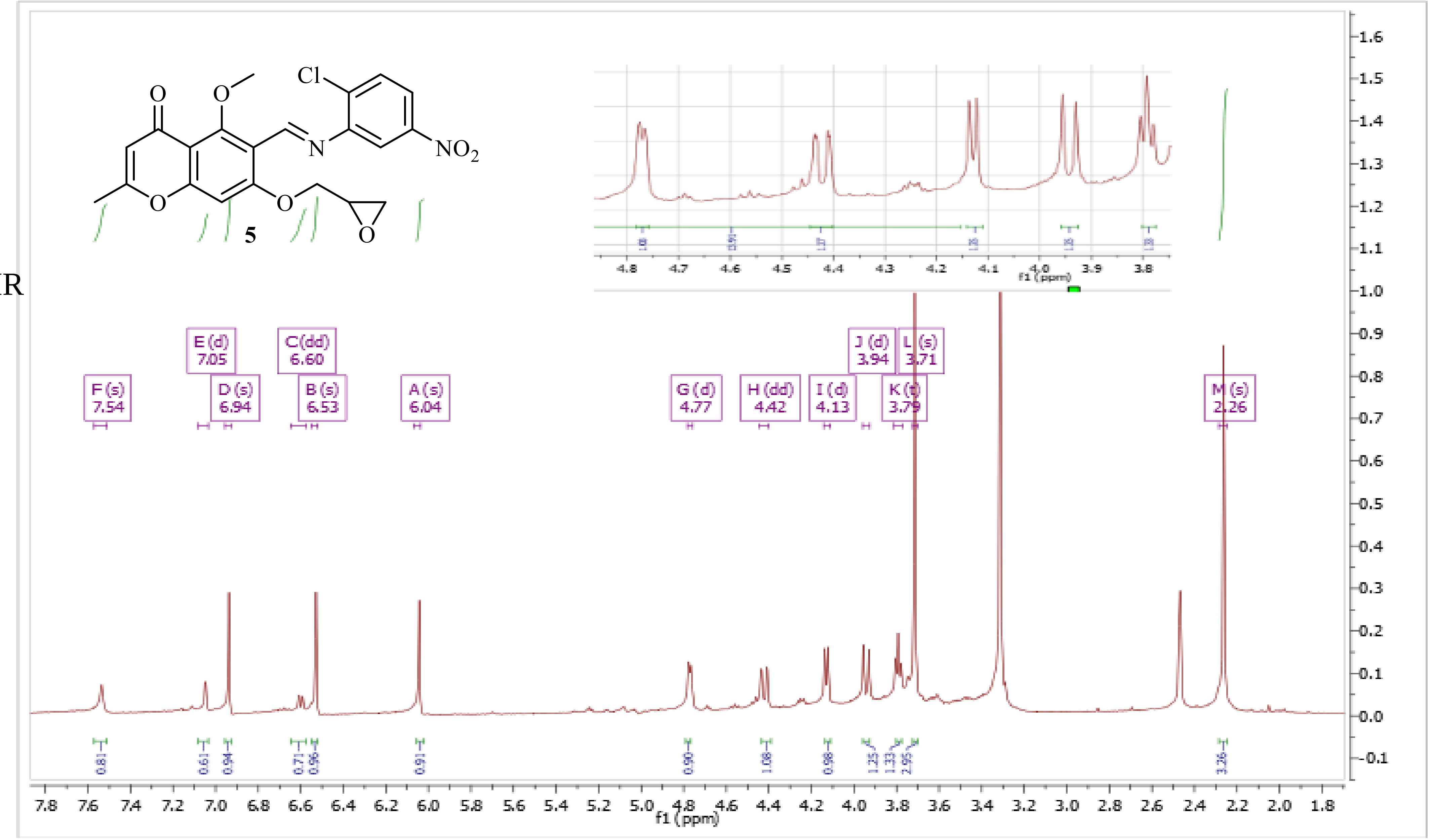

Figure s8: ^{13}CNMR (DMSO) spectrum for compound **5**

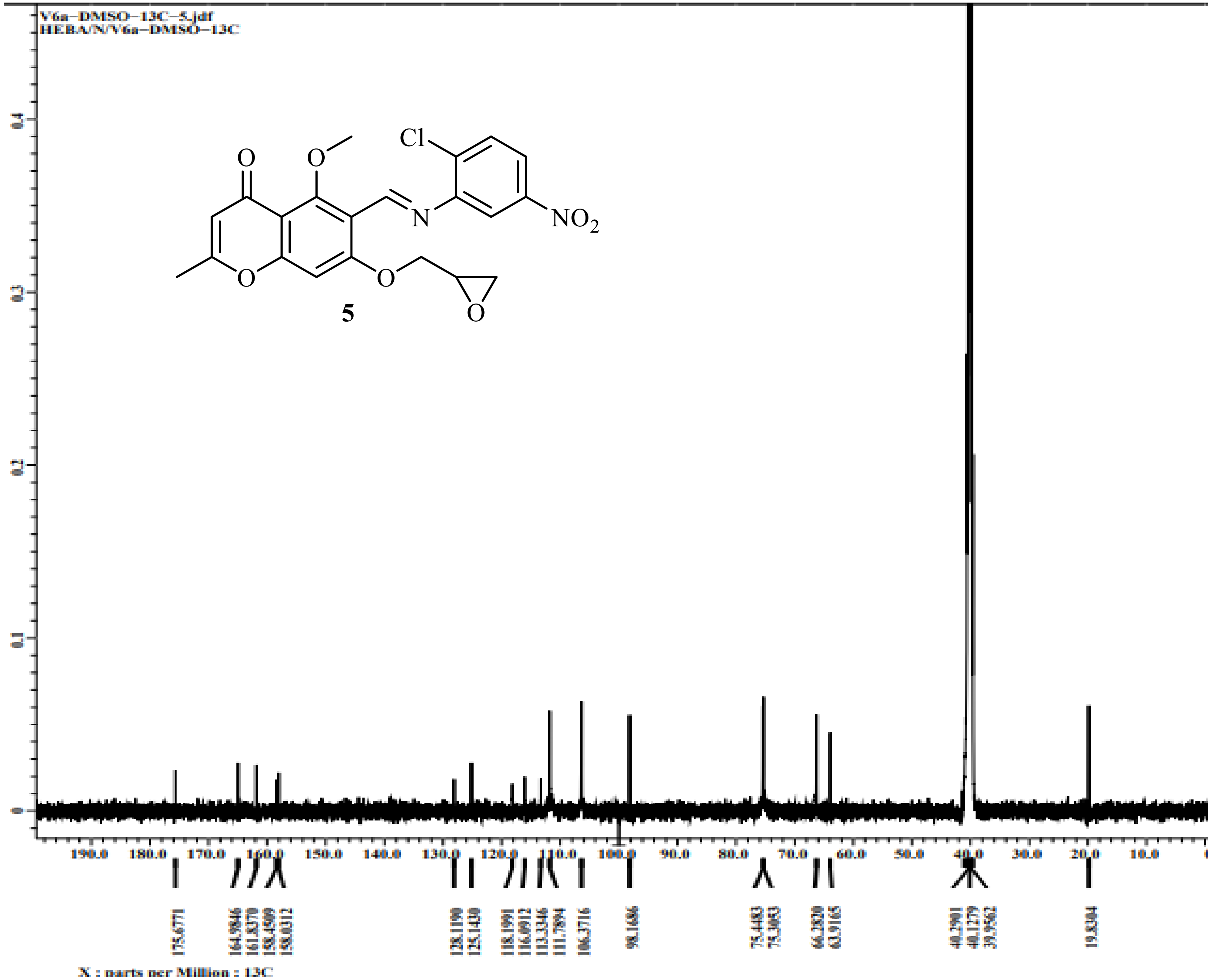

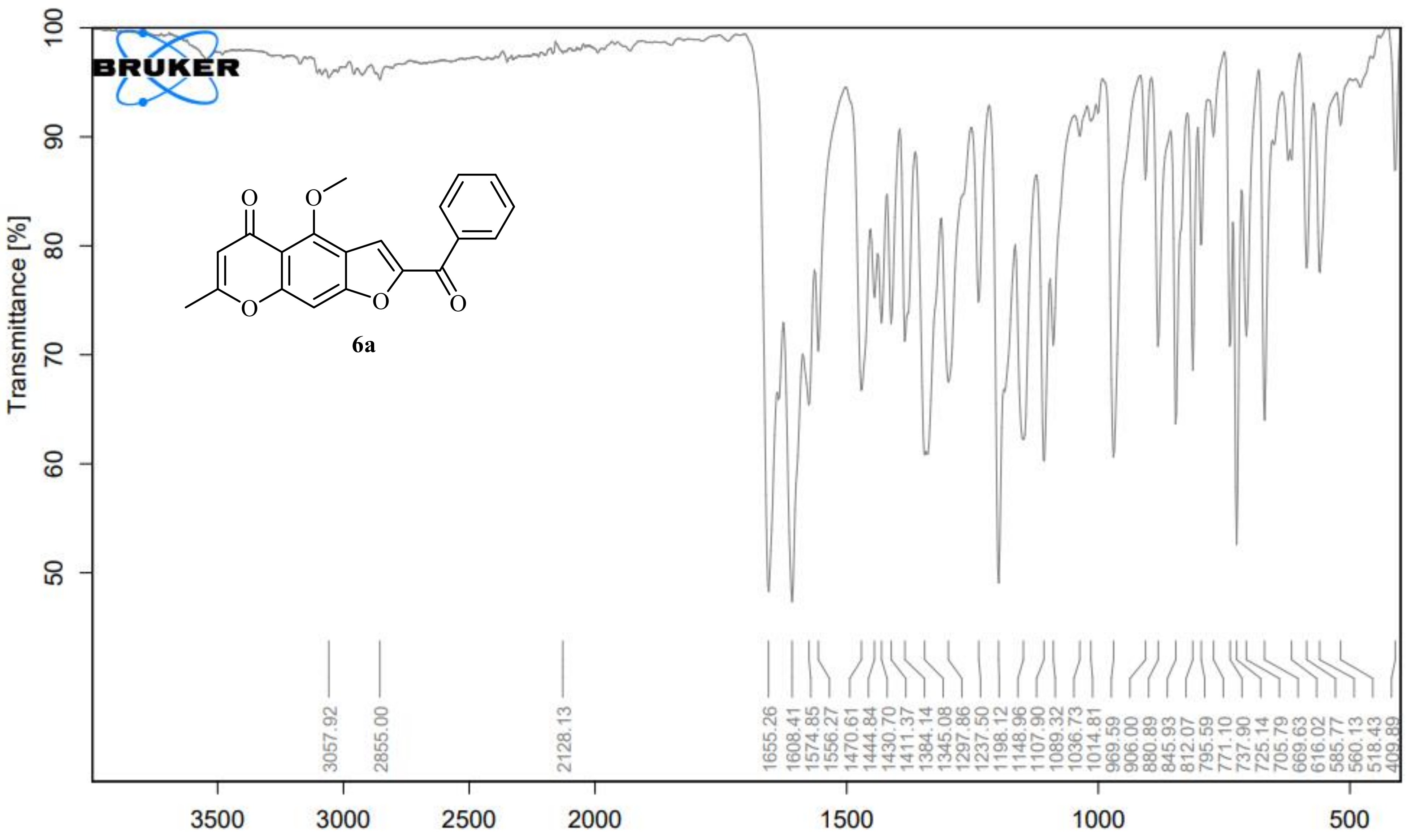

Figure s9: IR spectrum for compound **6a**

Figure s10: ^{1}HNMR (DMSO) spectrum for compound **6a**

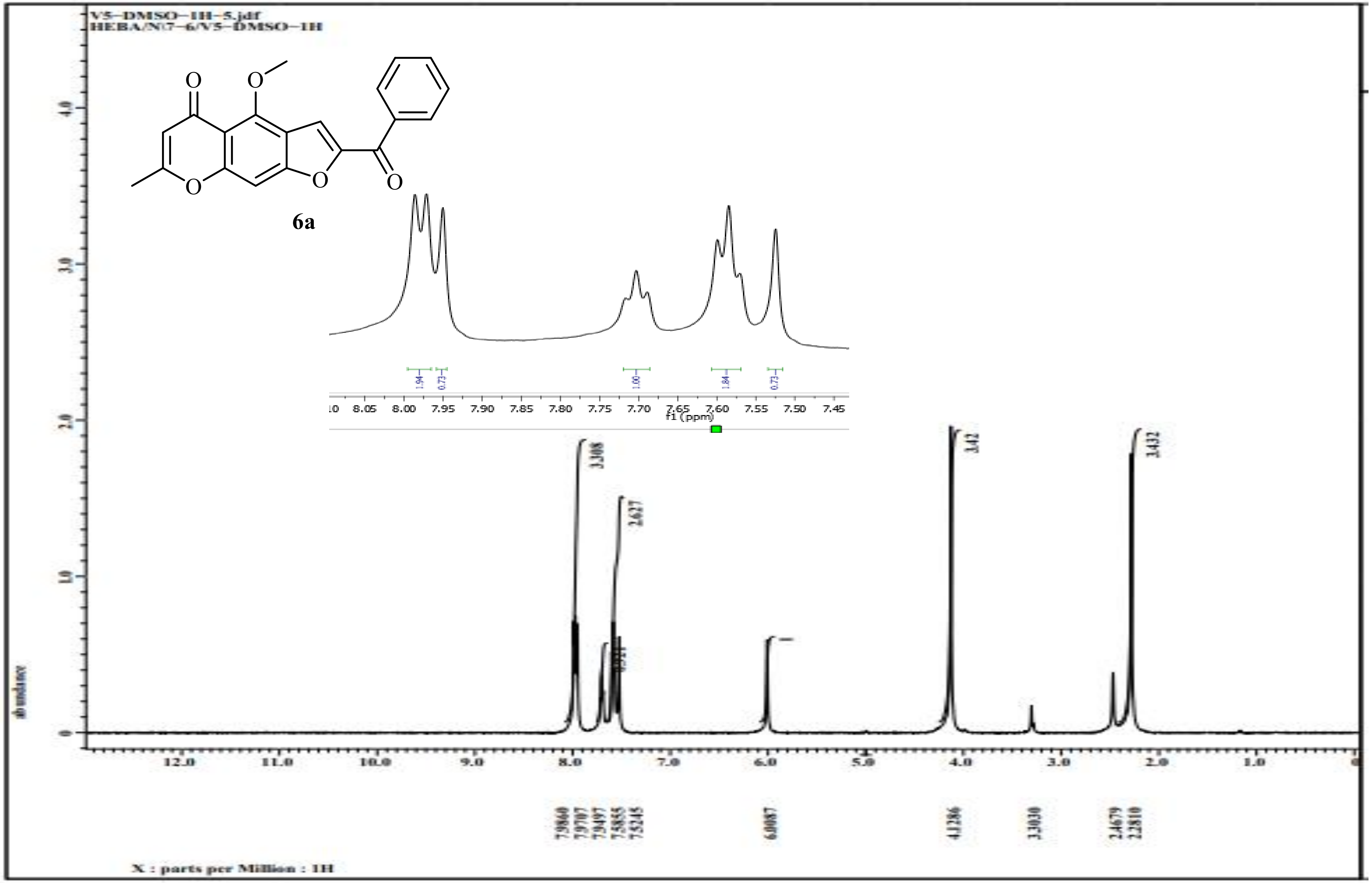

Figure s11: ^{13}CNMR (DMSO) spectrum for compound **6a**

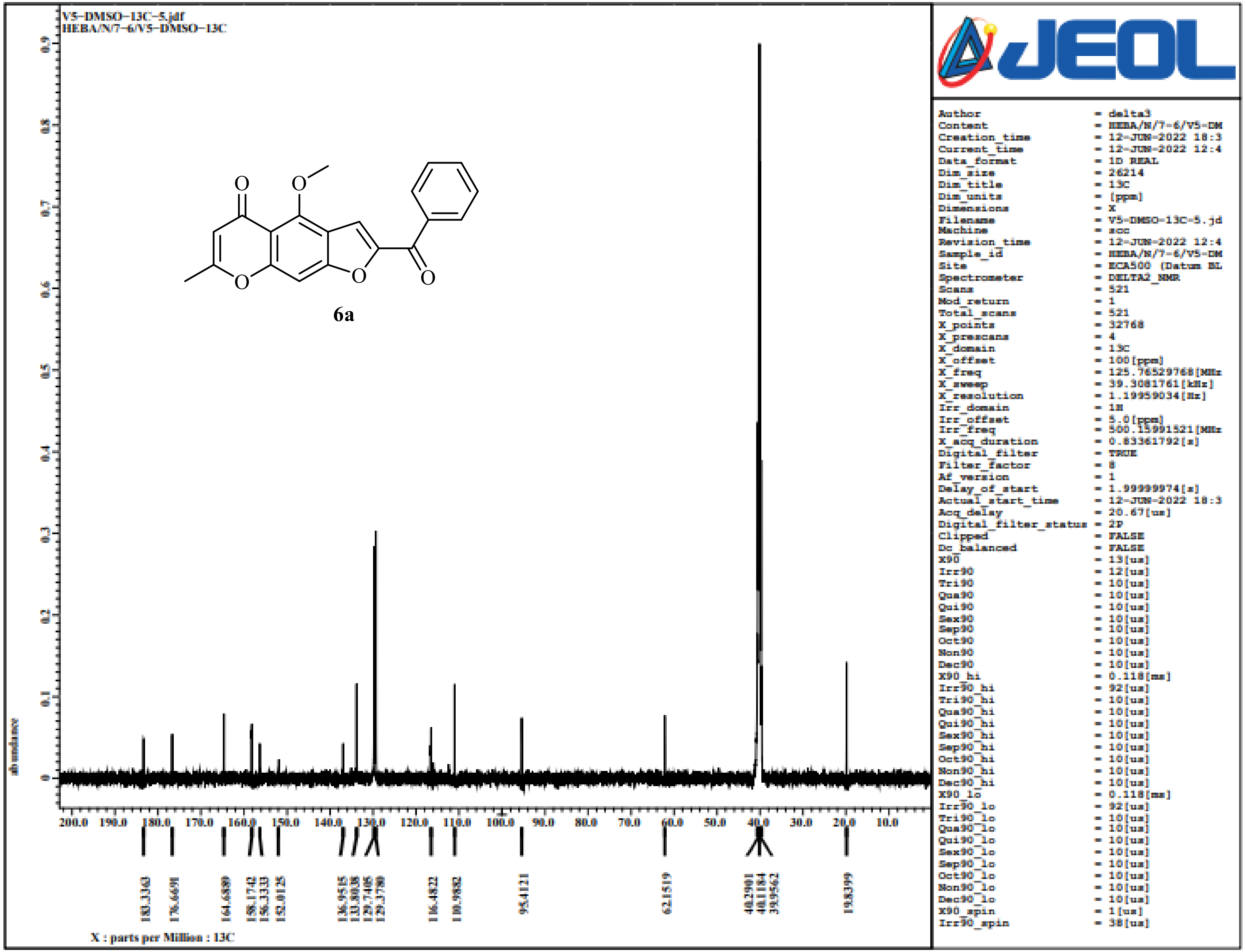

Figure s12: Mass spectrum for compound **6a**

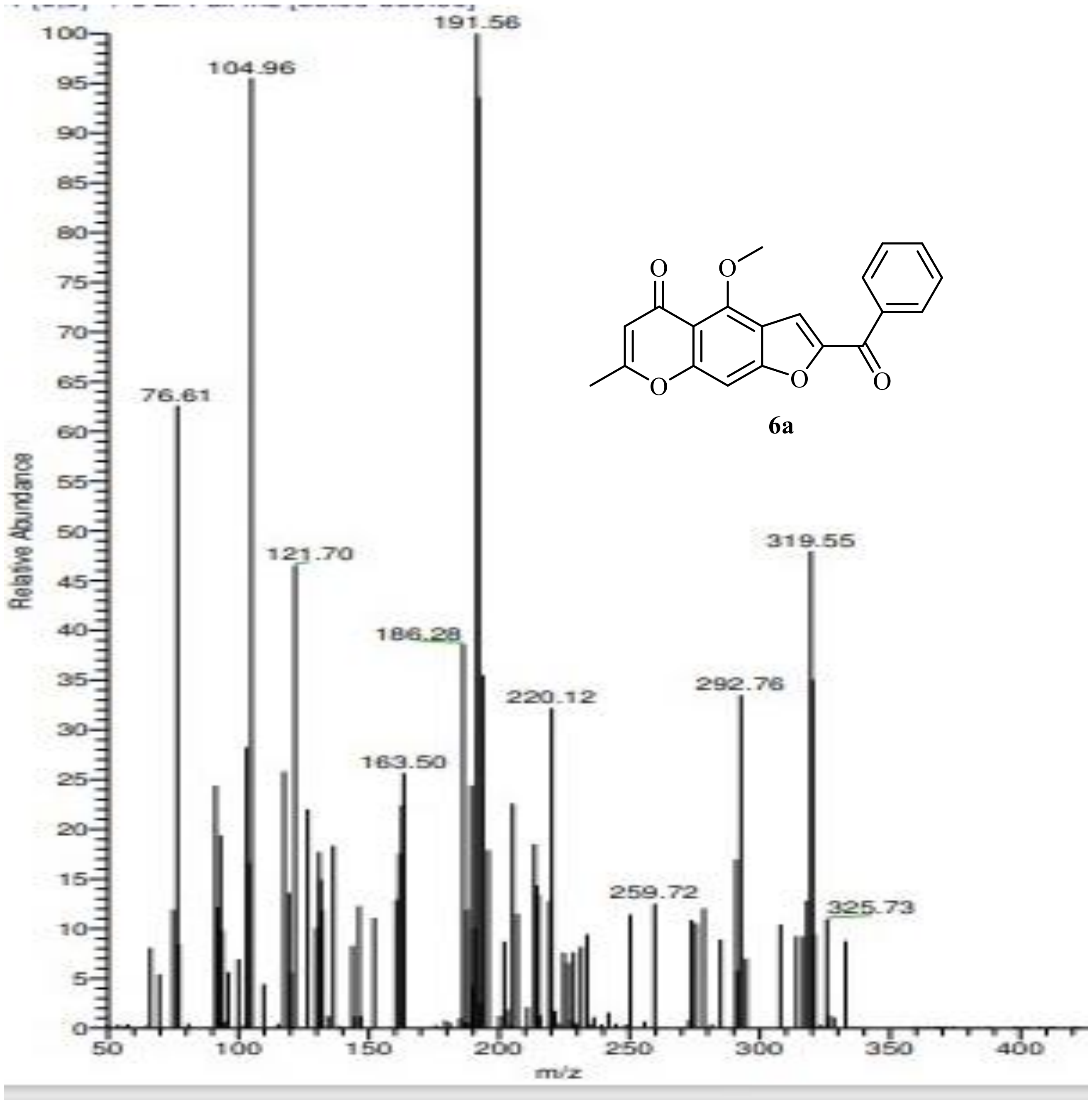

Figure s13: IR spectrum for compound **6b**

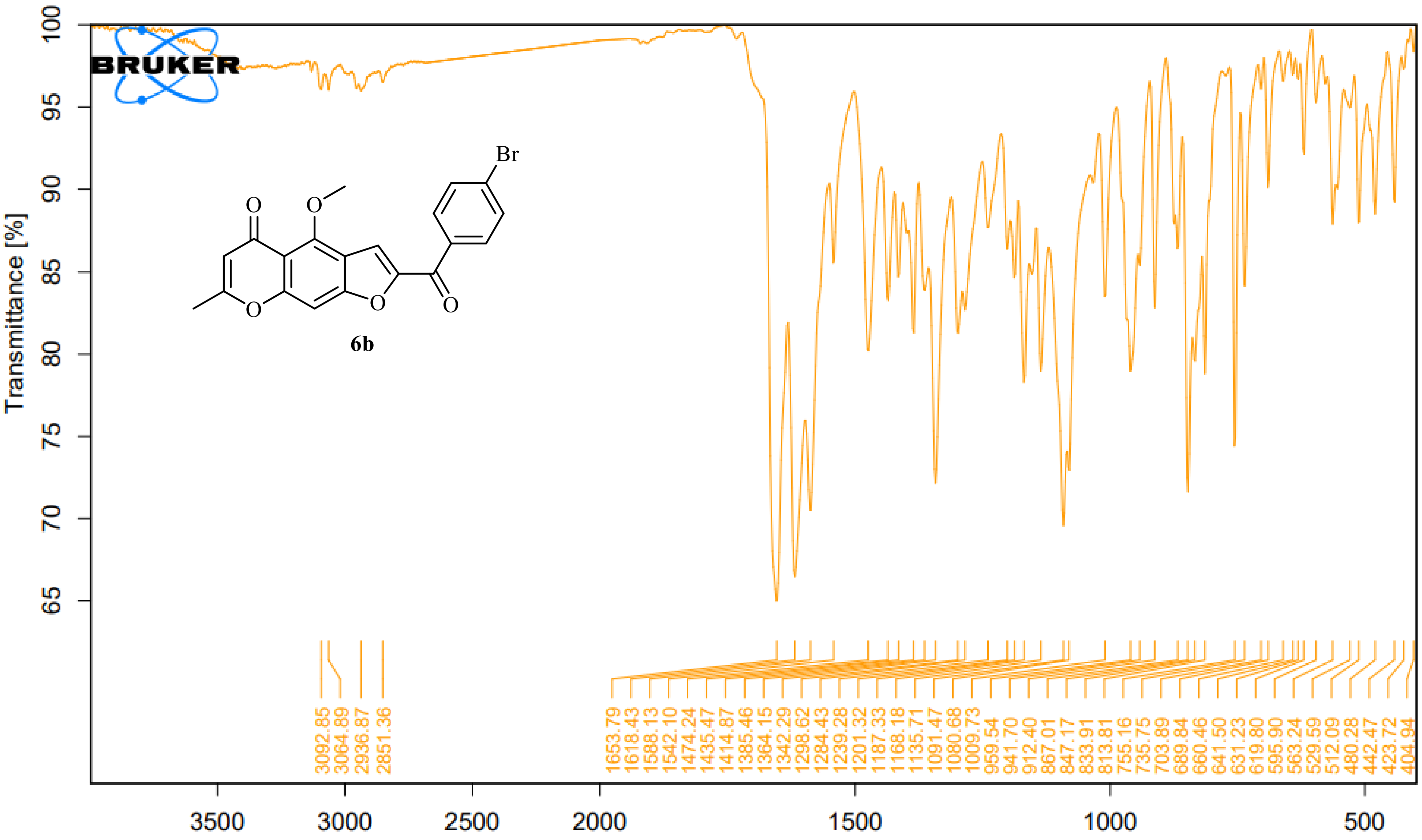

Figure s14: ^{1}HNMR (DMSO) spectrum for compound **6b**

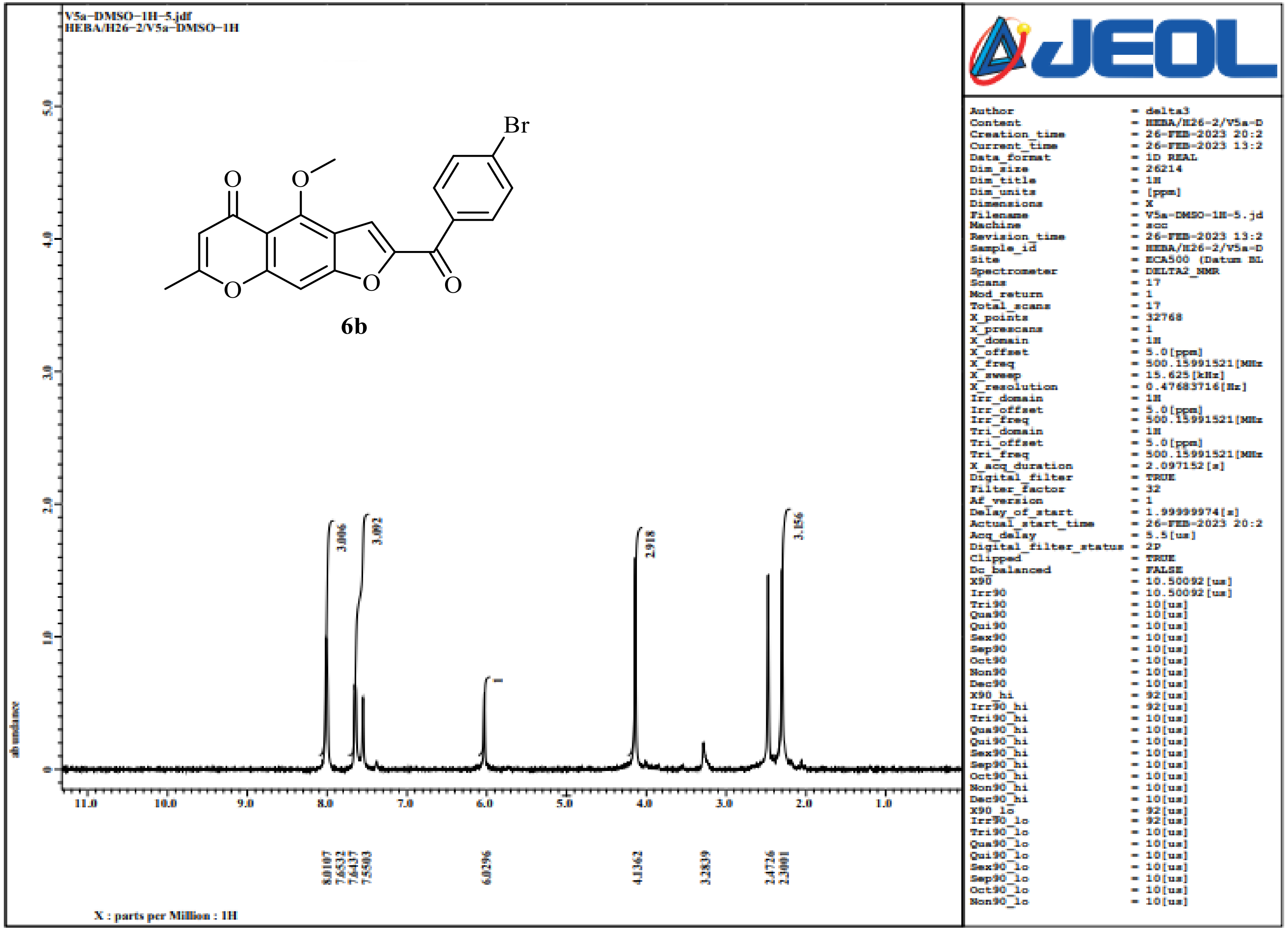

Figure s15: ^{13}CNMR (DMSO) spectrum for compound **6b**

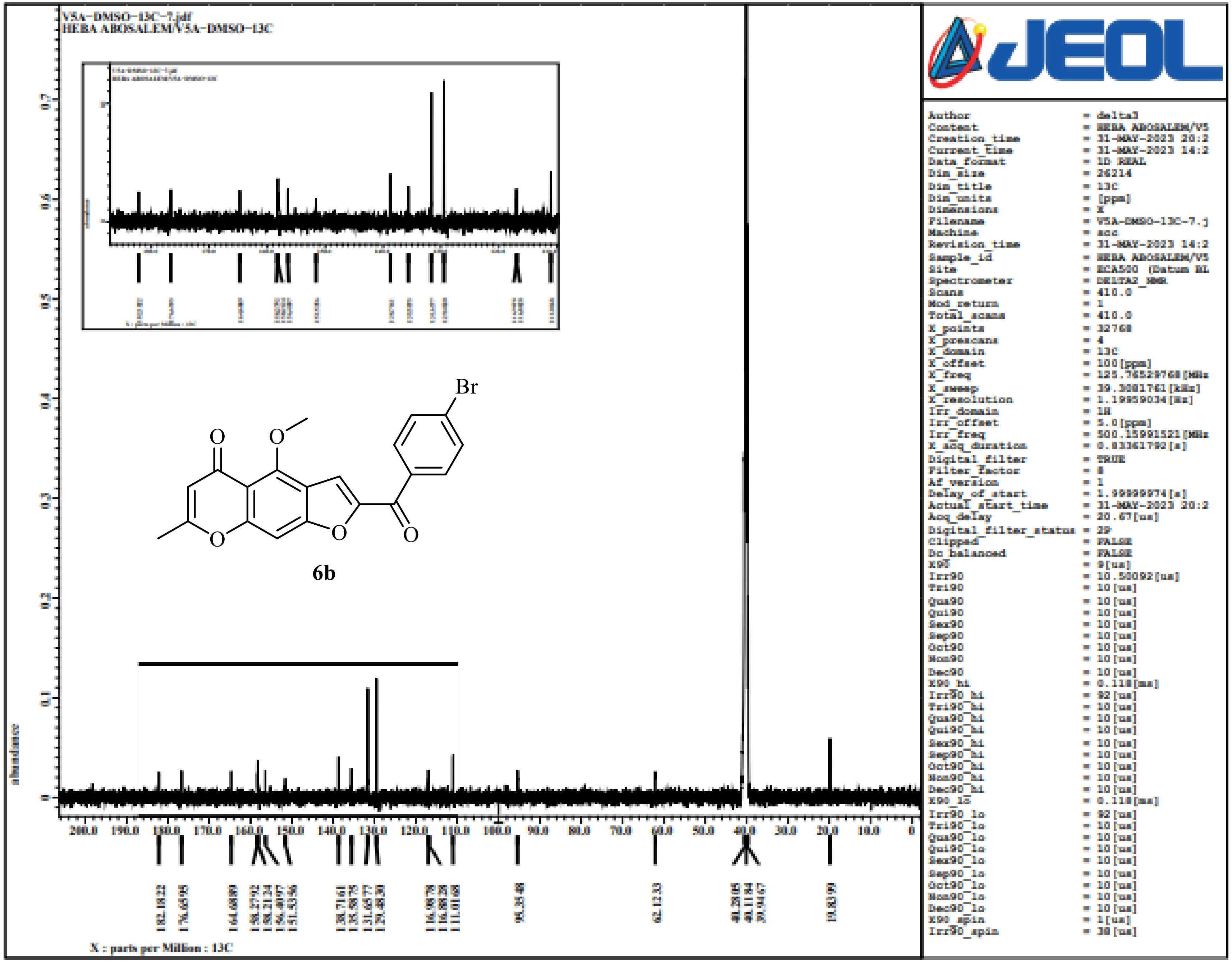

Figure s16: IR spectrum for compound **7**

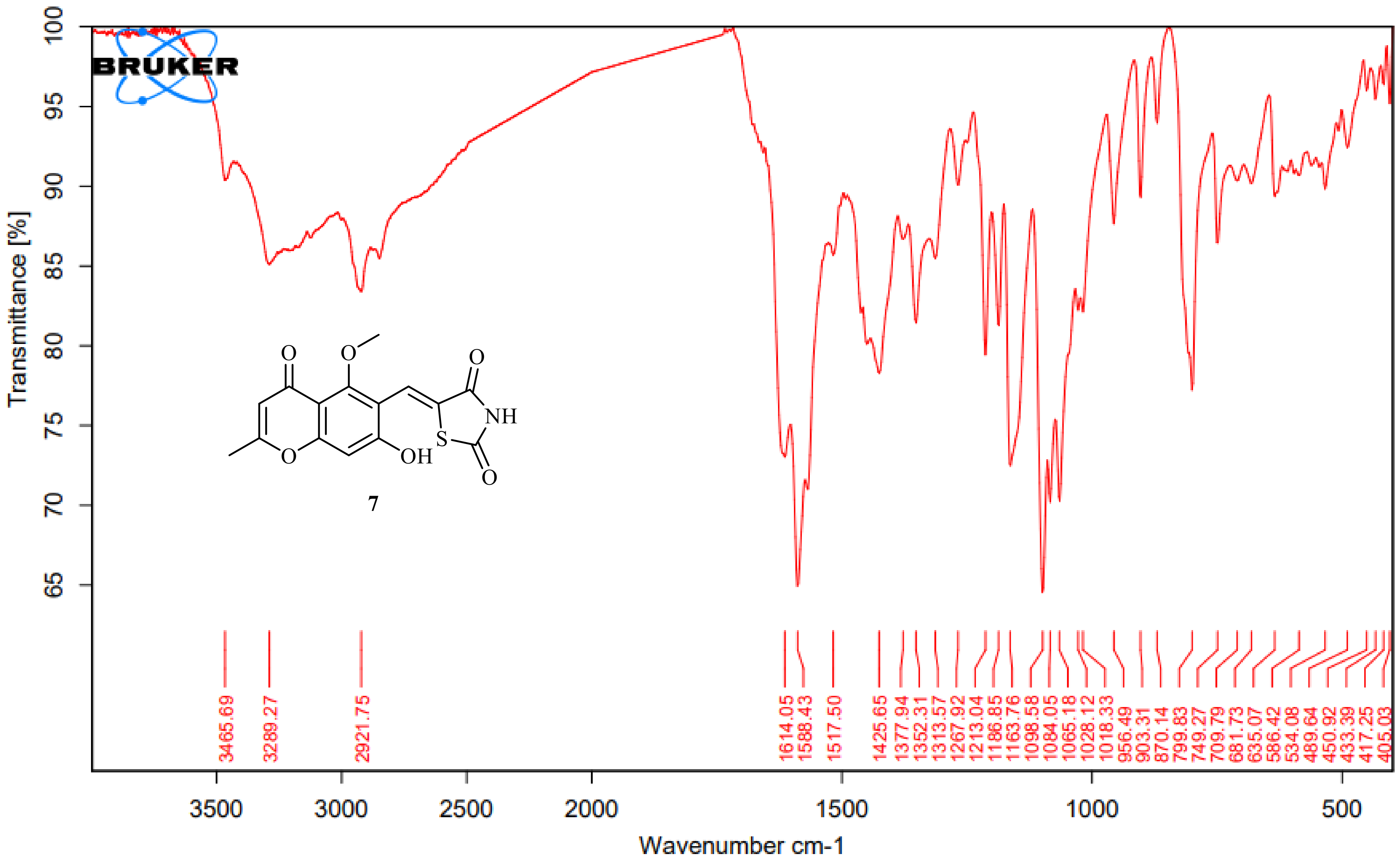

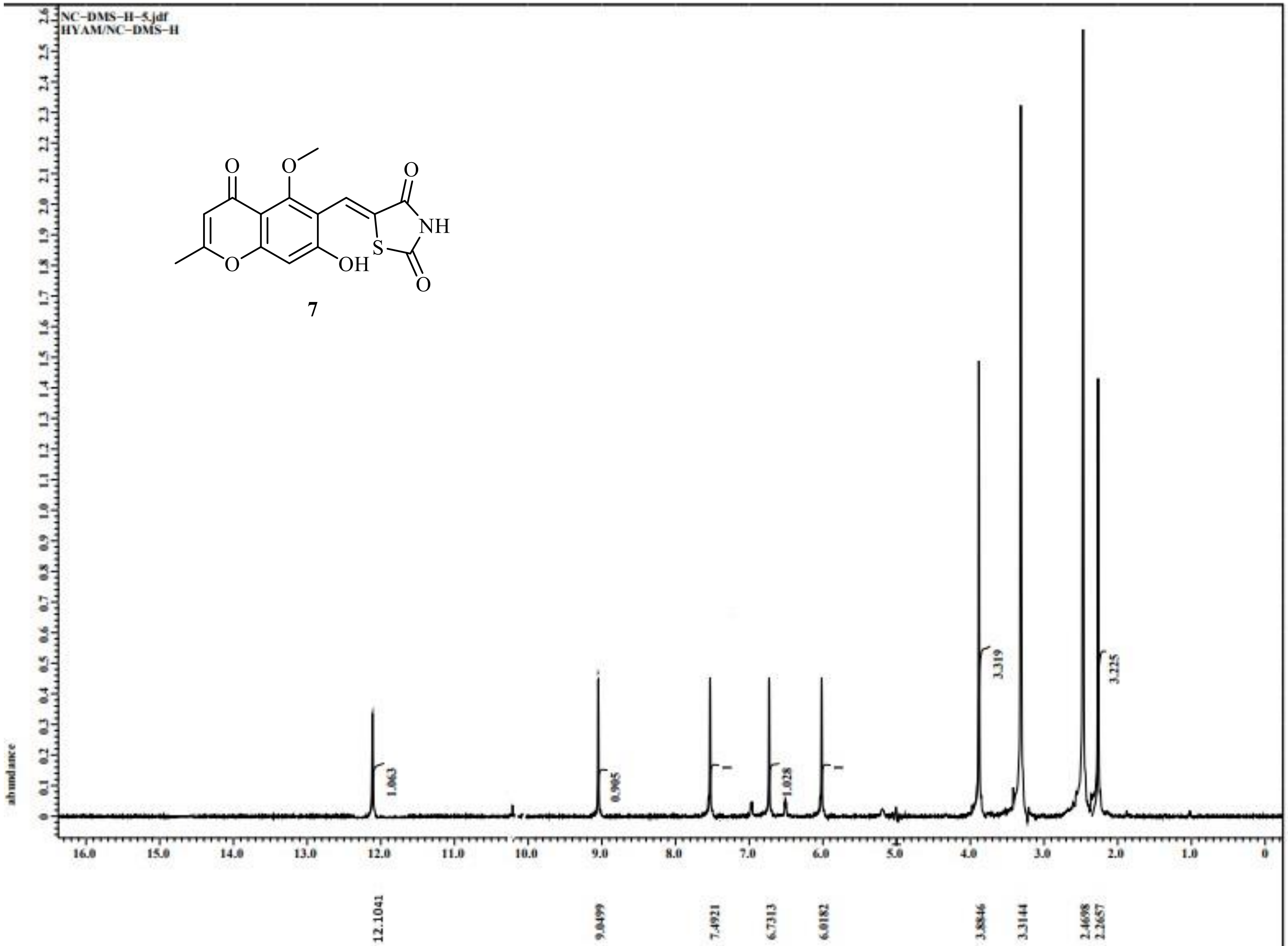

Figure s17: ^{1}HNMR (DMSO) spectrum for compound **7**

Figure s18: Mass spectrum for compound **7**

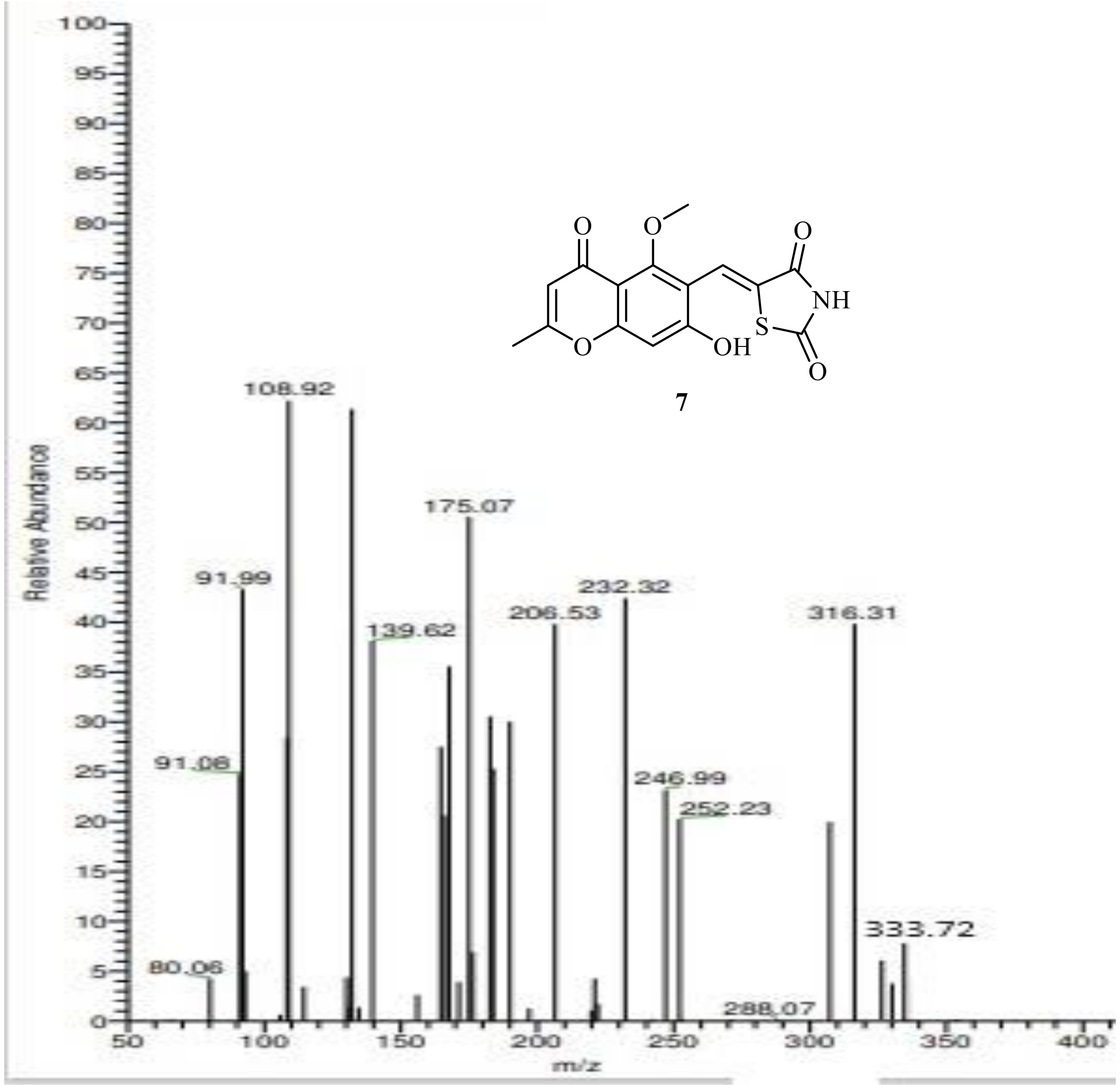

Figure s19: ^{1}HNMR (DMSO) spectrum for compound **8**

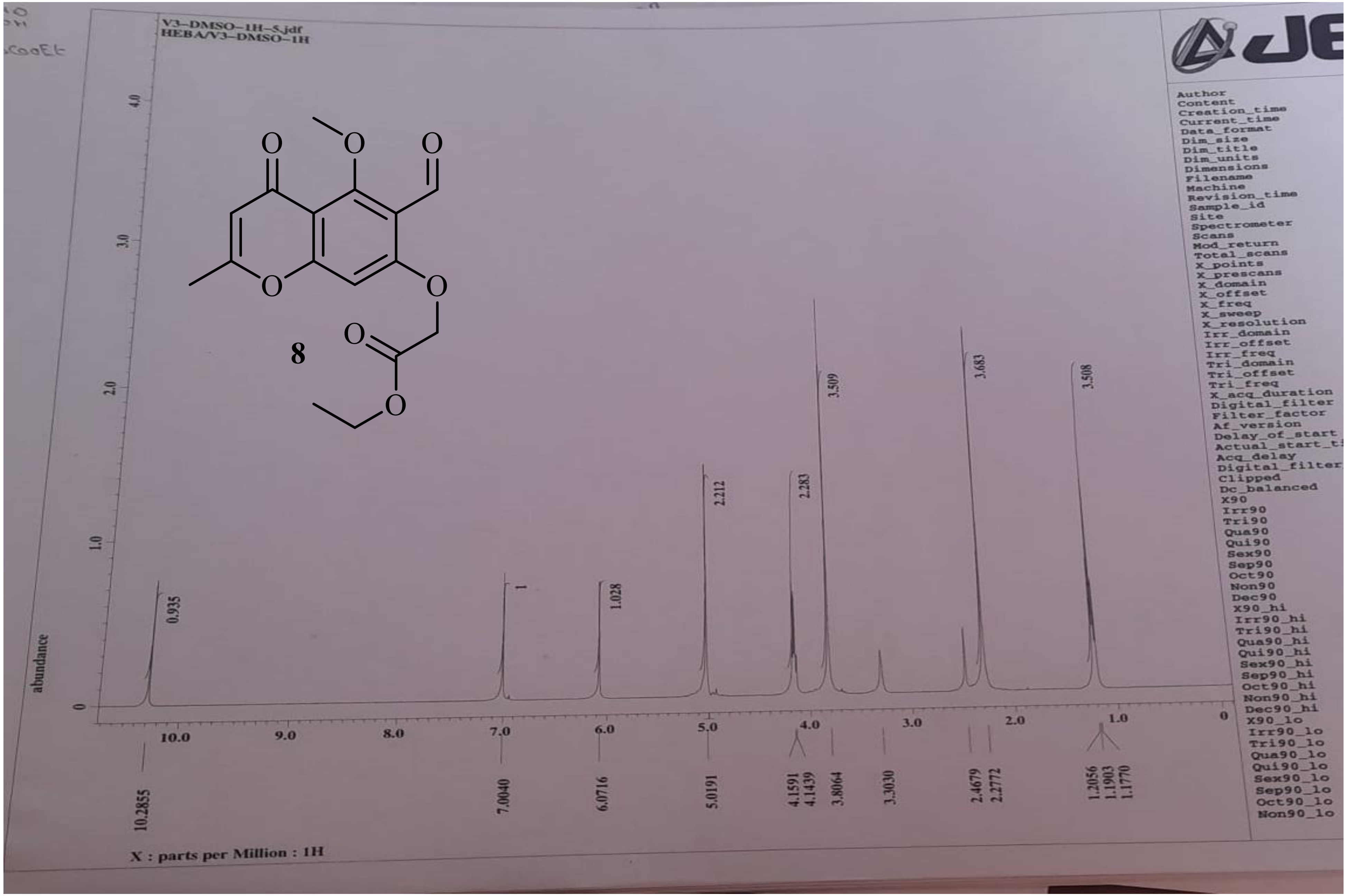

Figure s20: IR spectrum for compound **9**

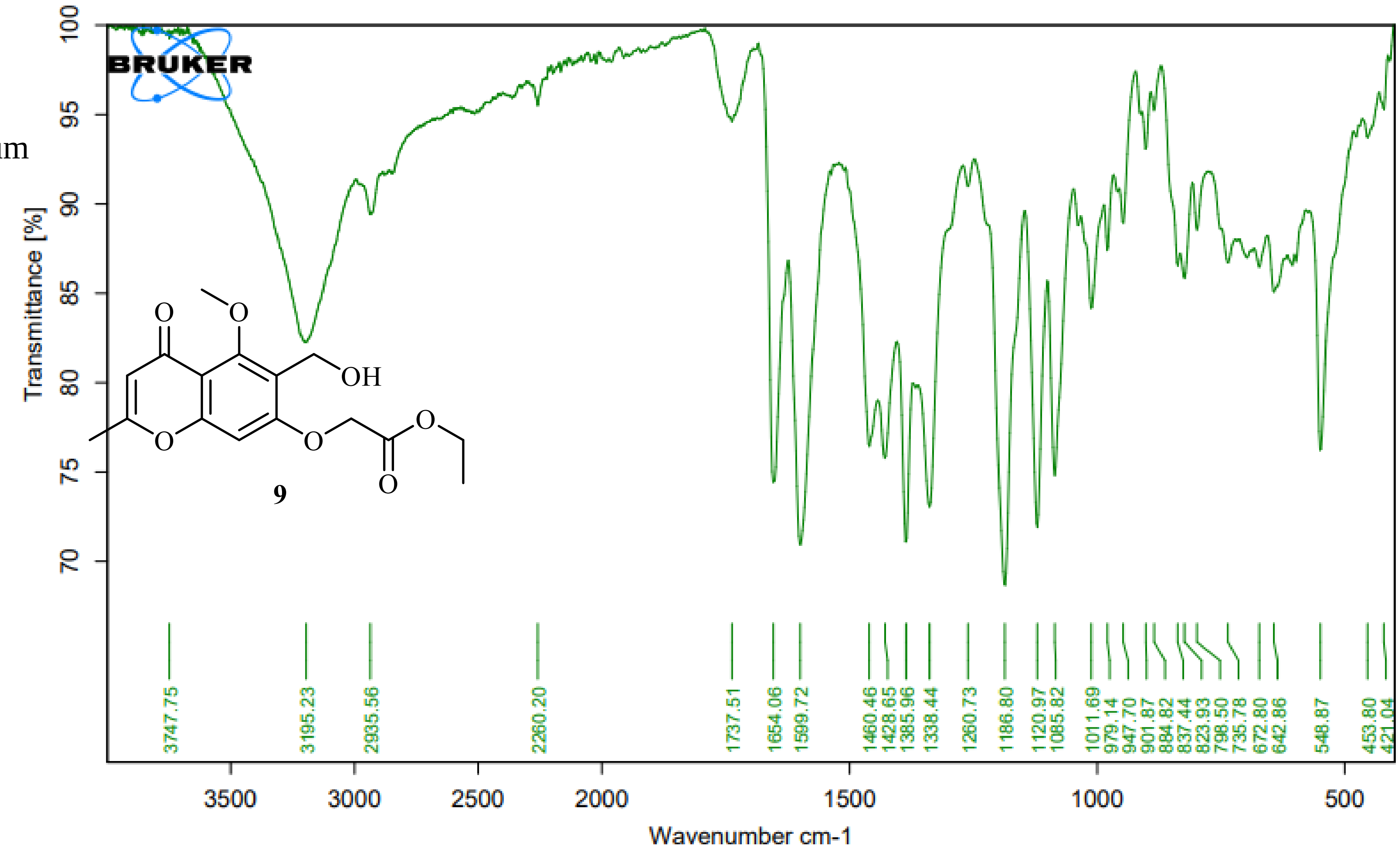

Figure s21: ^{1}HNMR (DMSO) spectrum for compound **9**

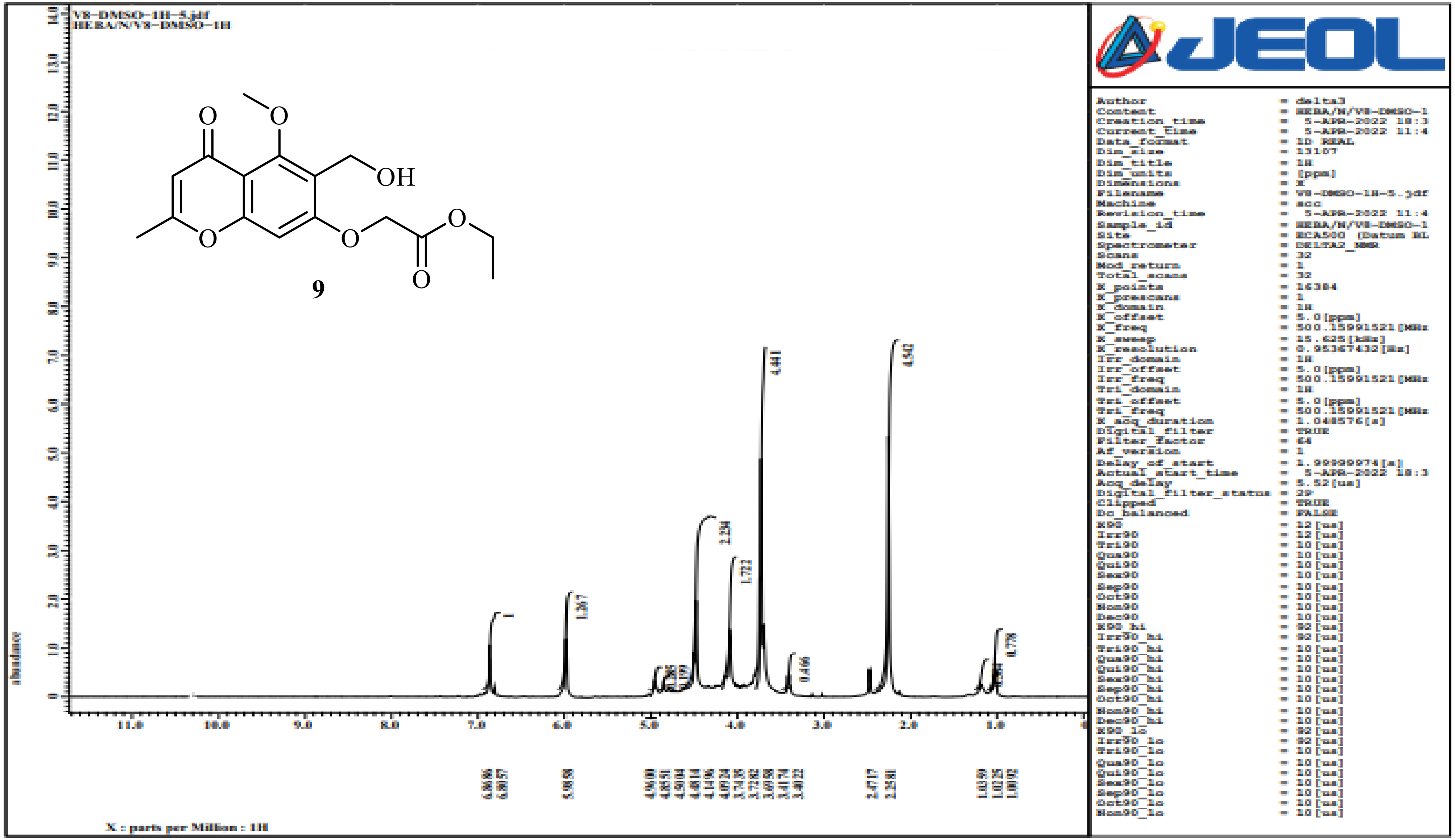

Figure s22: IR spectrum for compound **10**

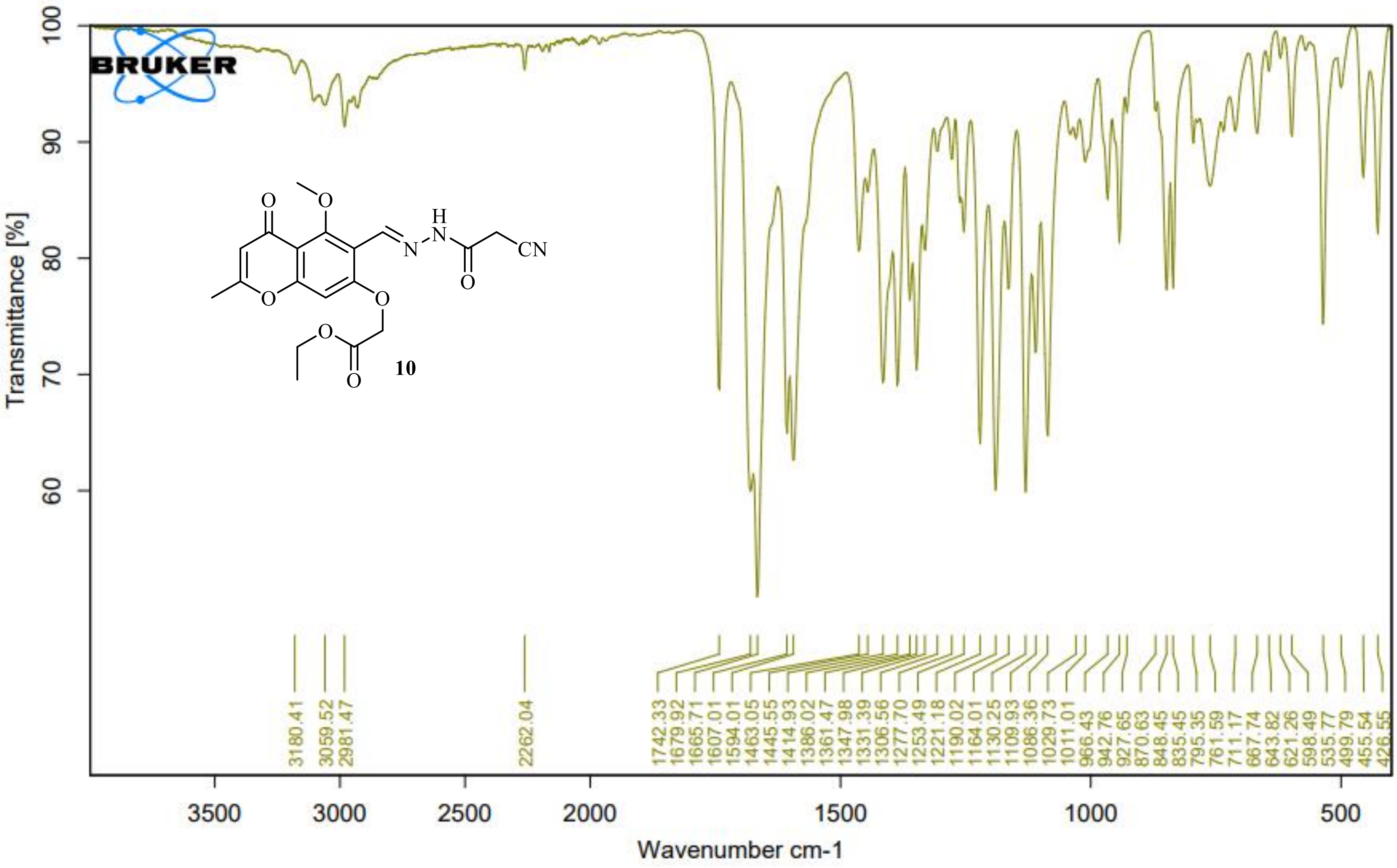

Figure s23: ^{1}HNMR (DMSO) spectrum for compound **10**

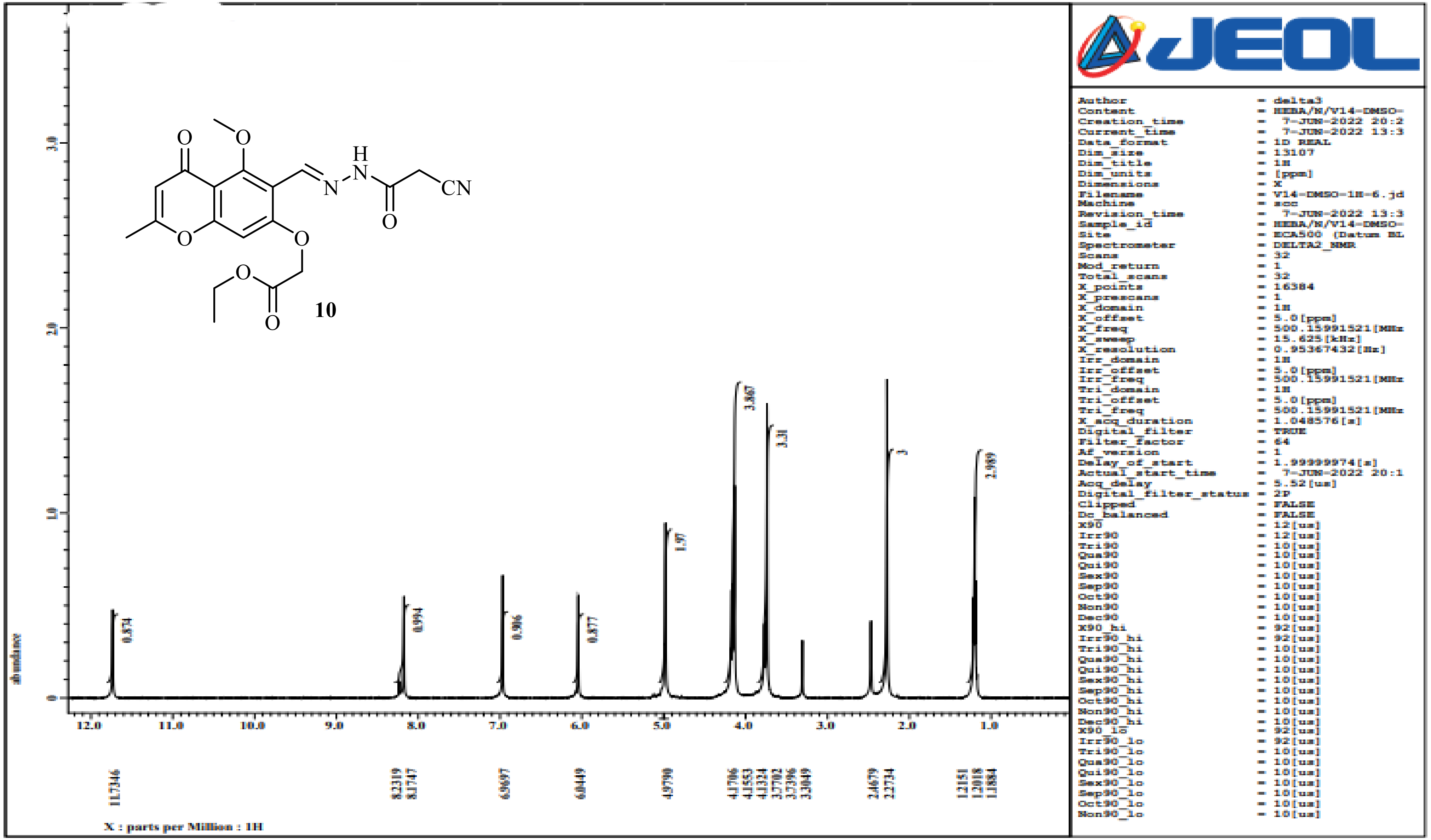

Figure s24: ^{13}CNMR (DMSO) spectrum for compound **10**

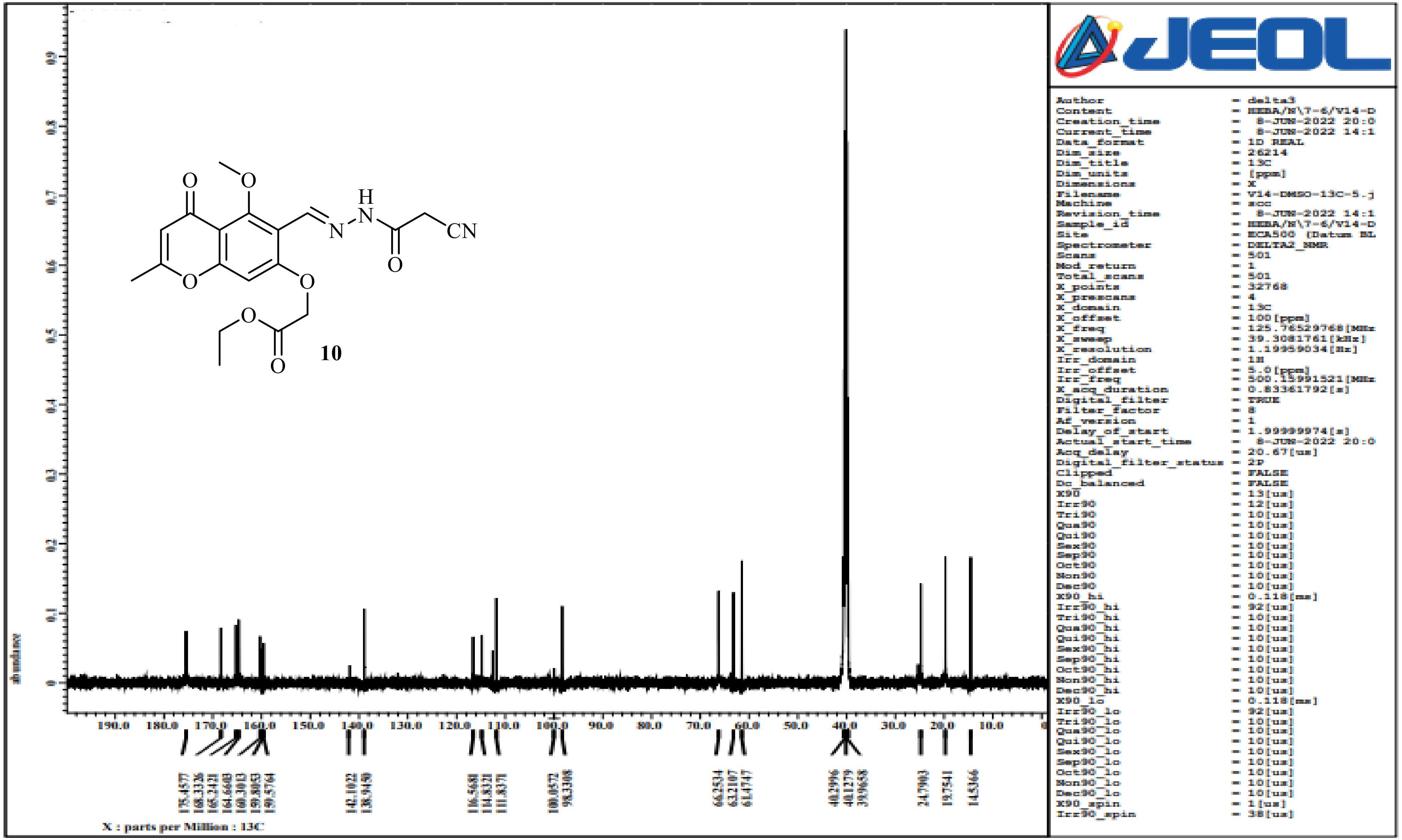

Figure s25: IR spectrum for compound **11**

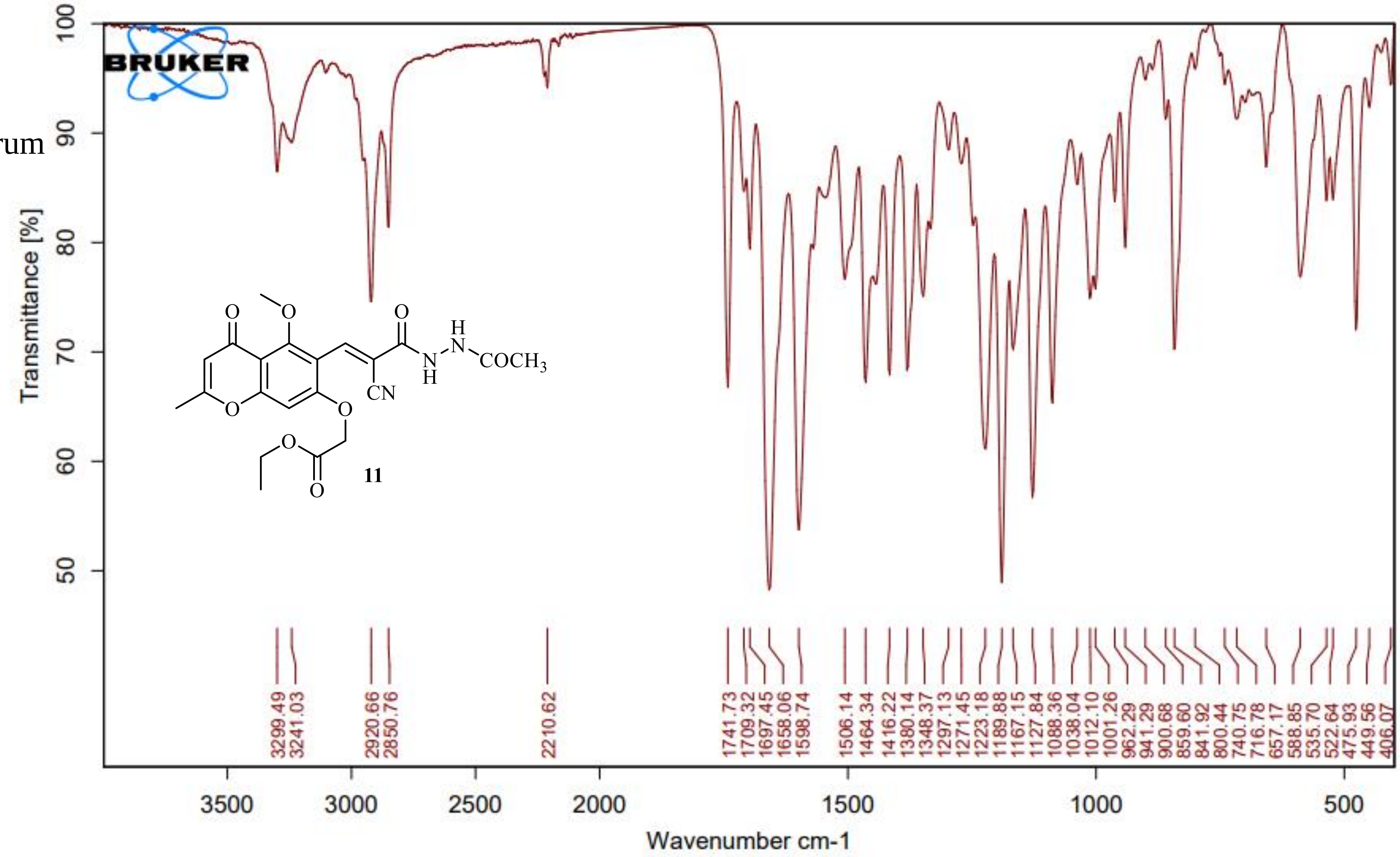

Figure s26: ^{1}HNMR (DMSO) spectrum for compound **11**

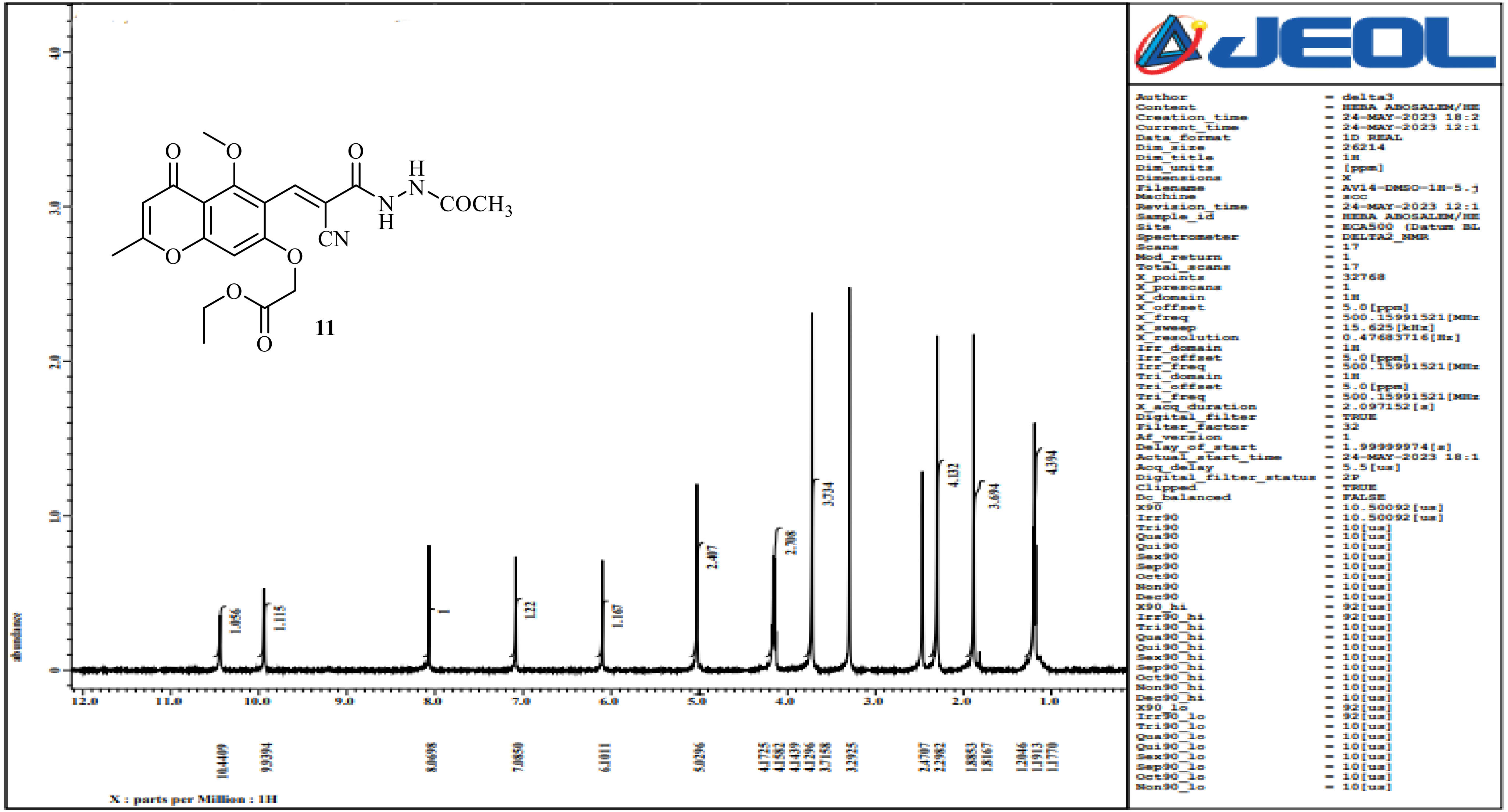

Figure s27: ^{13}CNMR (DMSO) spectrum for compound **11**

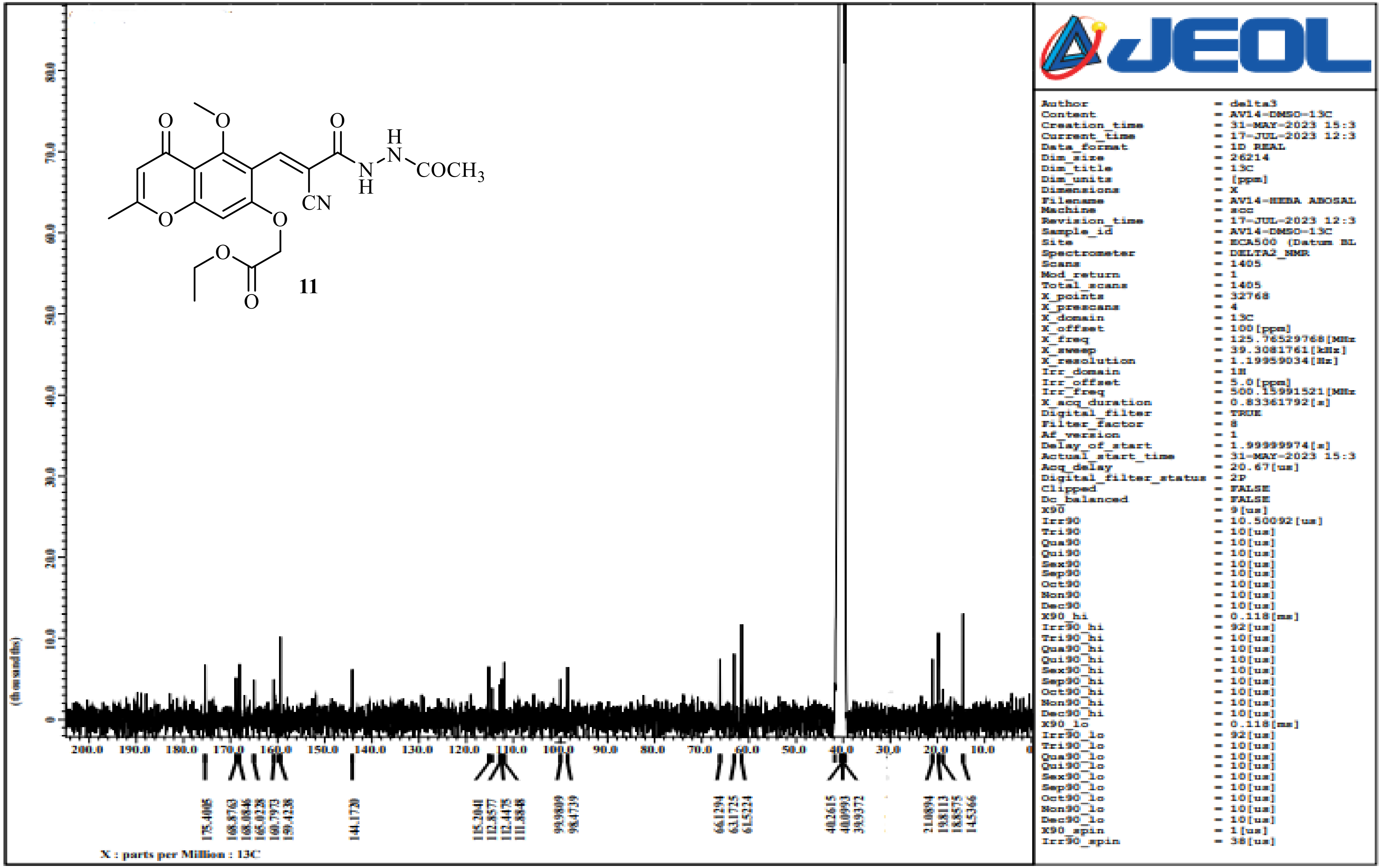

Figure s28: IR spectrum for compound **12**

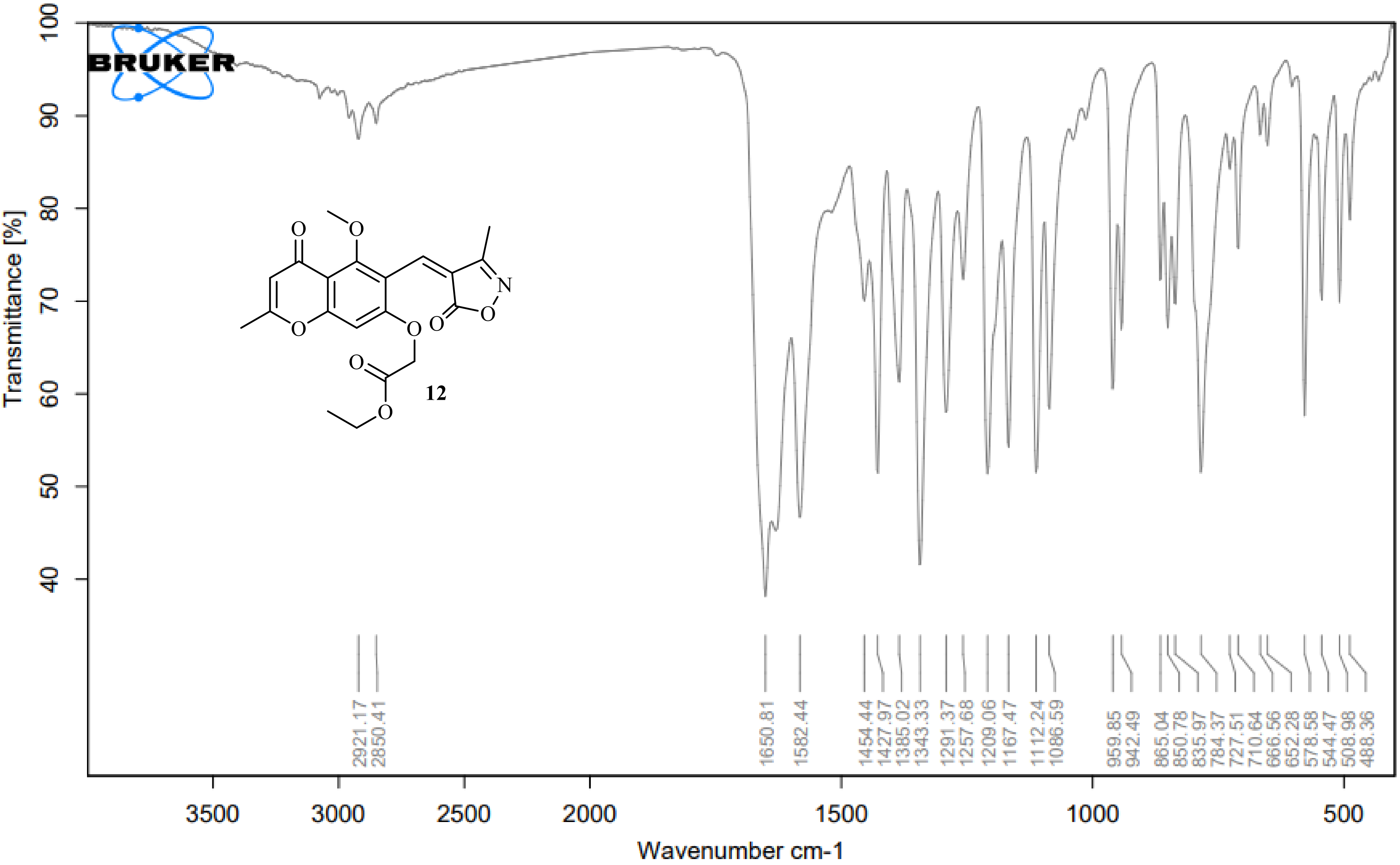

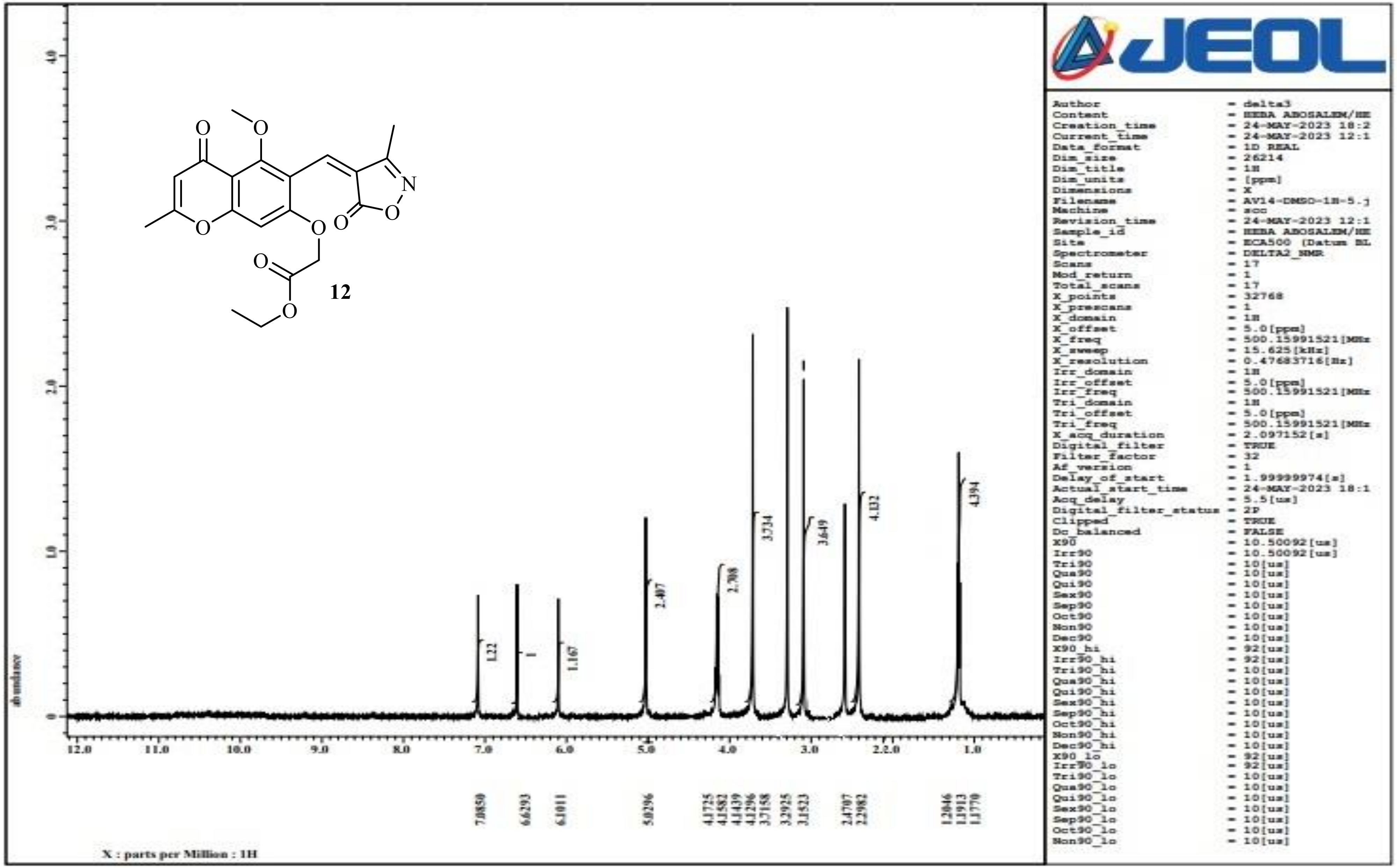

Figure s29: ^{1}HNMR (DMSO) spectrum for compound **12**

Figure s30: Mass spectrum for compound 12

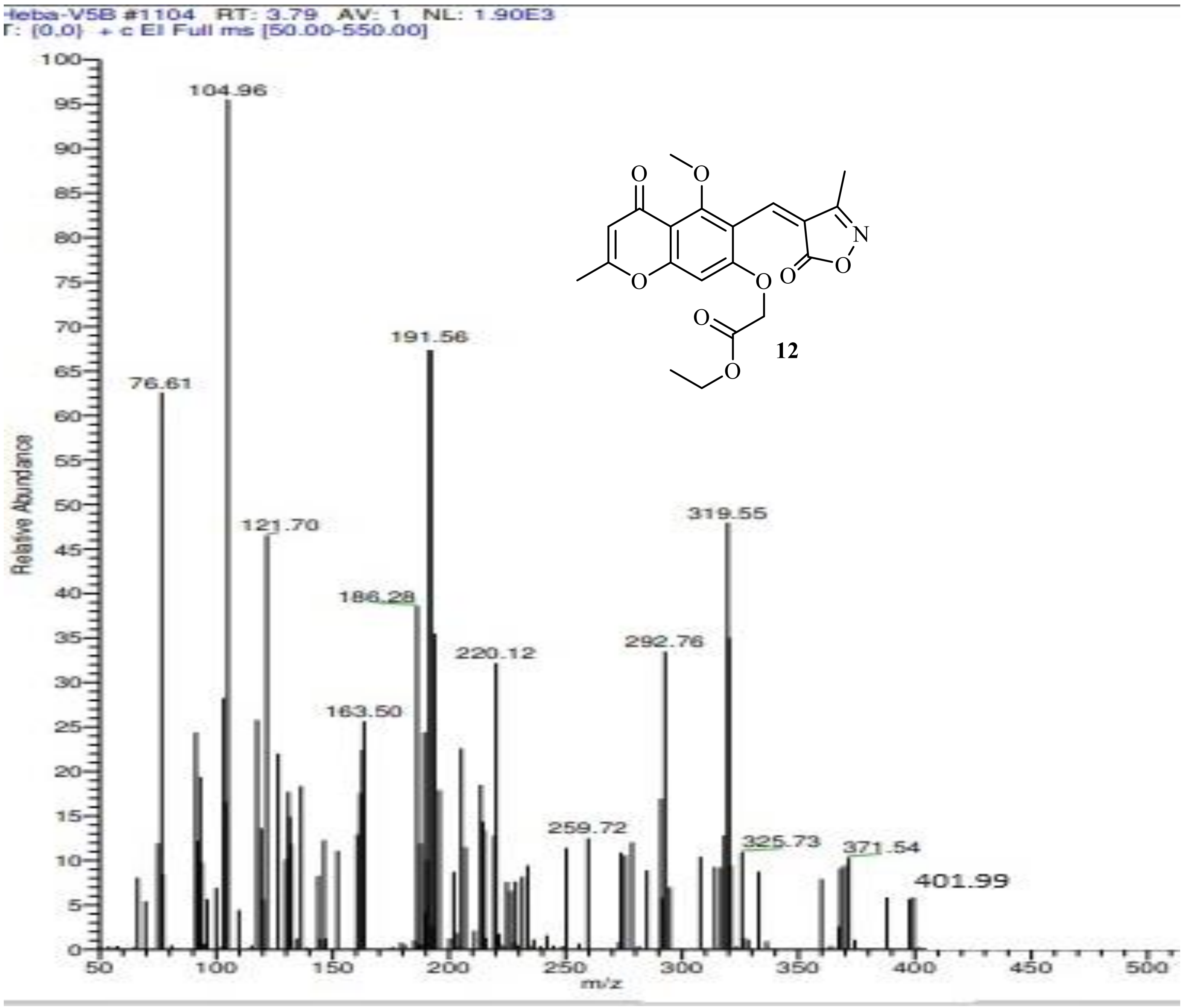

Figure s31: IR spectrum for compound **13**

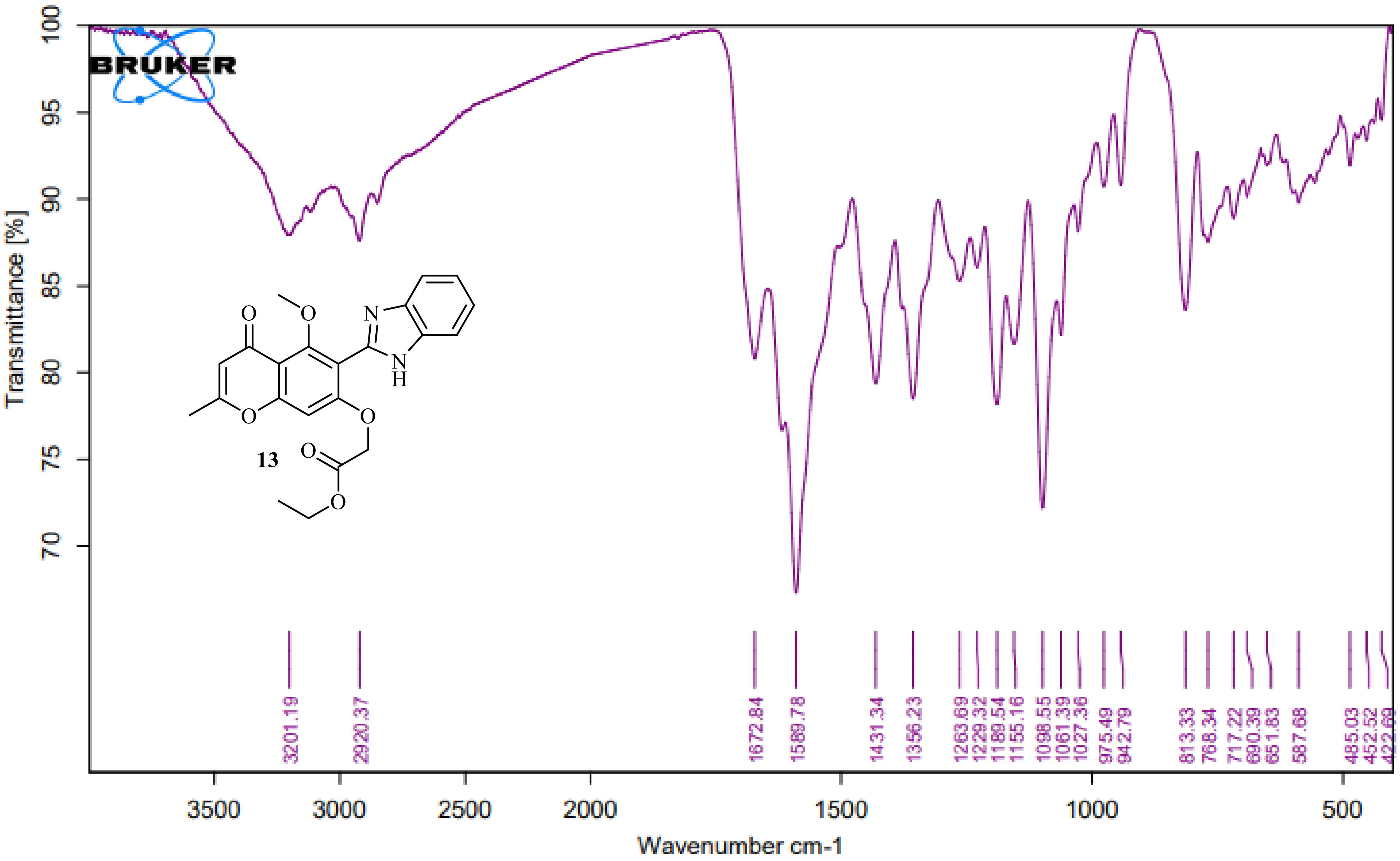

Figure s32: ^{1}HNMR (DMSO) spectrum for compound **13**

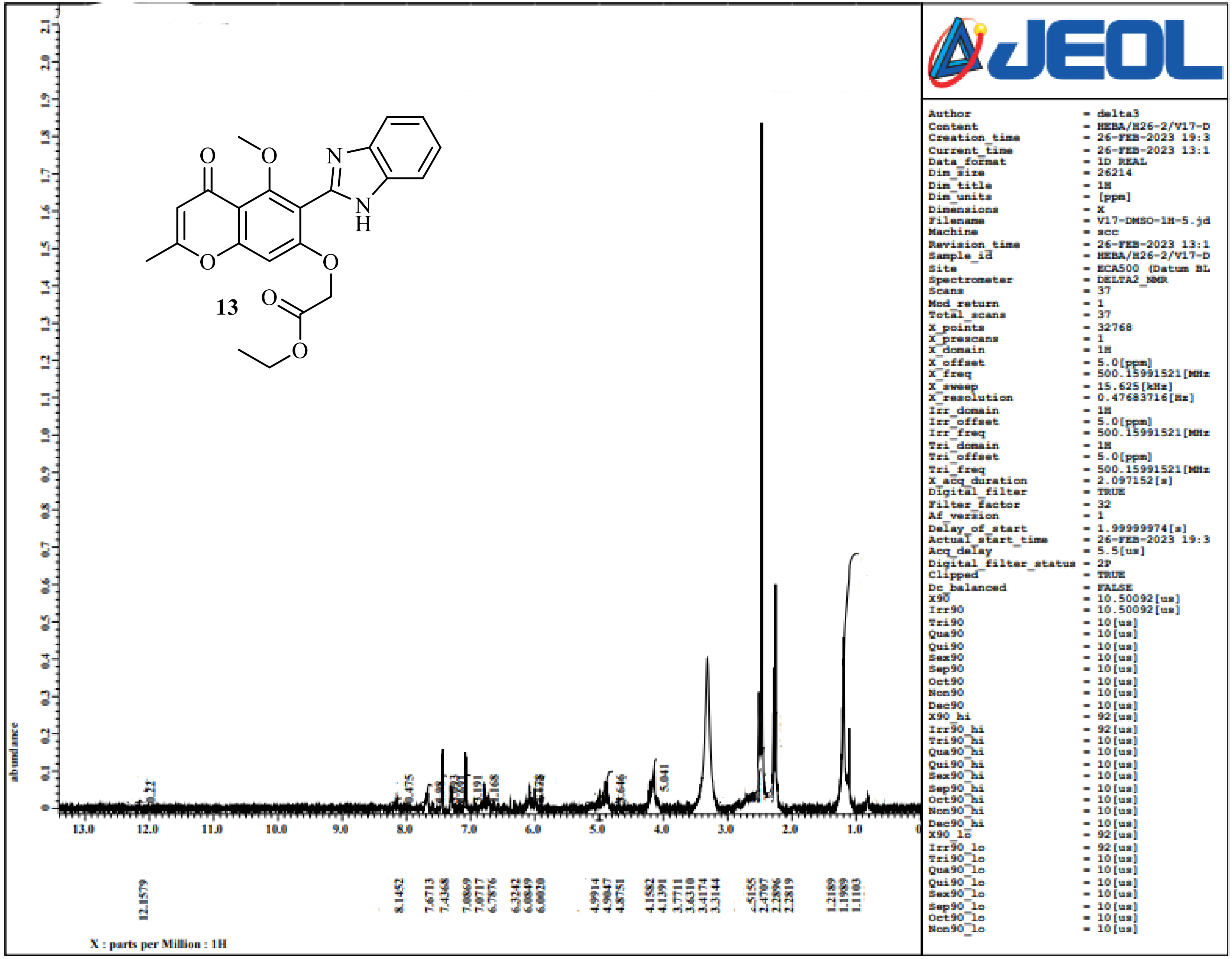

Figure s33: Mass spectrum for compound **13**

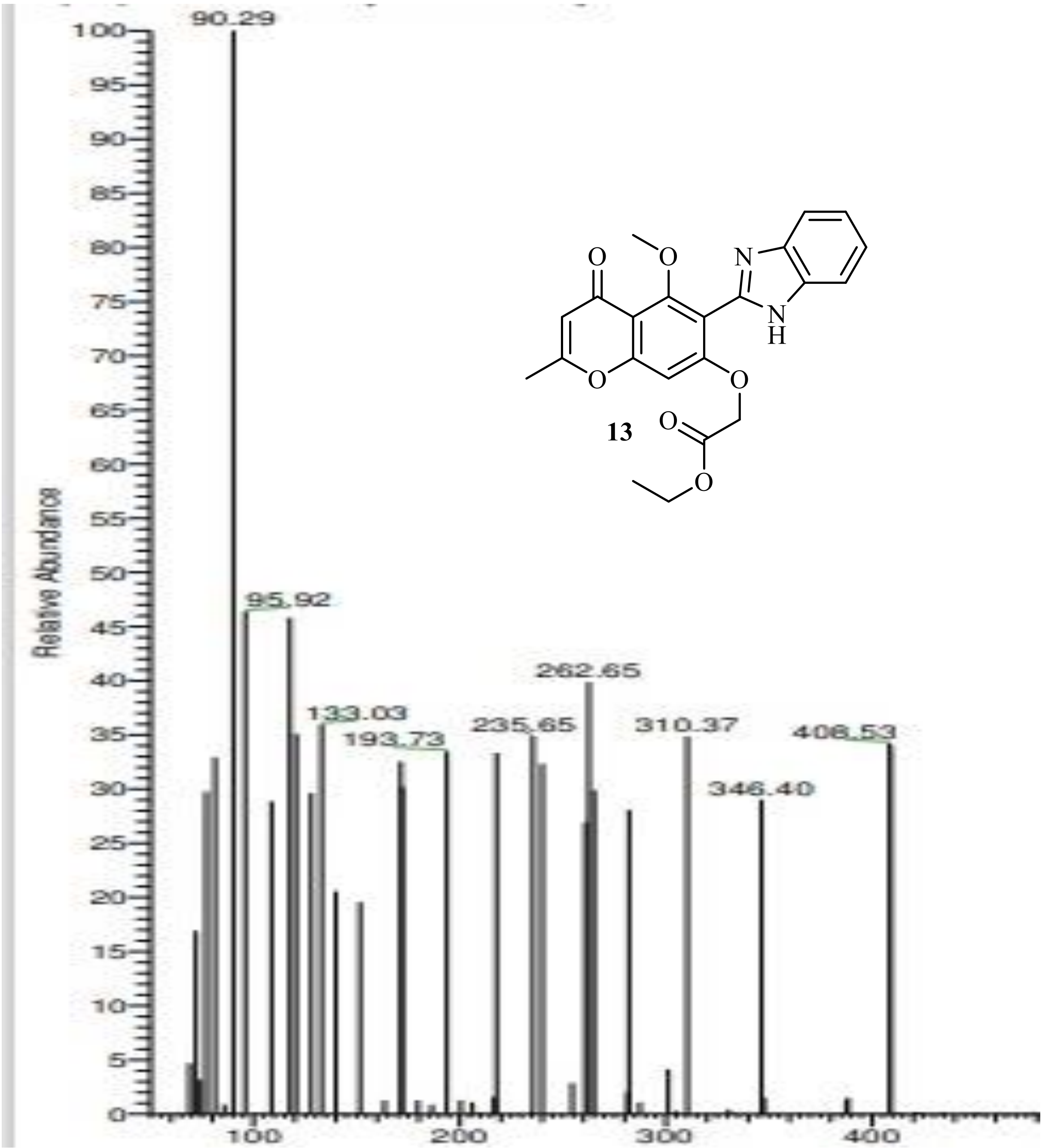

Figure s34: IR spectrum for compound **14a**

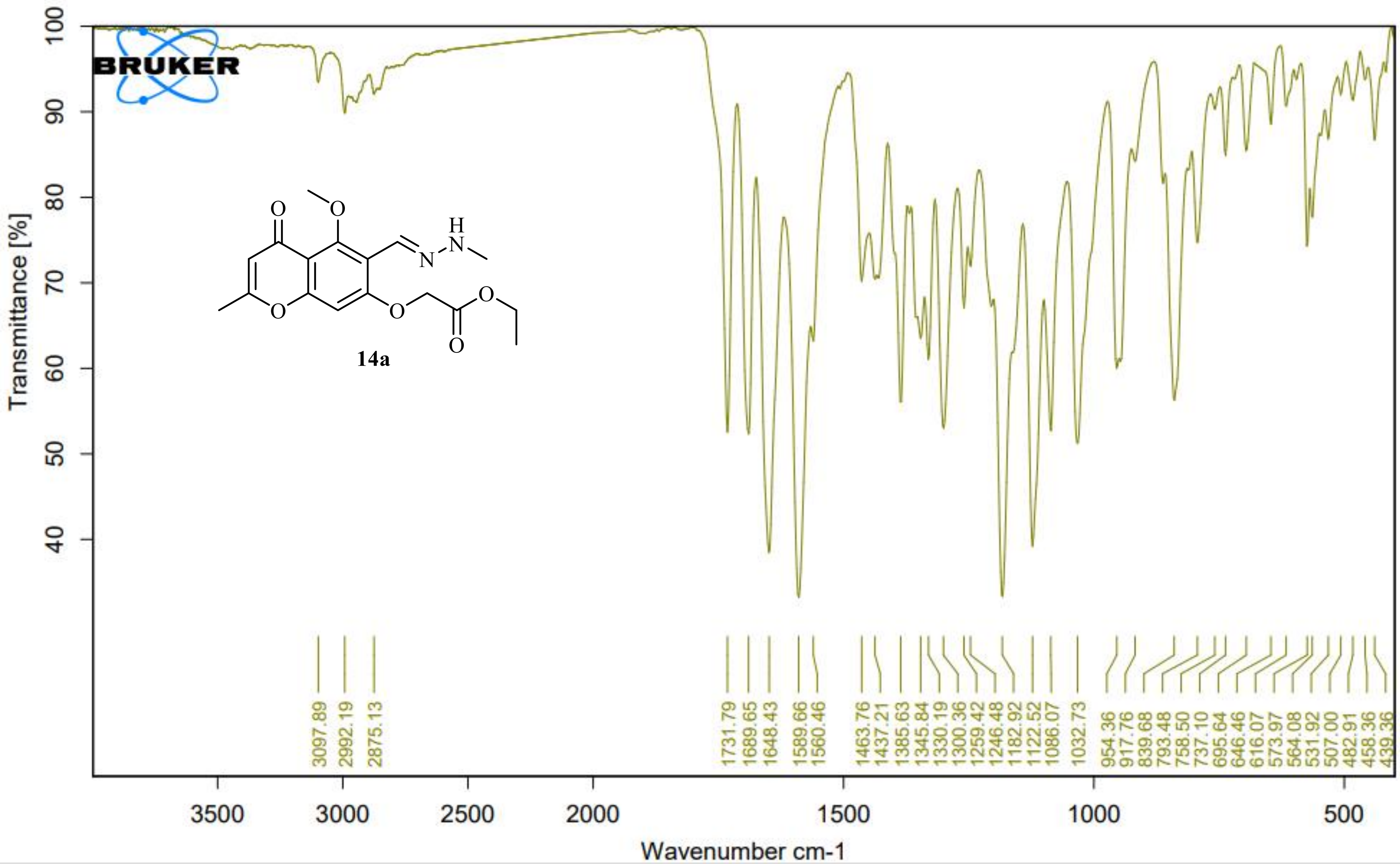

Figure s35: ^{1}HNMR (DMSO) spectrum for compound **14a**

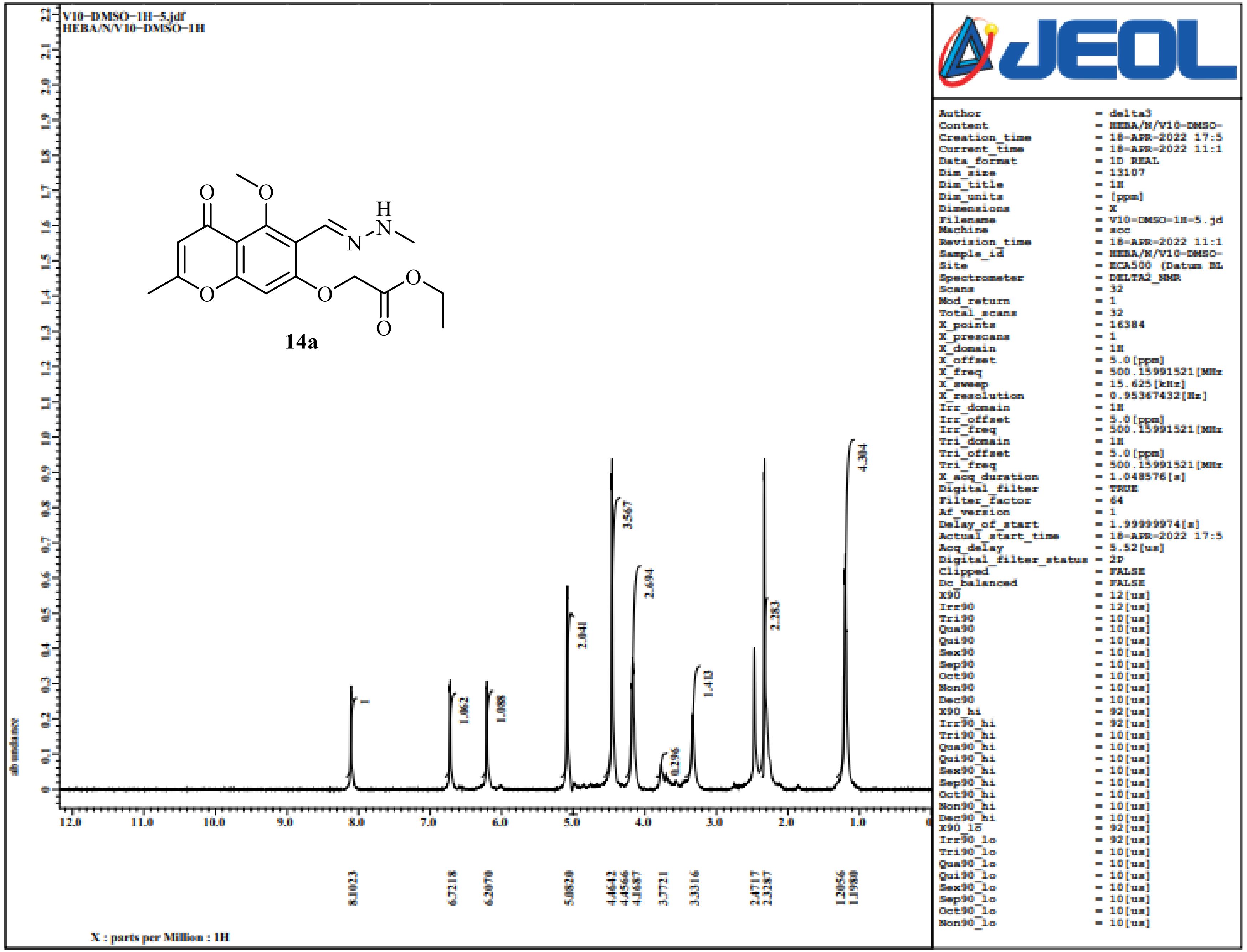

Figure s36: IR spectrum for compound **14b**

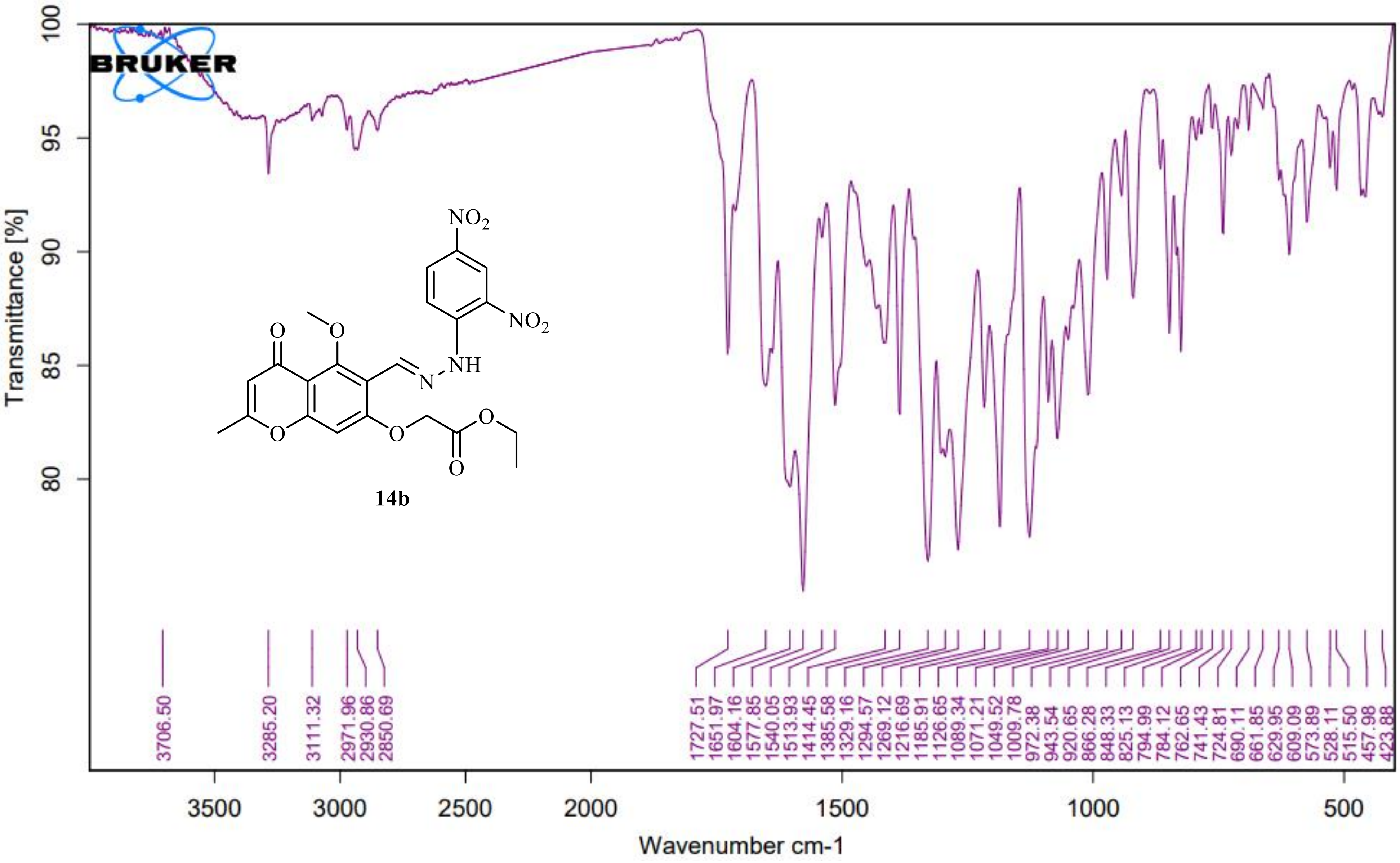

Figure s37: ^{1}HNMR (DMSO) spectrum for compound **14b**

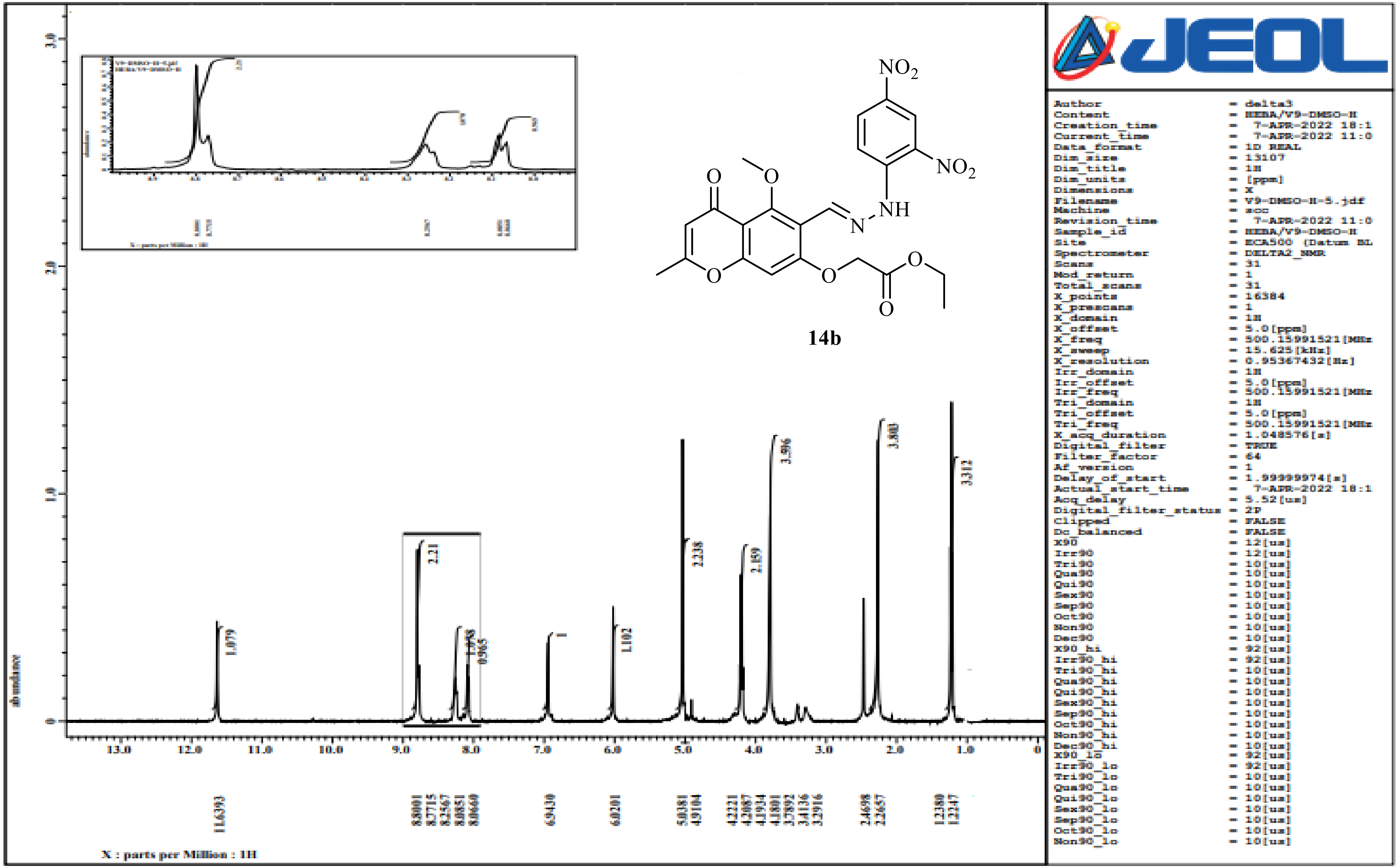

Figure s38: Mass spectrum for compound 14b

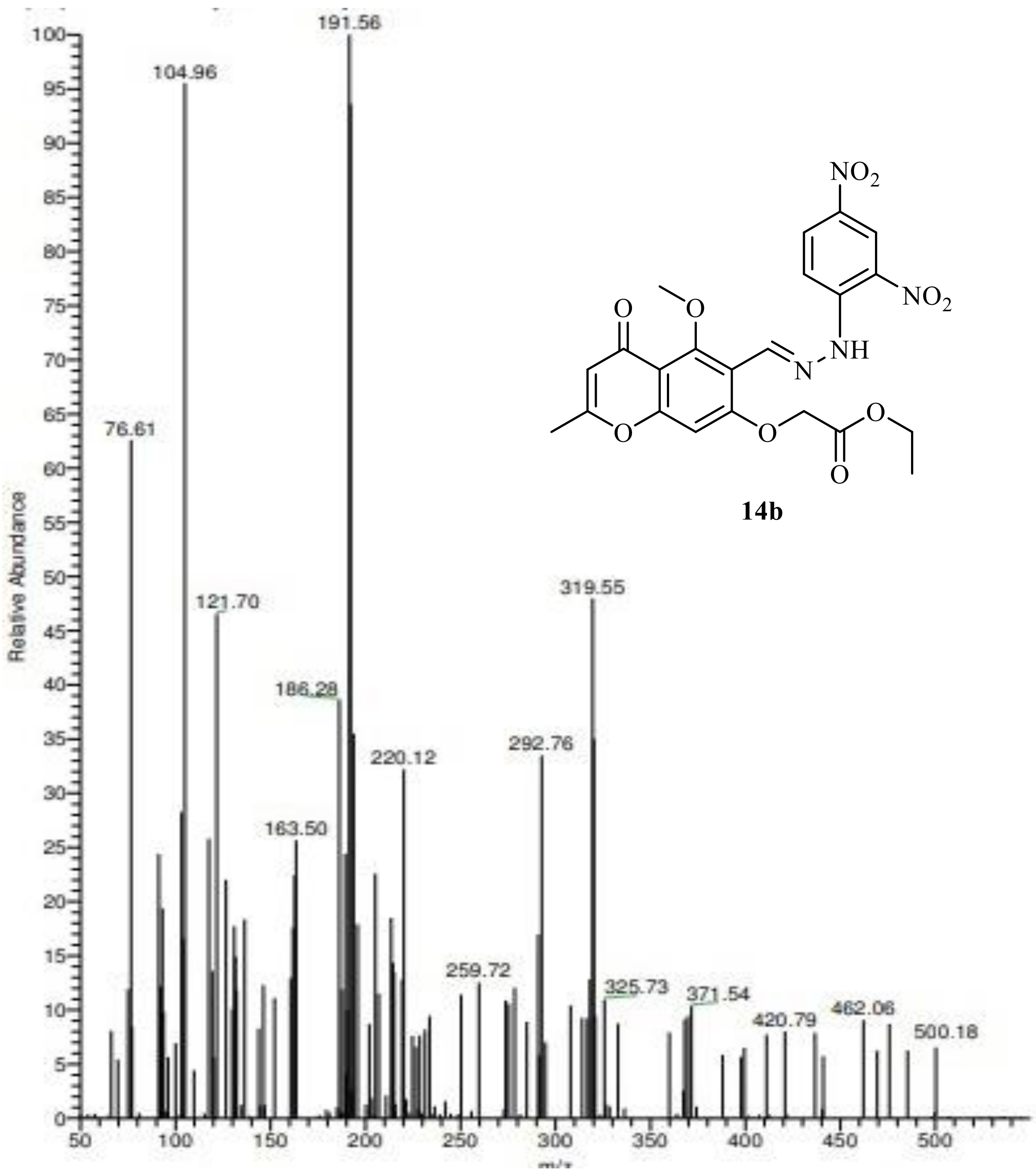

14b

Figure s39: IR spectrum for compound **14c**

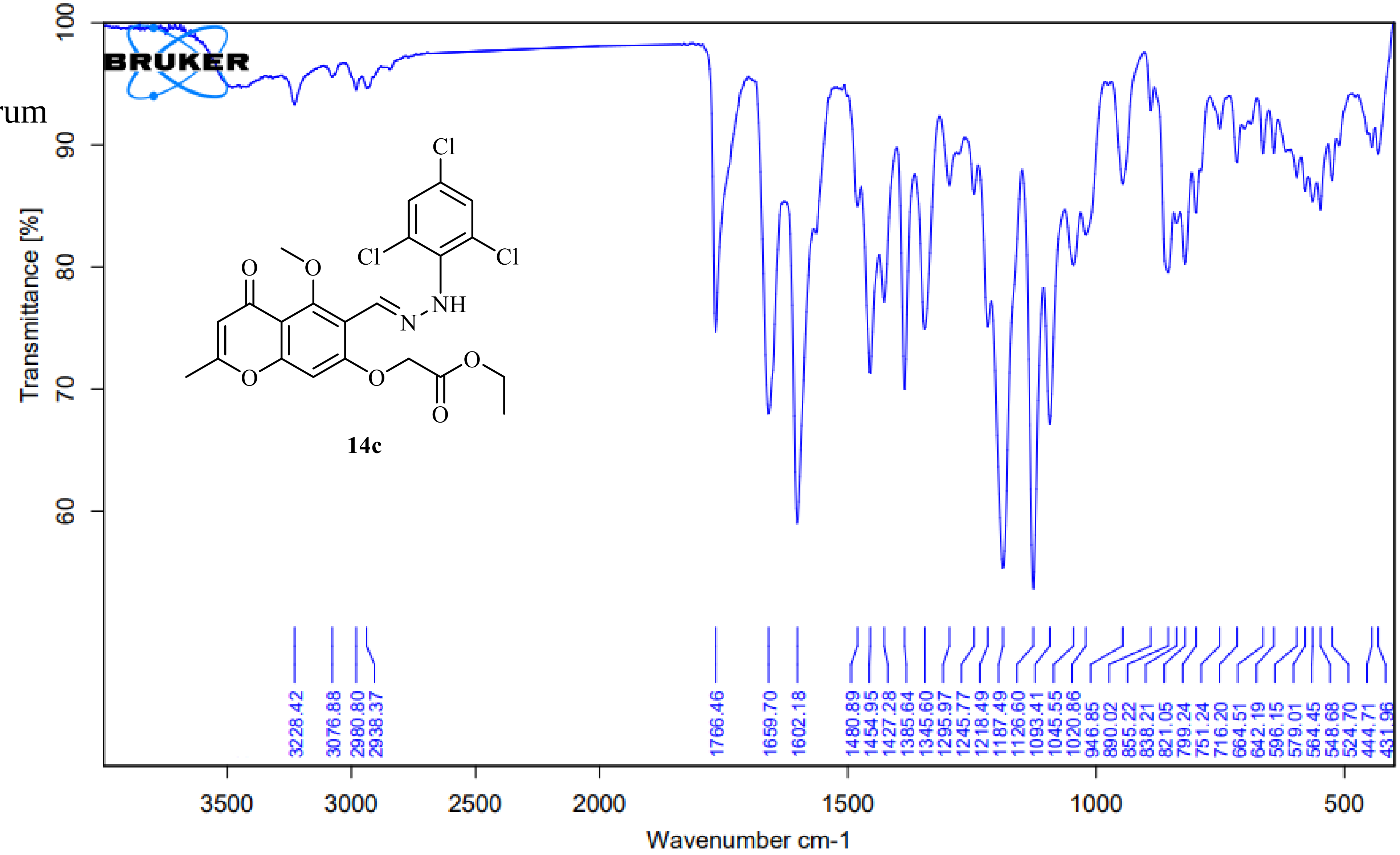

Figure s40: ^{1}HNMR (DMSO) spectrum for compound **14c**

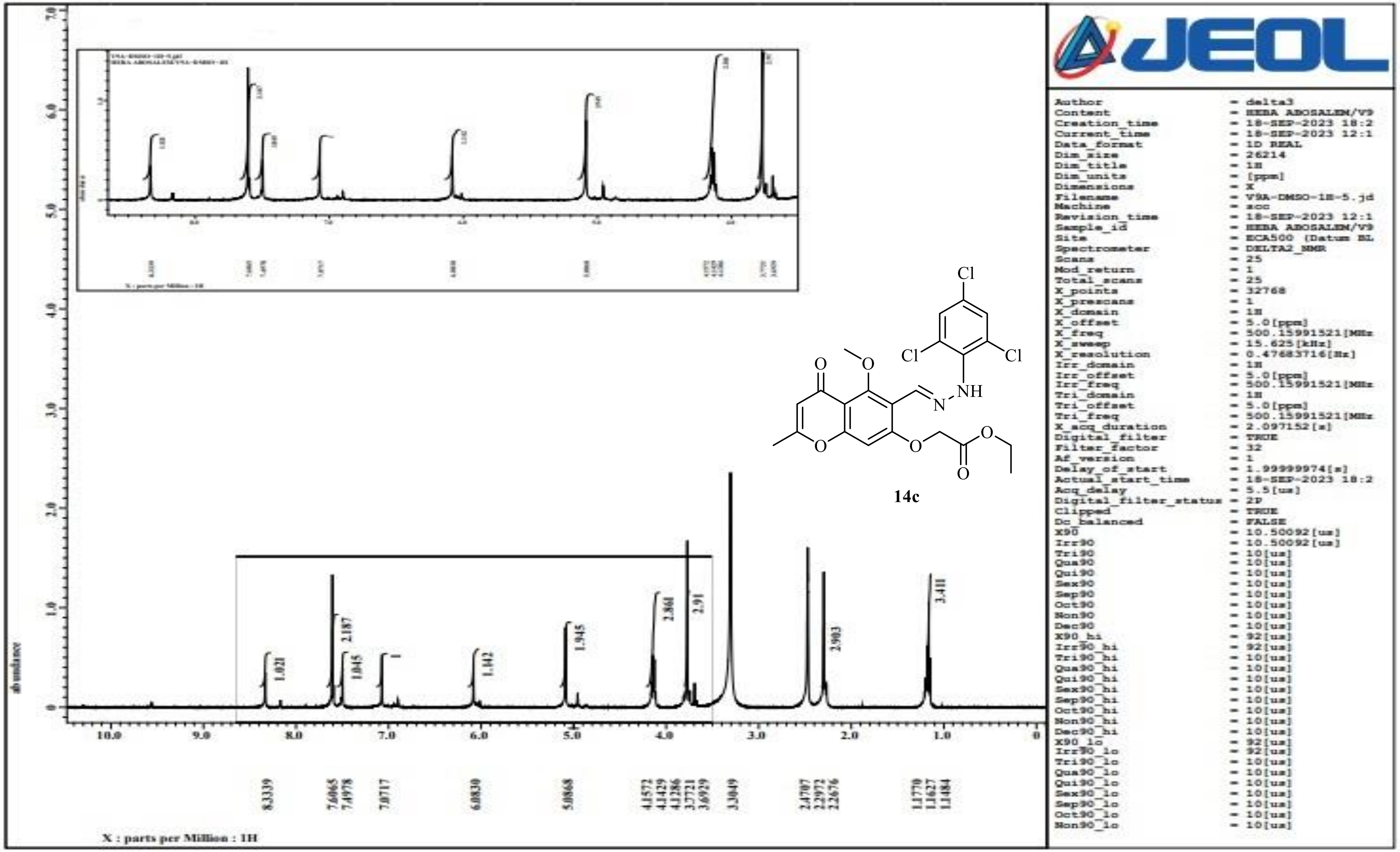

Figure s41: ^{13}CNMR (DMSO) spectrum for compound **14c**

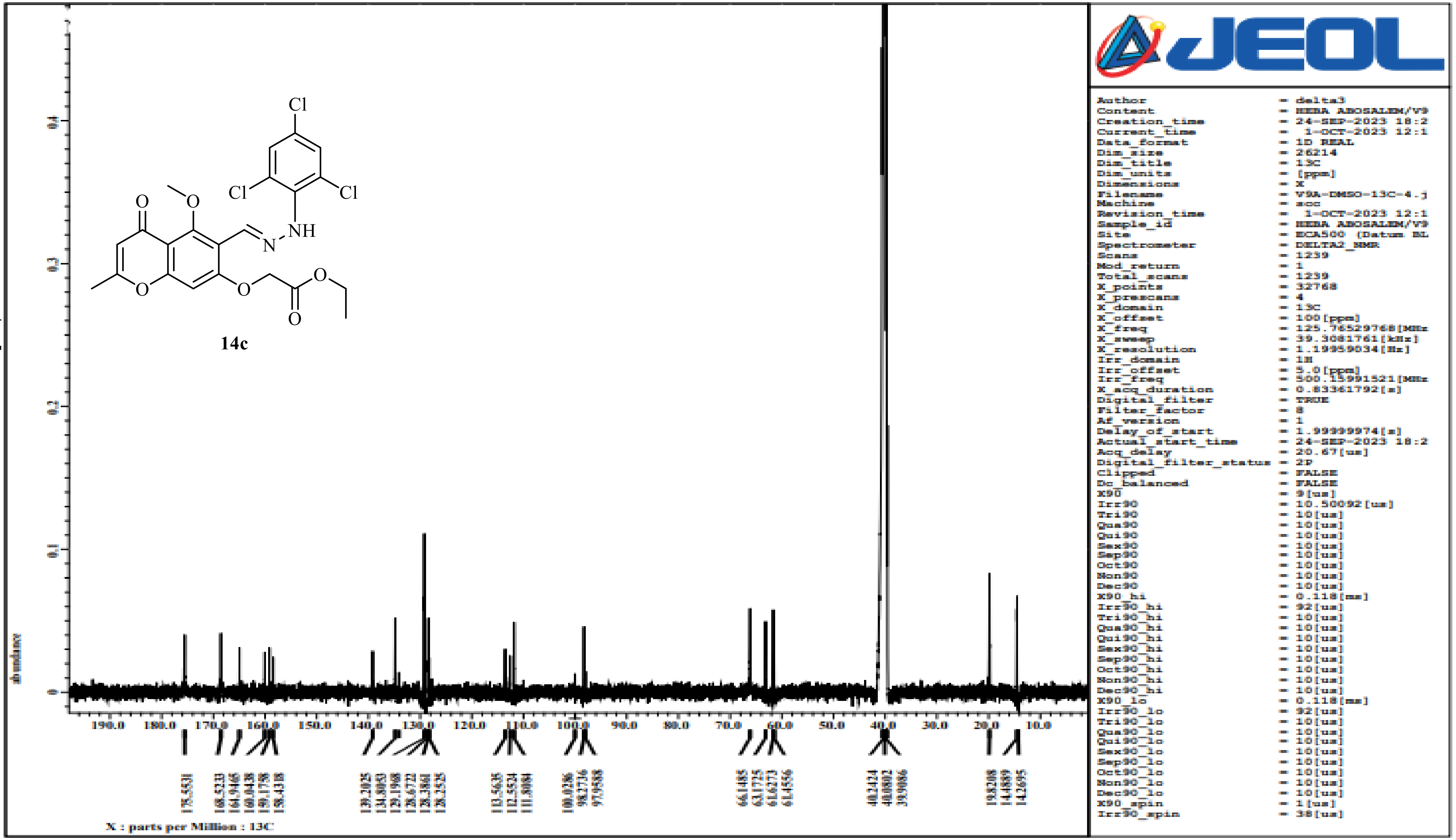

Figure s42: IR spectrum for compound **15a**

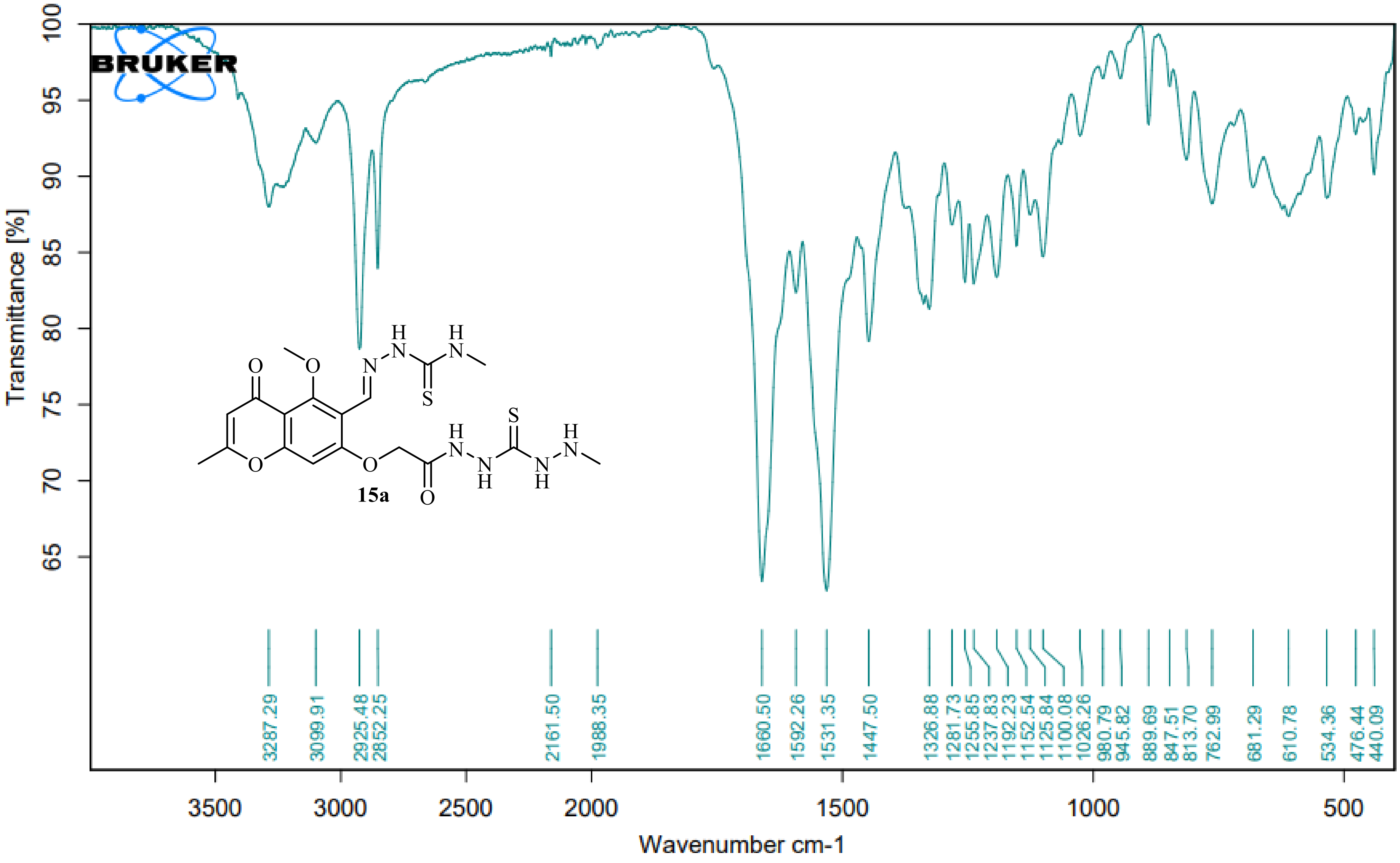

Figure s43: ^{1}HNMR (DMSO) spectrum for compound **15a**

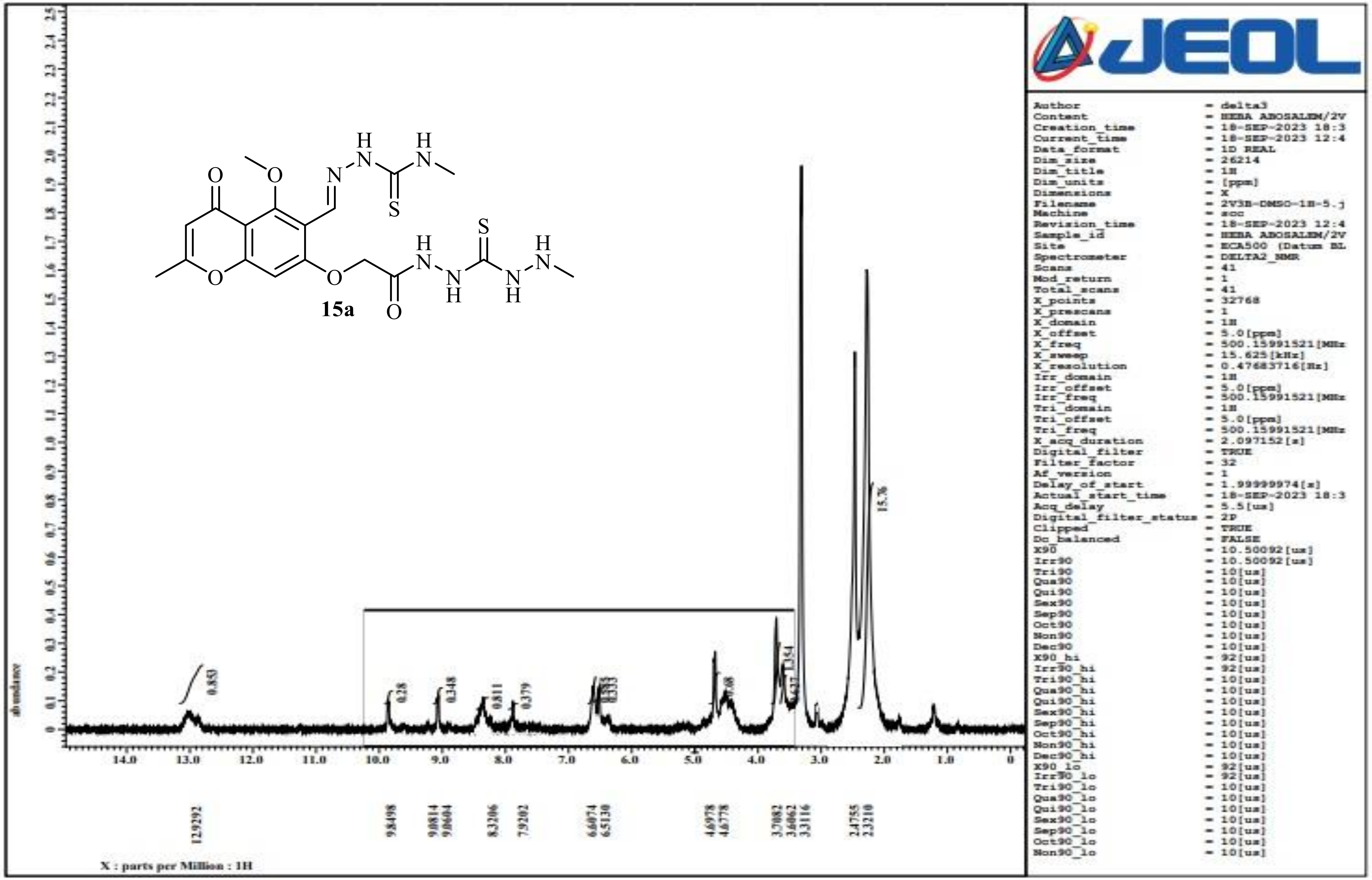

Figure s44: ^{13}CNMR (DMSO) spectrum for compound **15a**

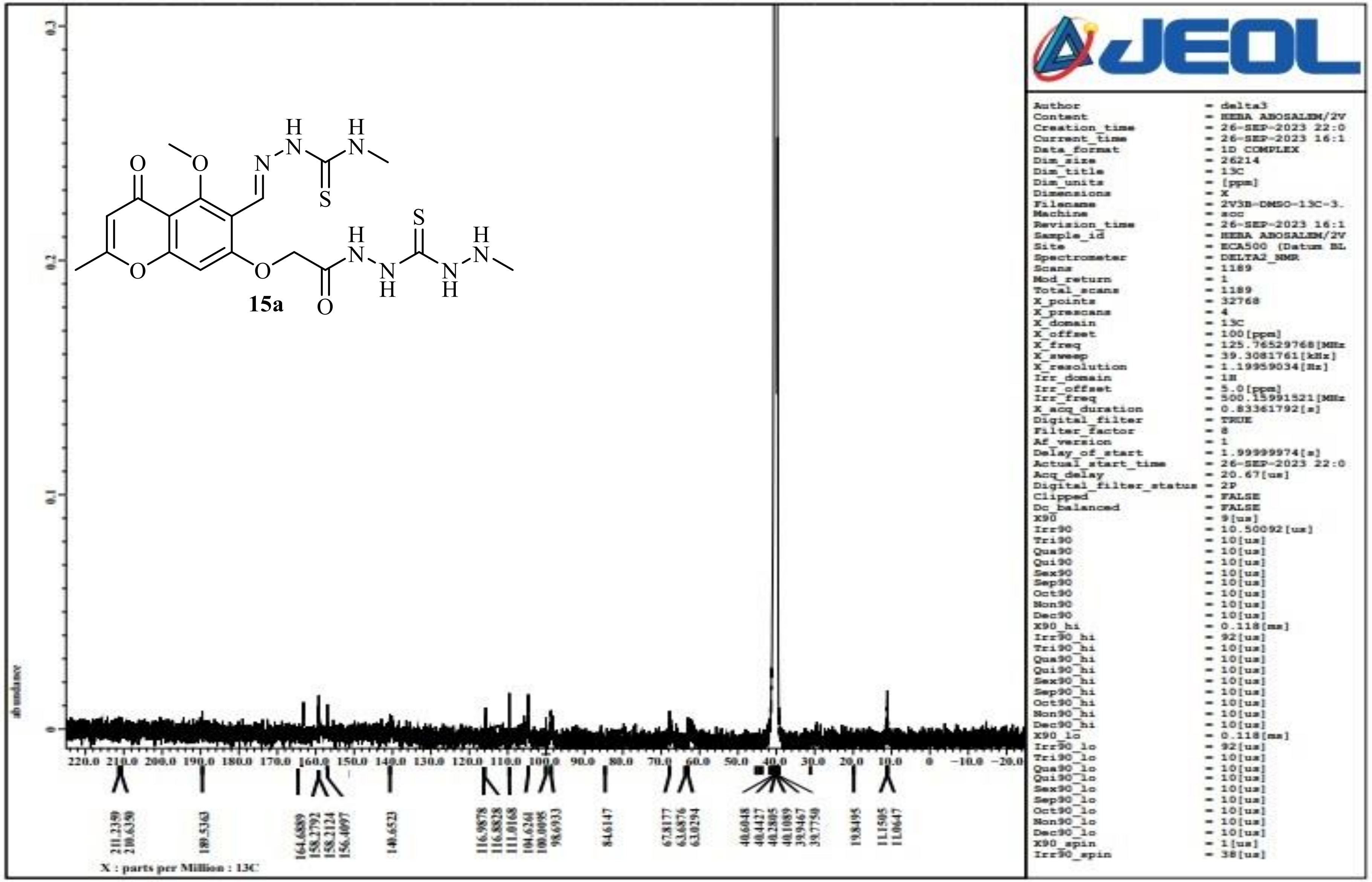

Figure s45: Mass spectrum for compound 15a

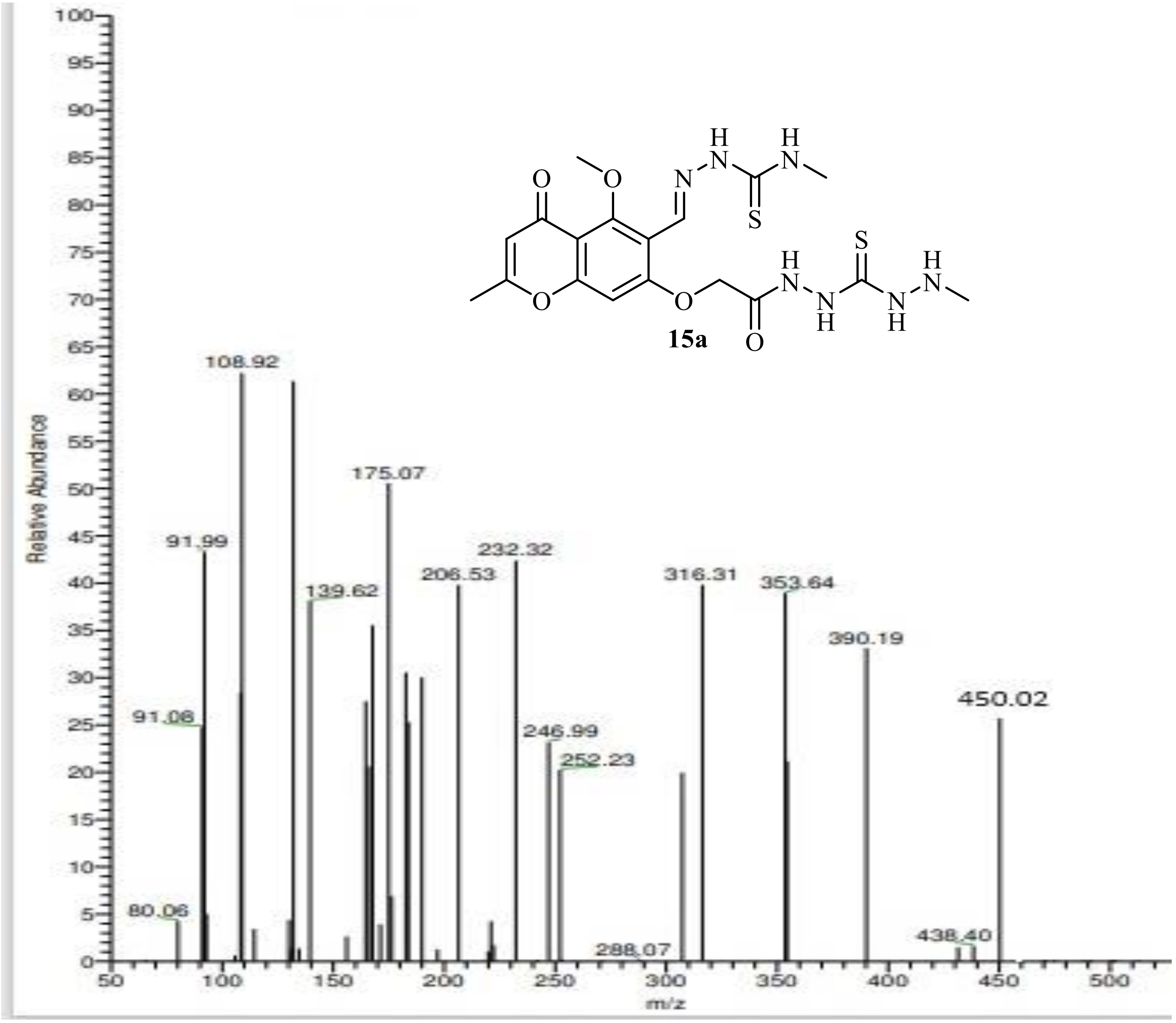

Figure s46: IR spectrum for compound **15b**

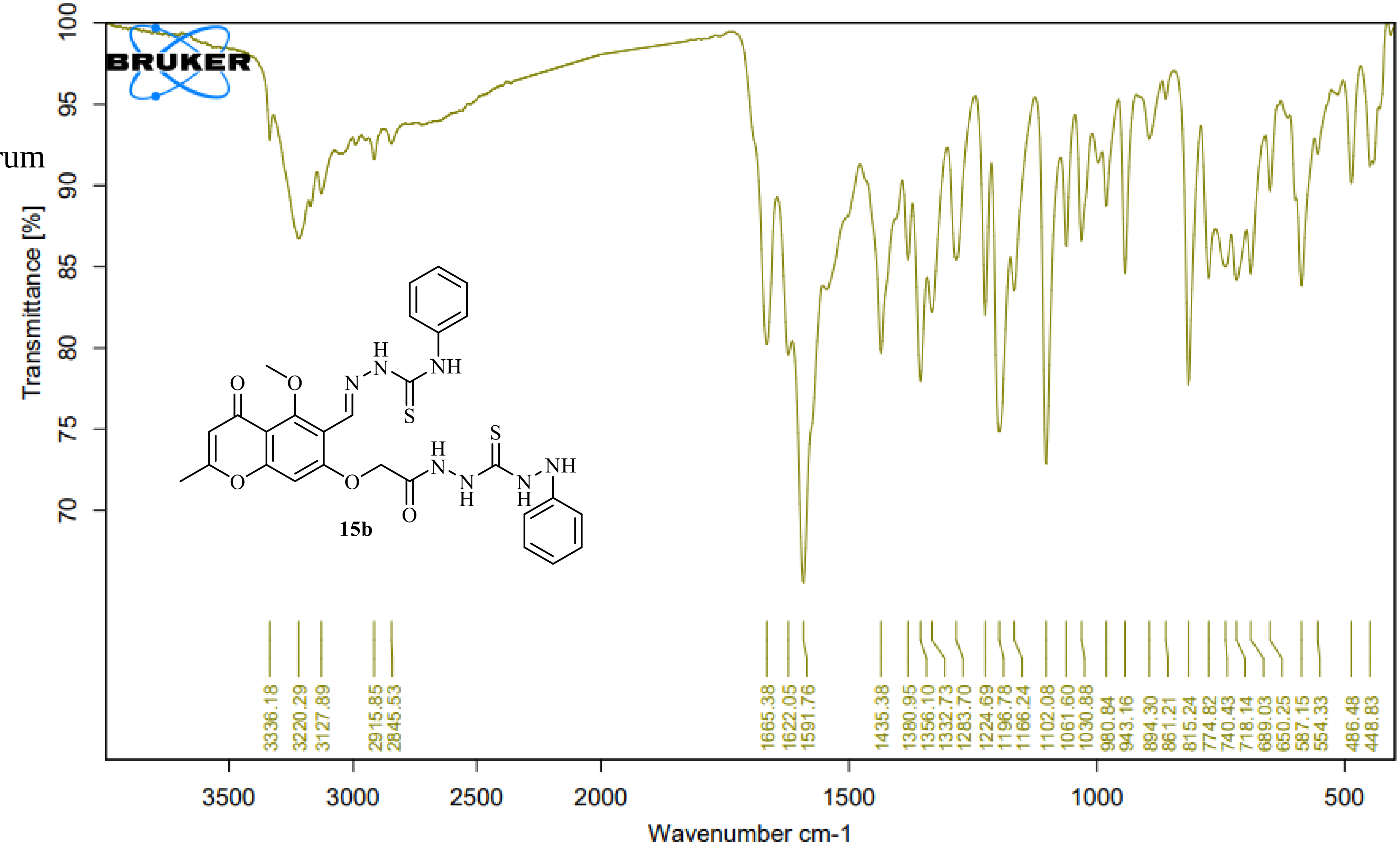

Figure s47: ^{1}HNMR (DMSO) spectrum for compound **15b**

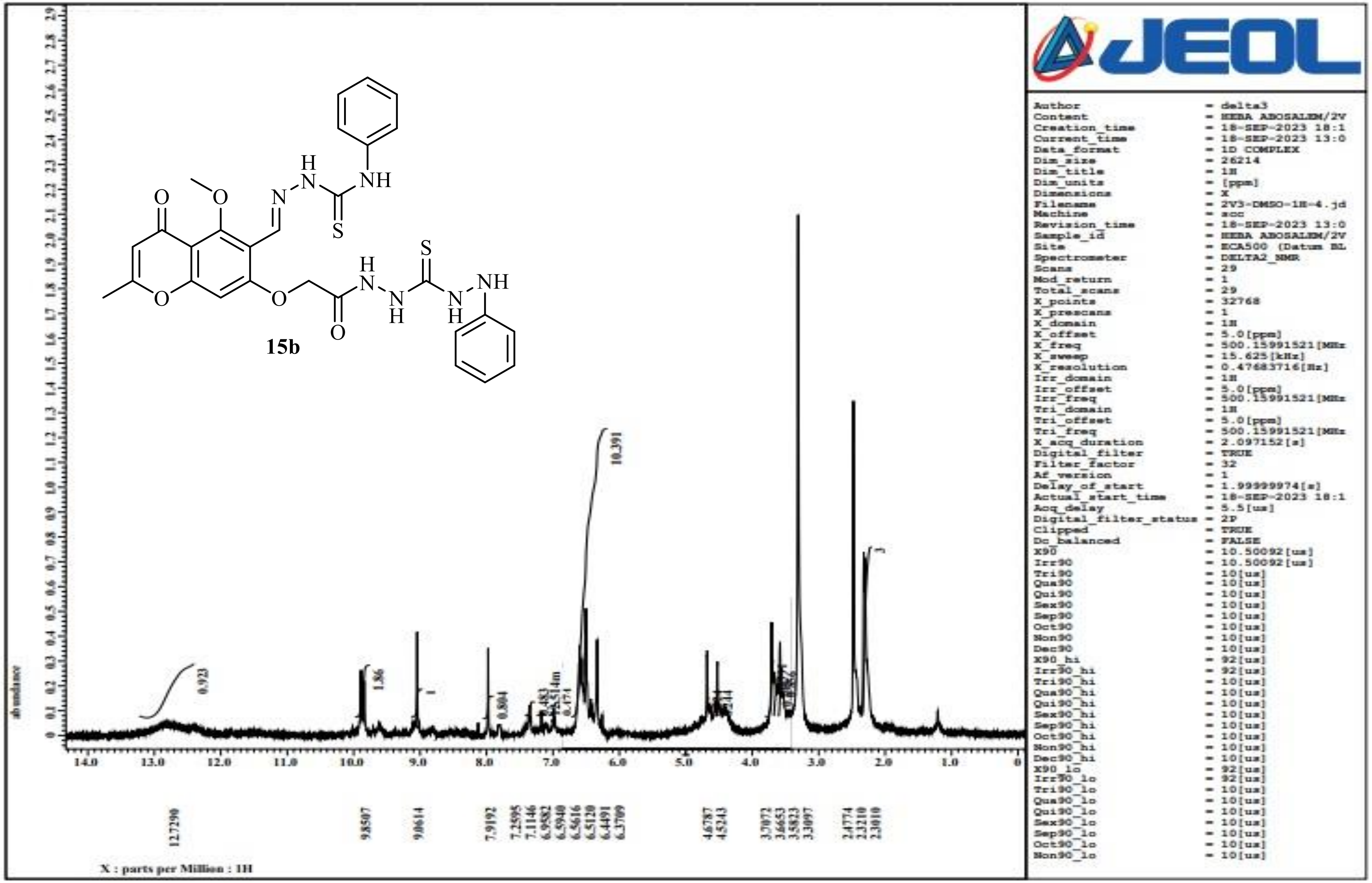

Figure s48: ^{13}CNMR (DMSO) spectrum for compound **15b**

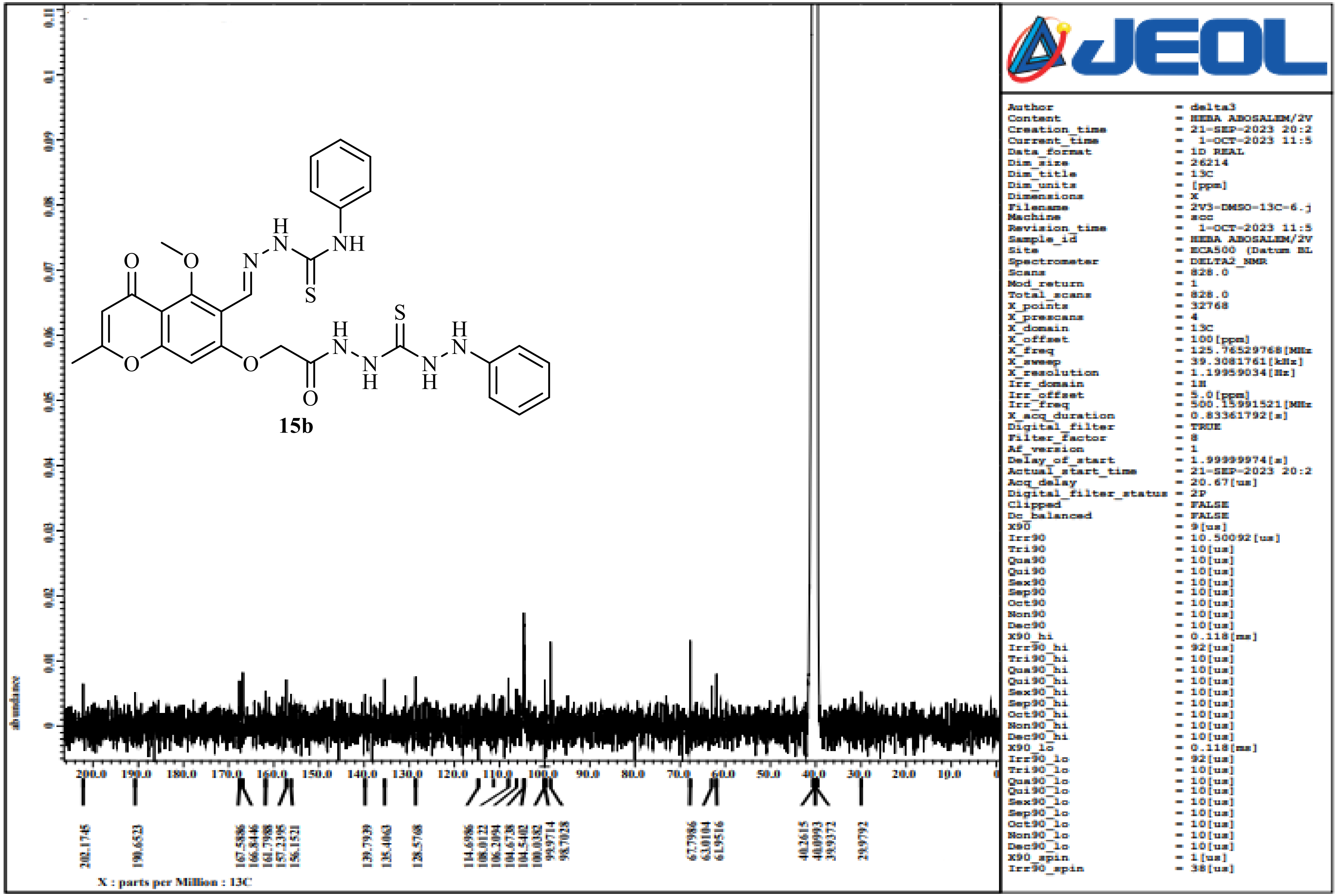

Figure s49: Mass spectrum for compound **15b**

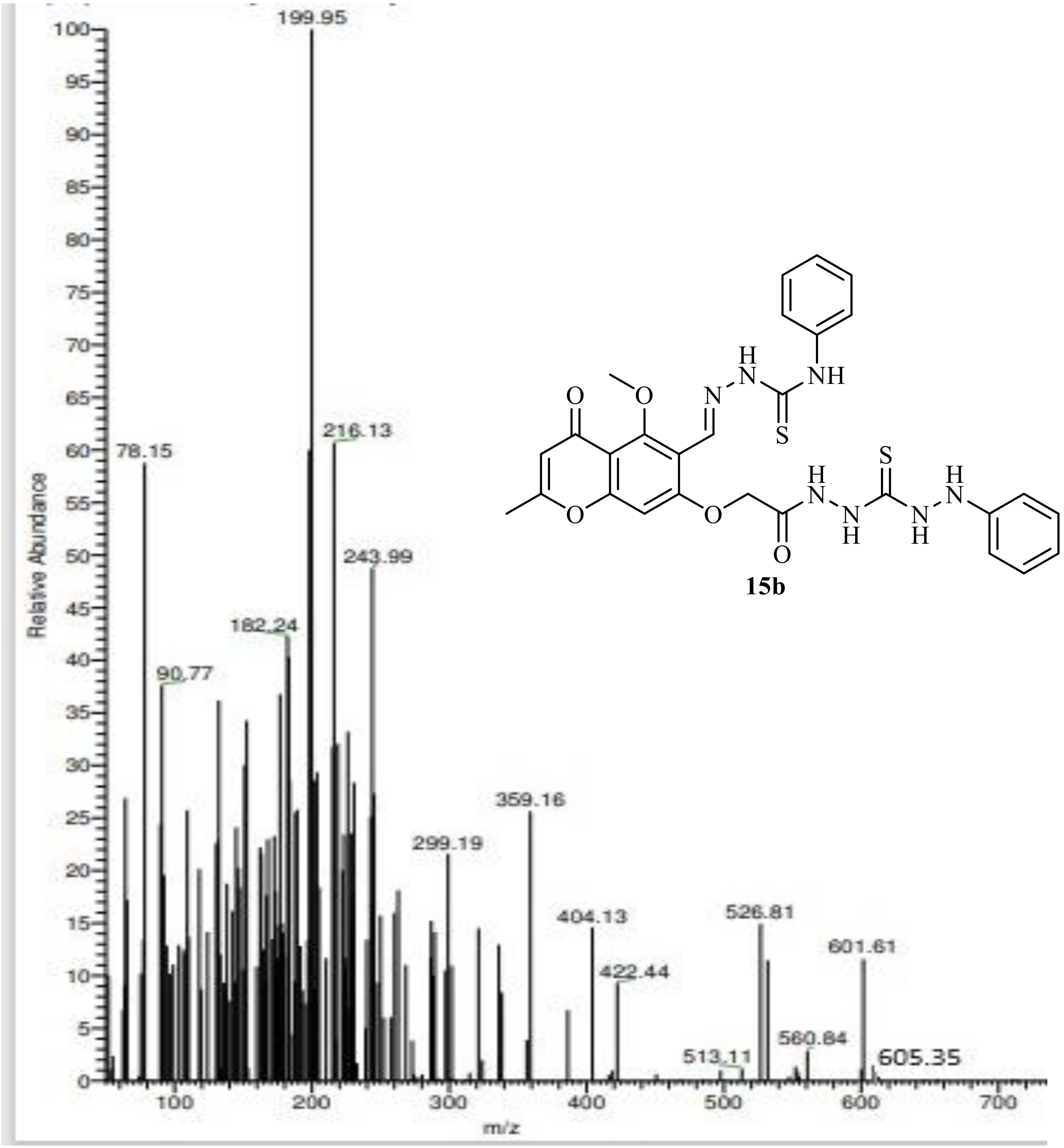

Figure s50: ^{1}HNMR (DMSO) spectrum for compound **15c**

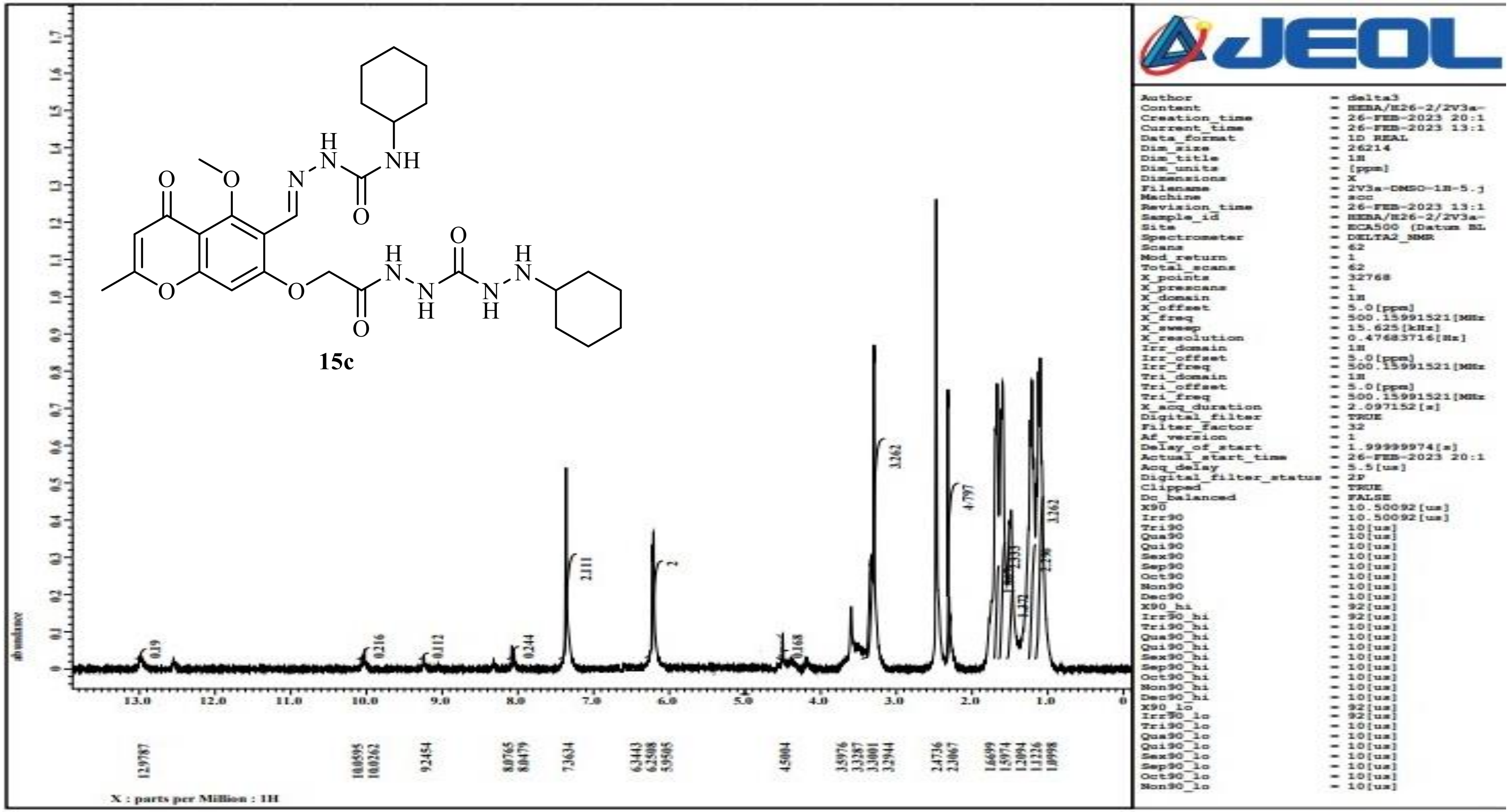

Figure s51: Mass spectrum for compound **15c**

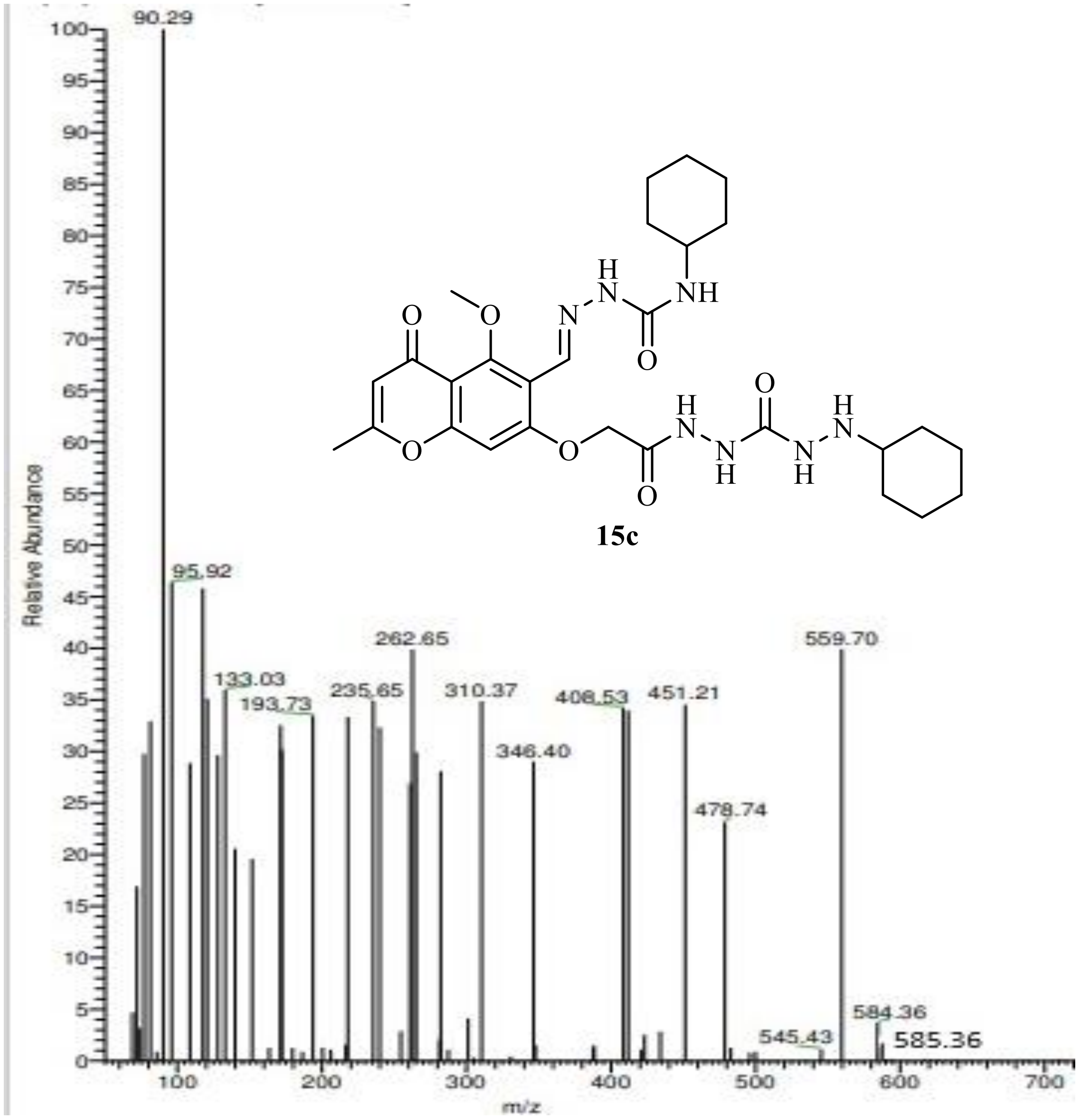

Figure s52: IR spectrum for compound **16**

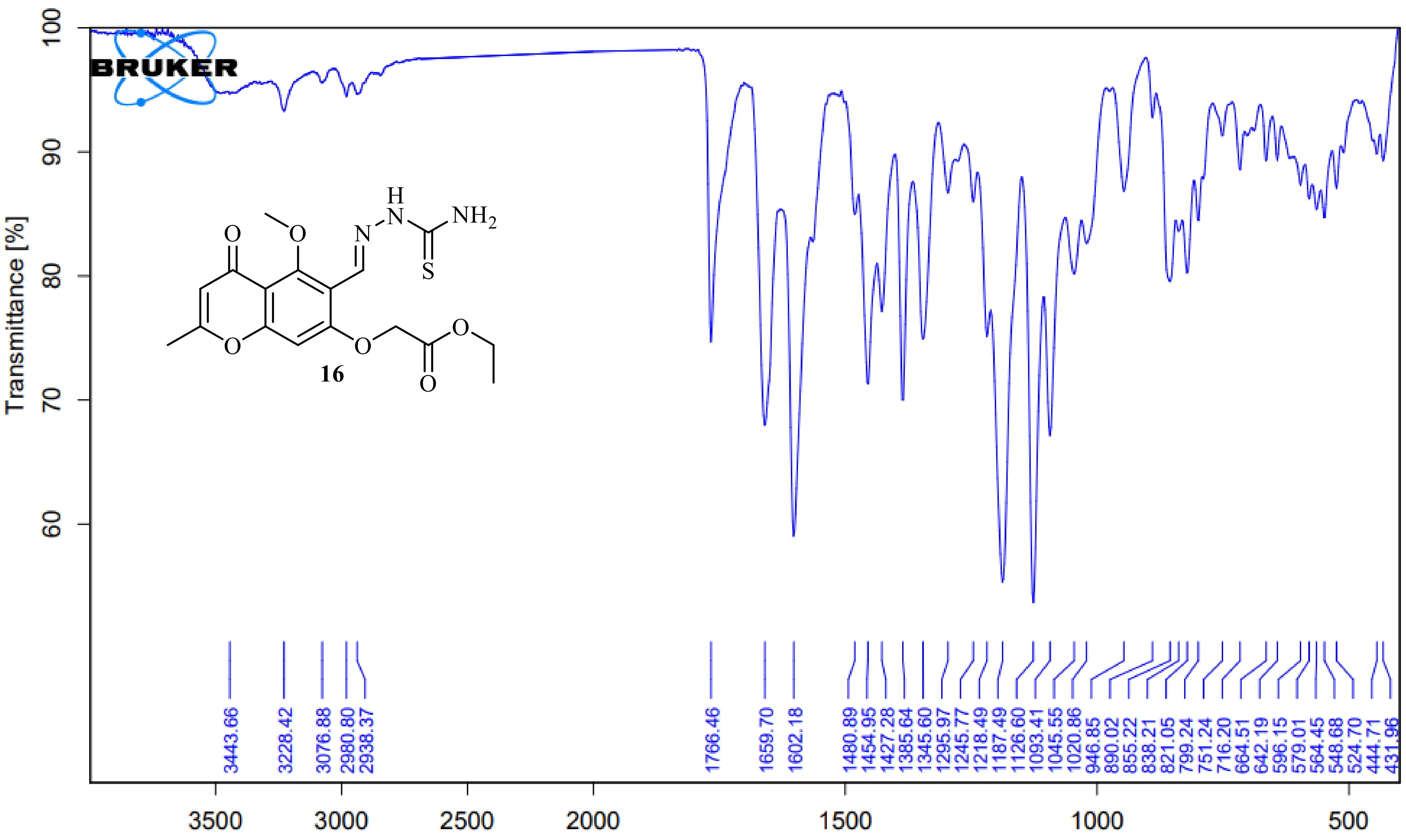

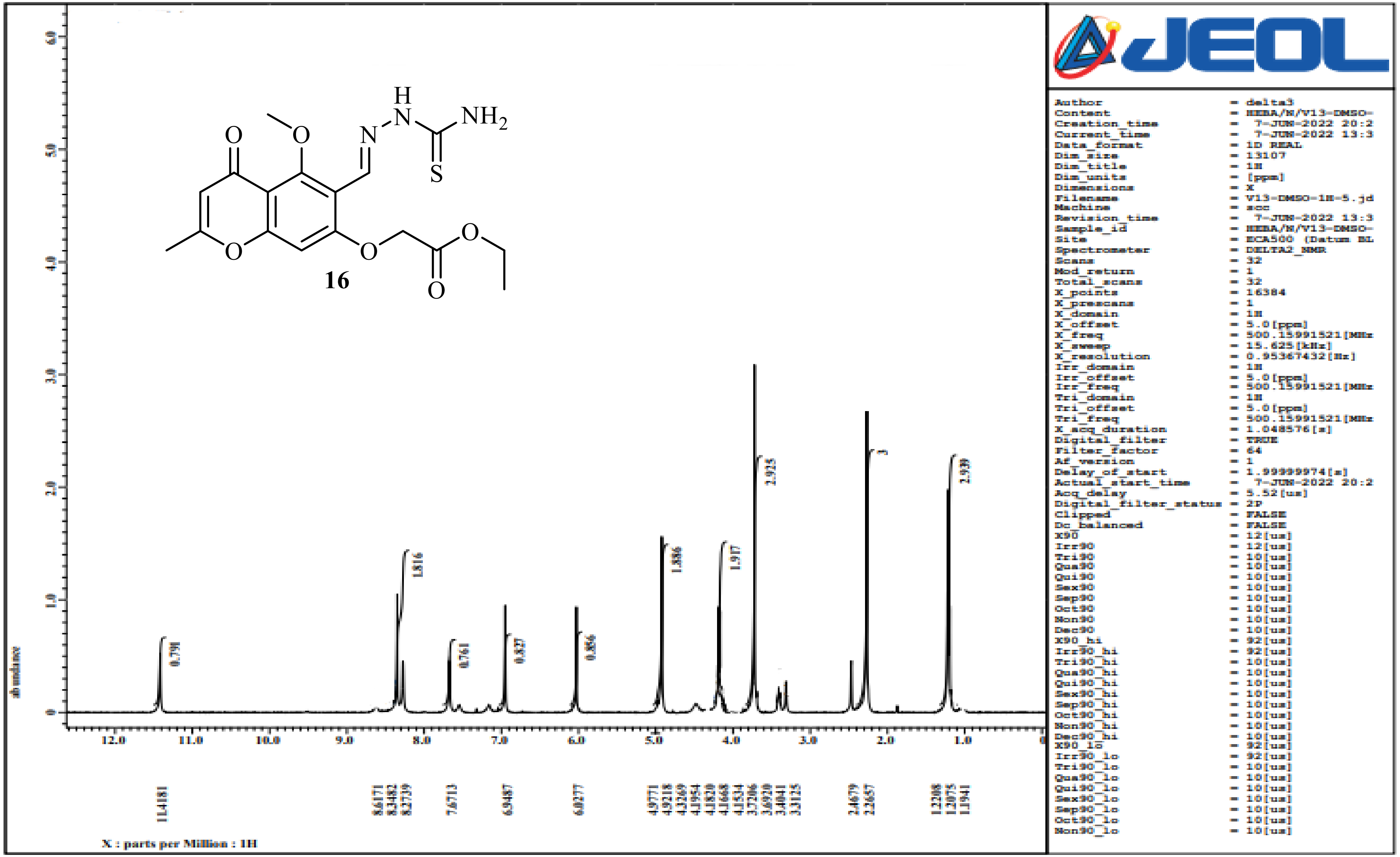

Figure s53: ^{1}HNMR (DMSO) spectrum for compound **16**

Figure s54: ^{13}CNMR (DMSO) spectrum for compound **16**

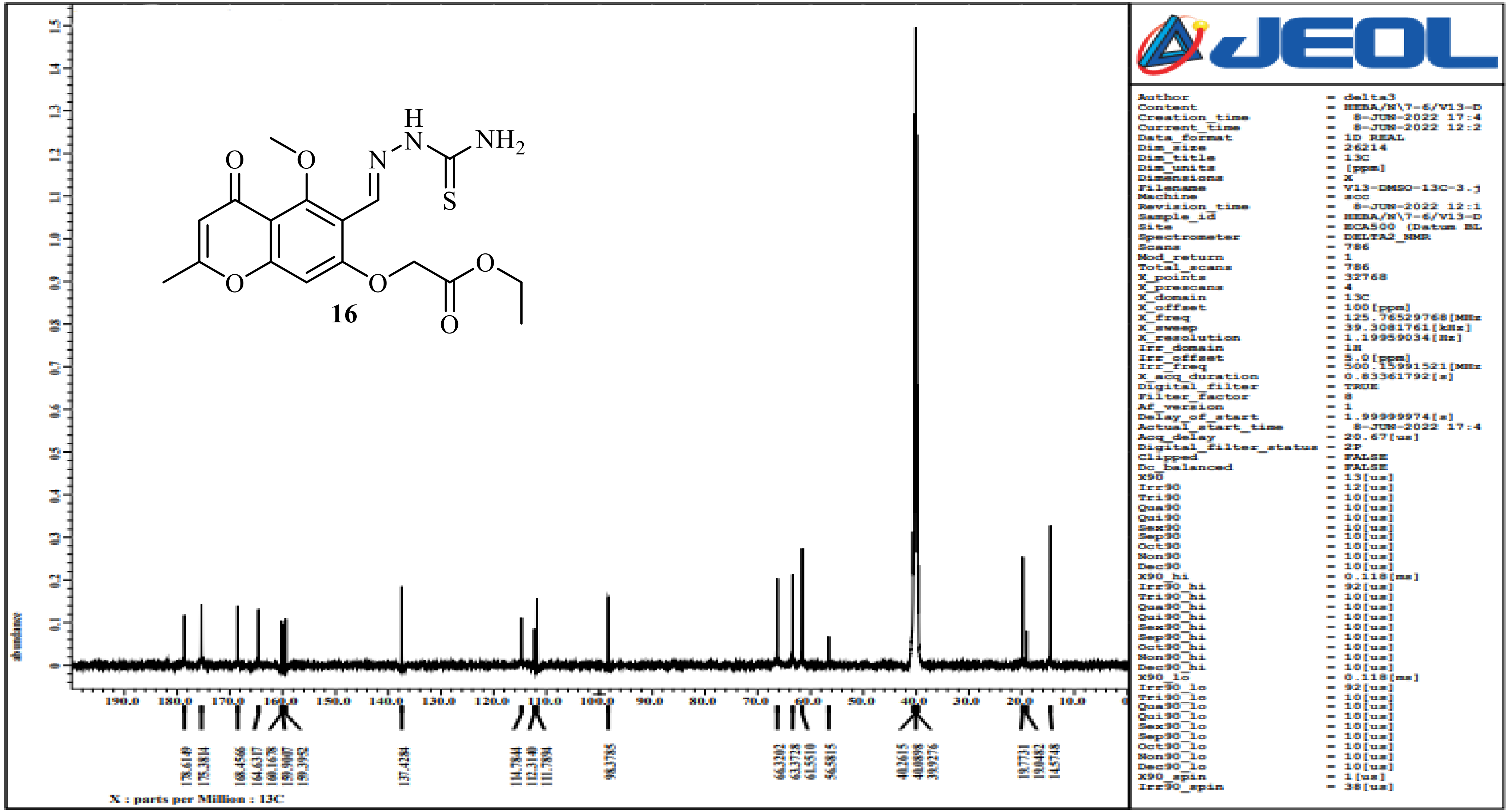

Figure s55: IR spectrum for compound **17**

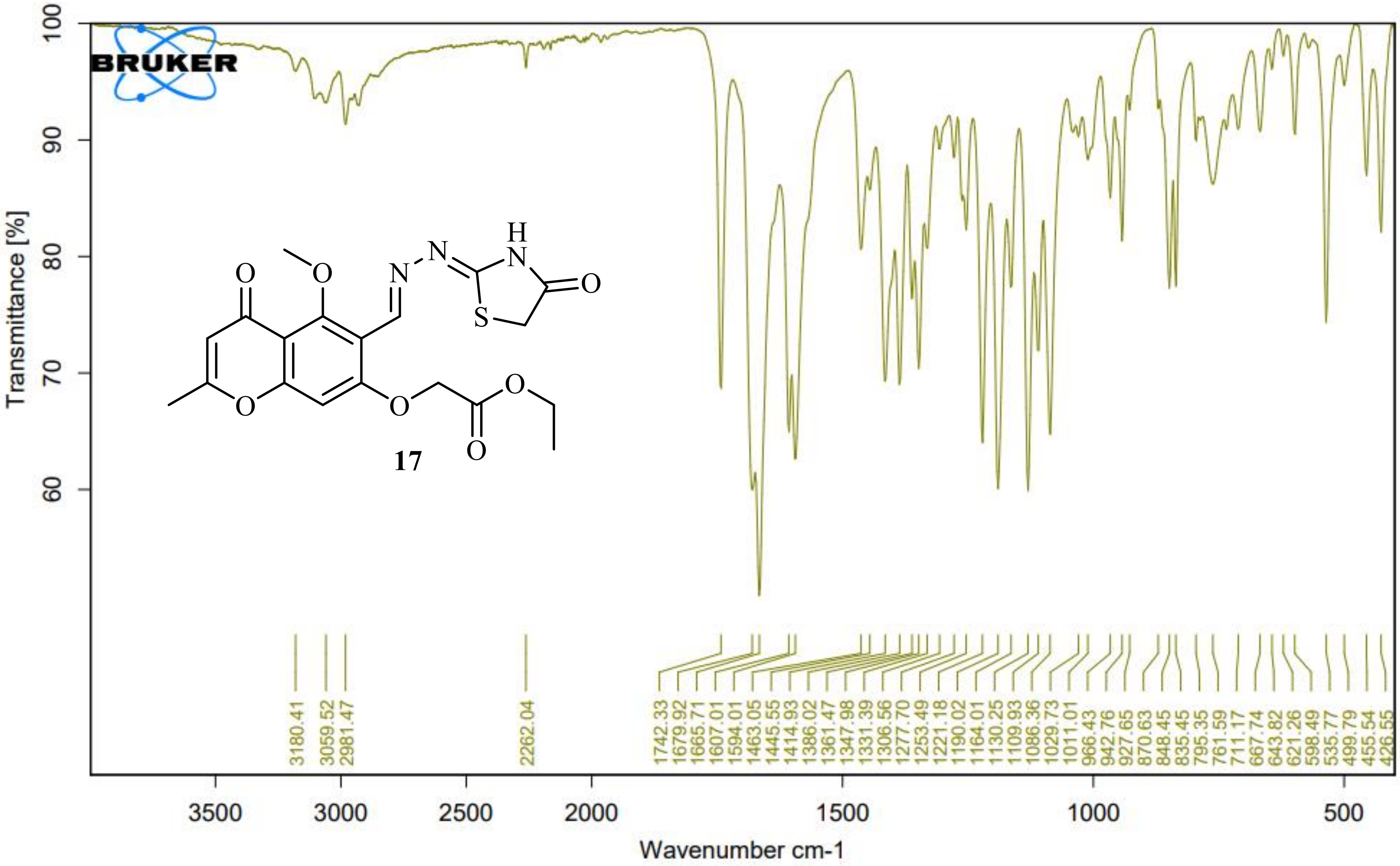

Figure s56: ^{1}HNMR (DMSO) spectrum for compound **17**

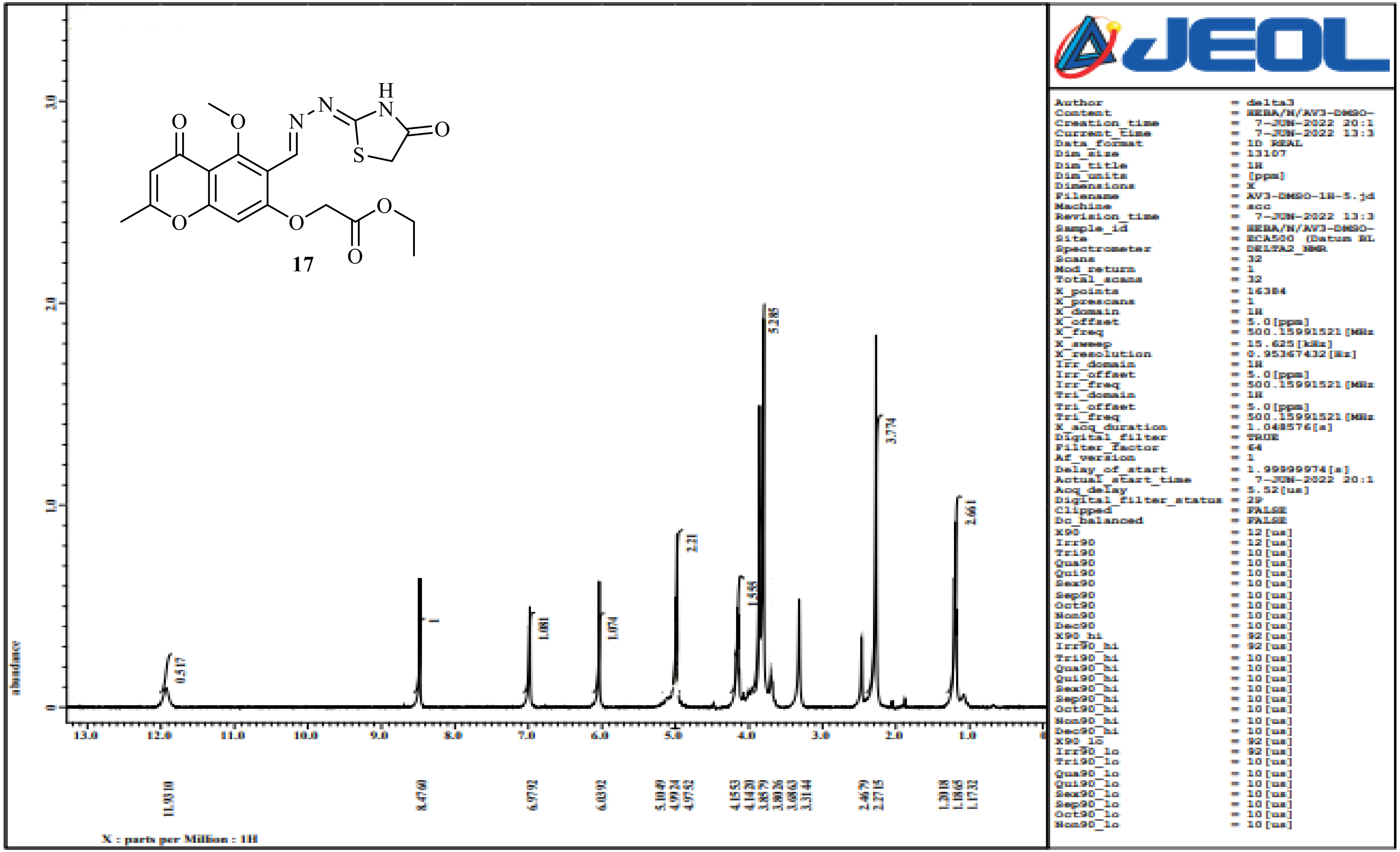

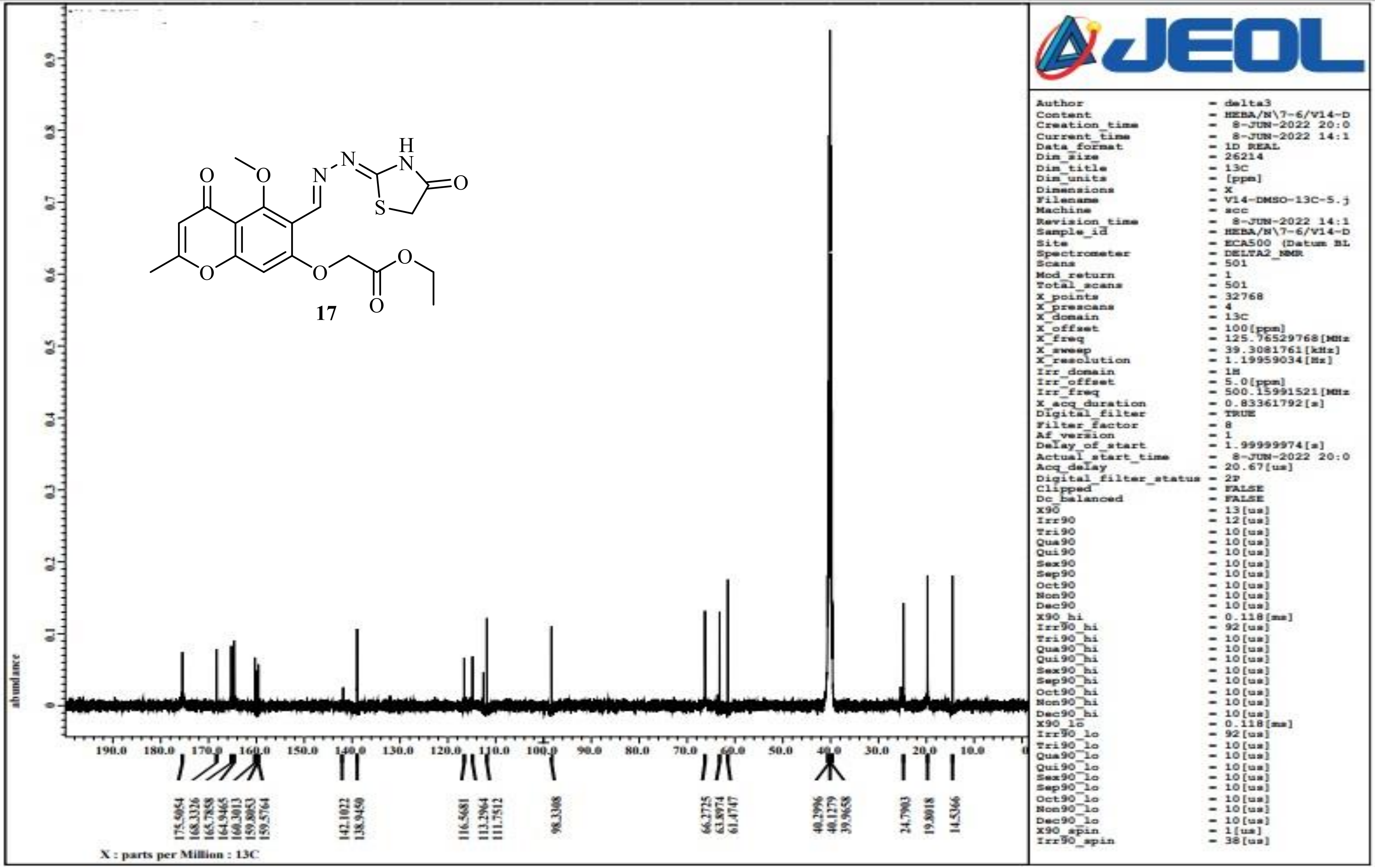

Figure s57: ^{13}CNMR (DMSO) spectrum for compound **17**

Figure s58: IR spectrum for compound **18**

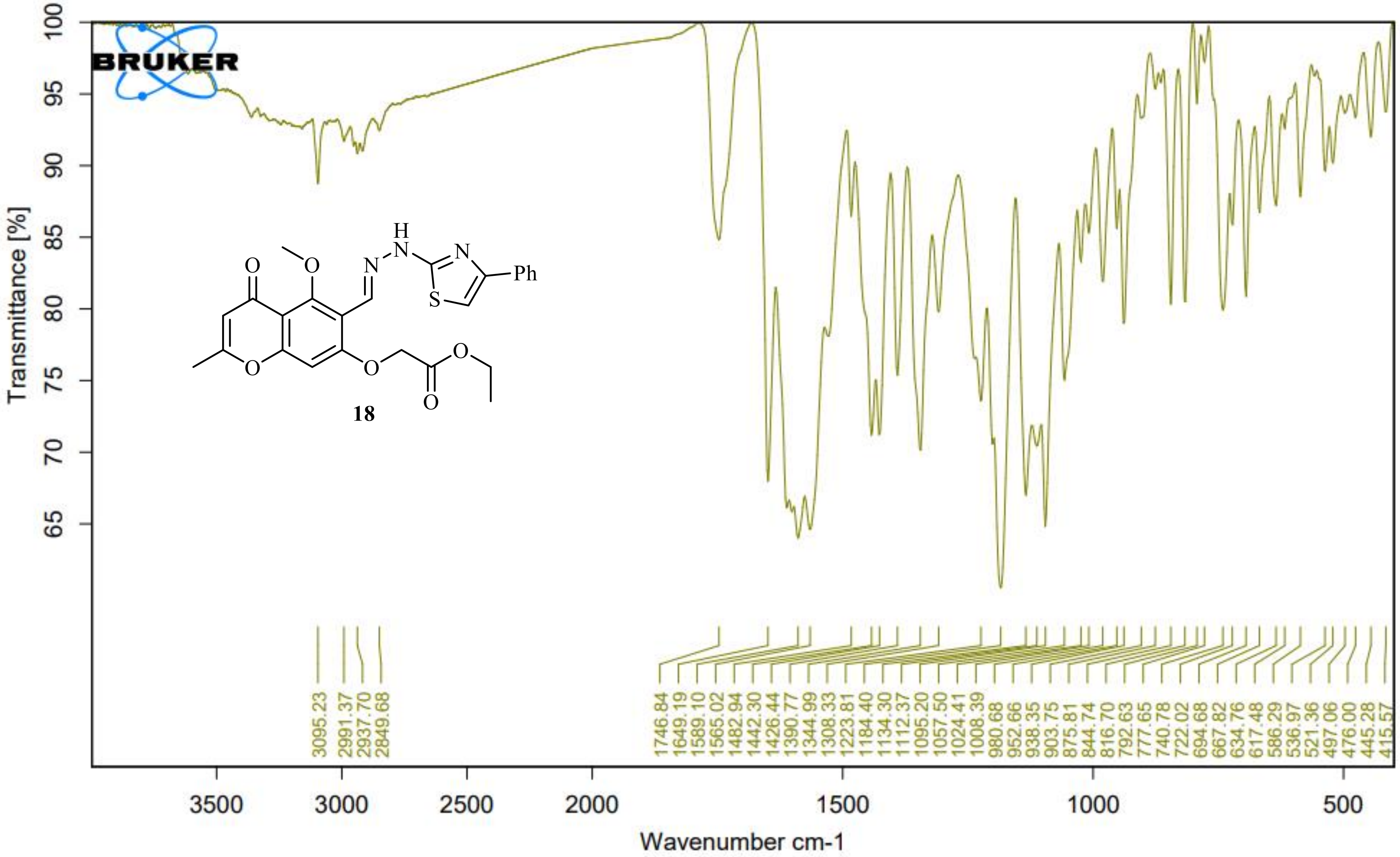

Figure s59: ^{1}HNMR (DMSO) spectrum for compound **18**

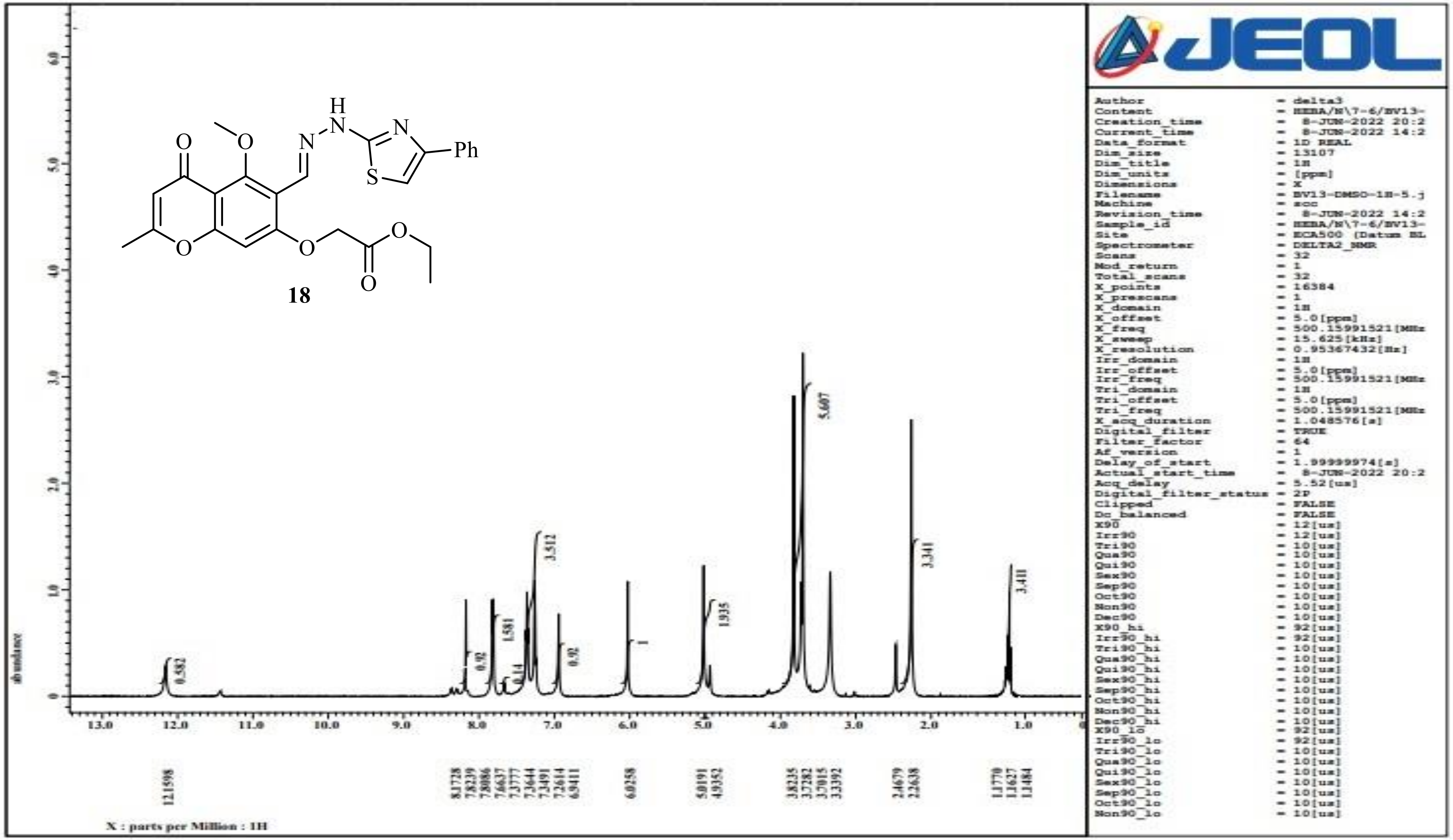

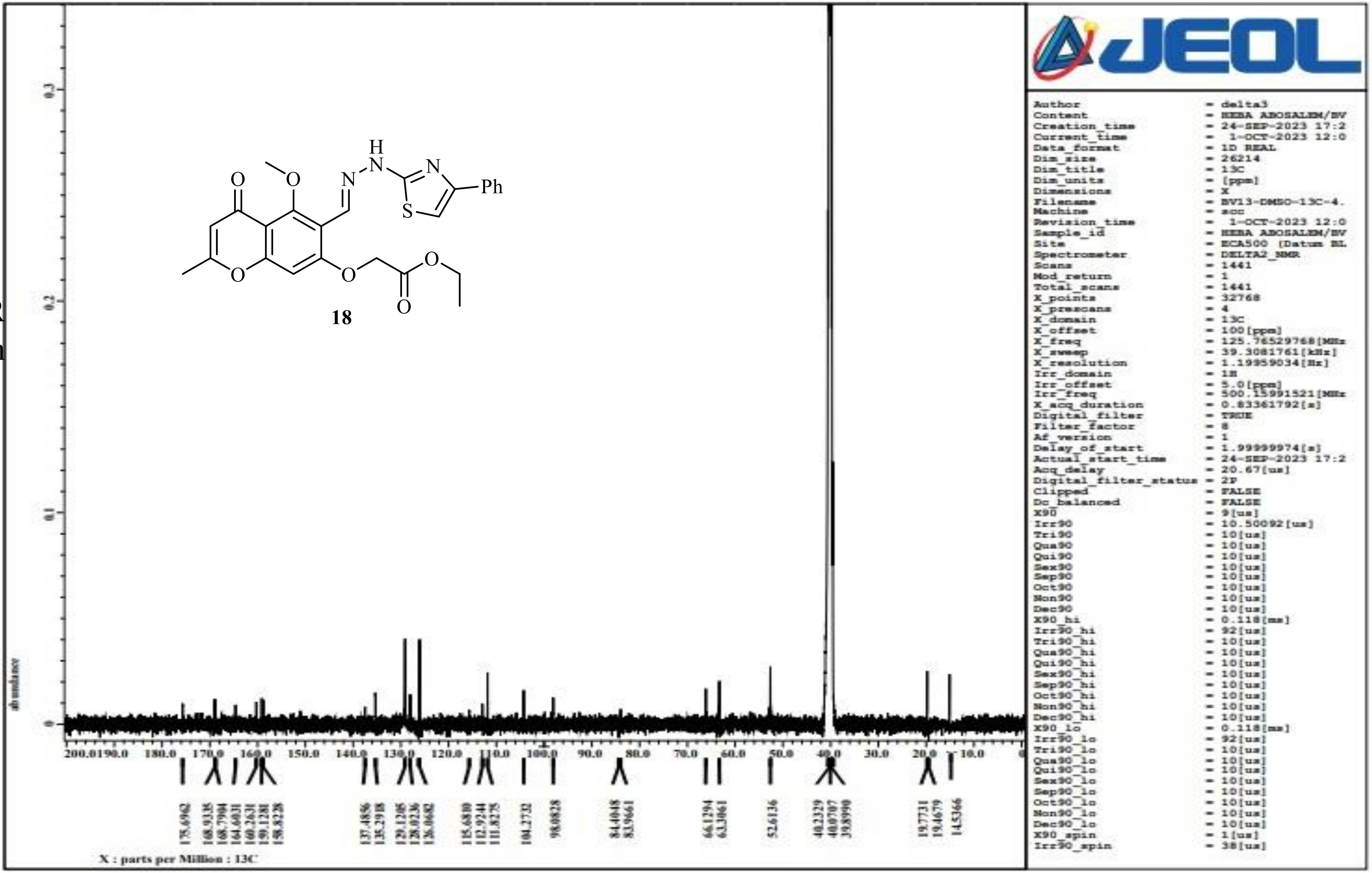

Figure s60: ^{13}CNMR (DMSO) spectrum for compound **18**

Figure s61: IR spectrum for compound **19**

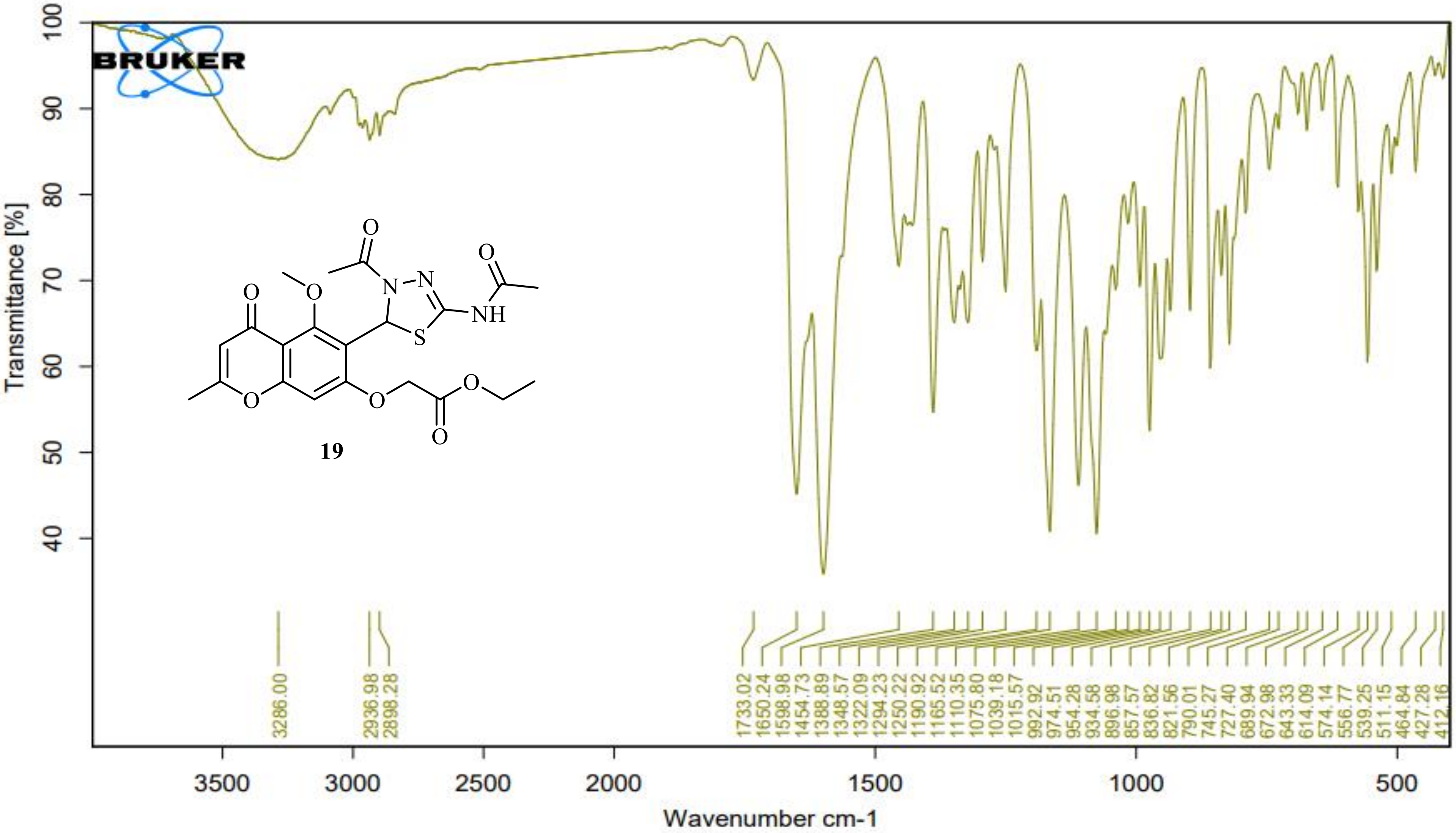

Figure s62: ^{1}HNMR (DMSO) spectrum for compound **19**

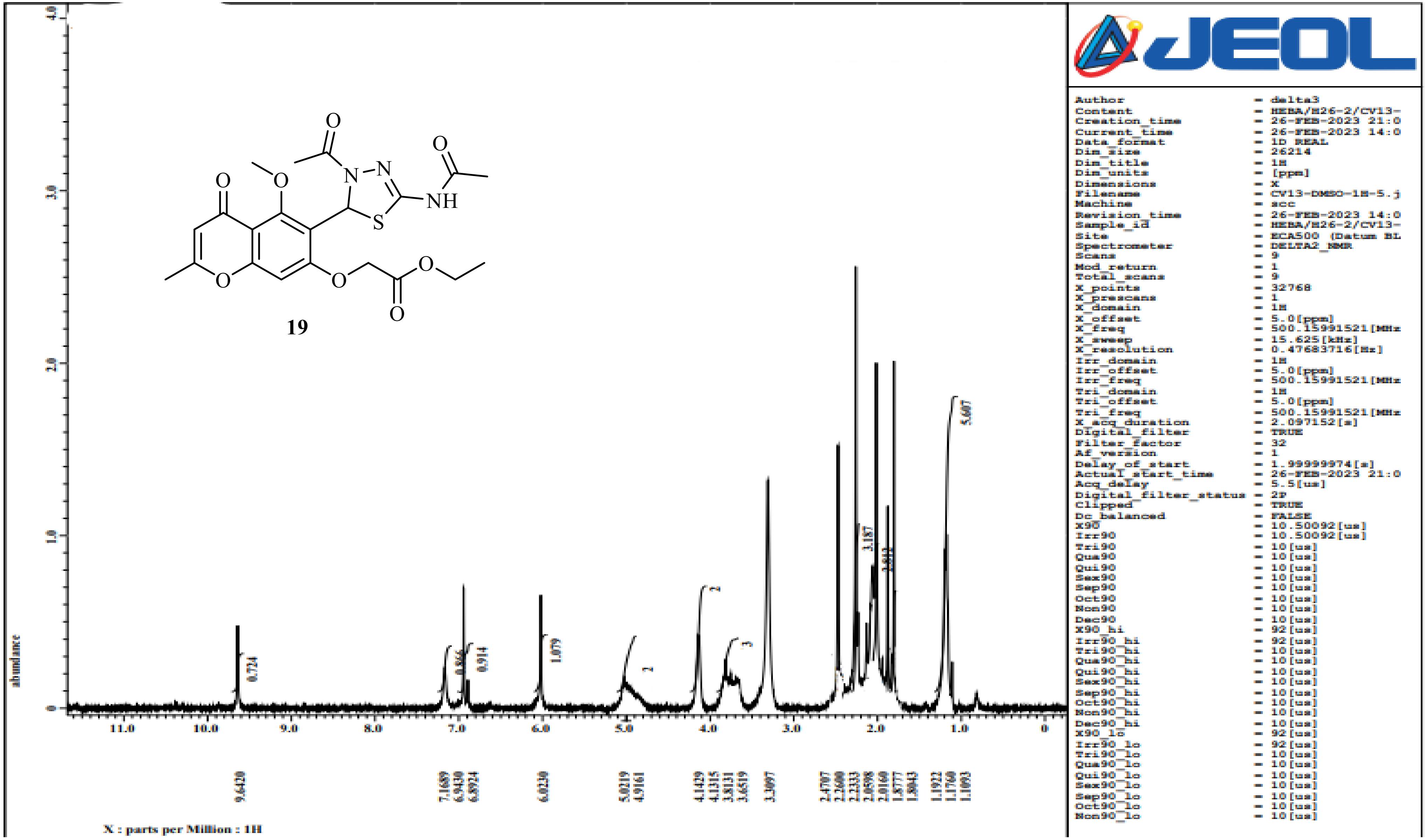

Figure s63: Mass spectrum for compound **19**

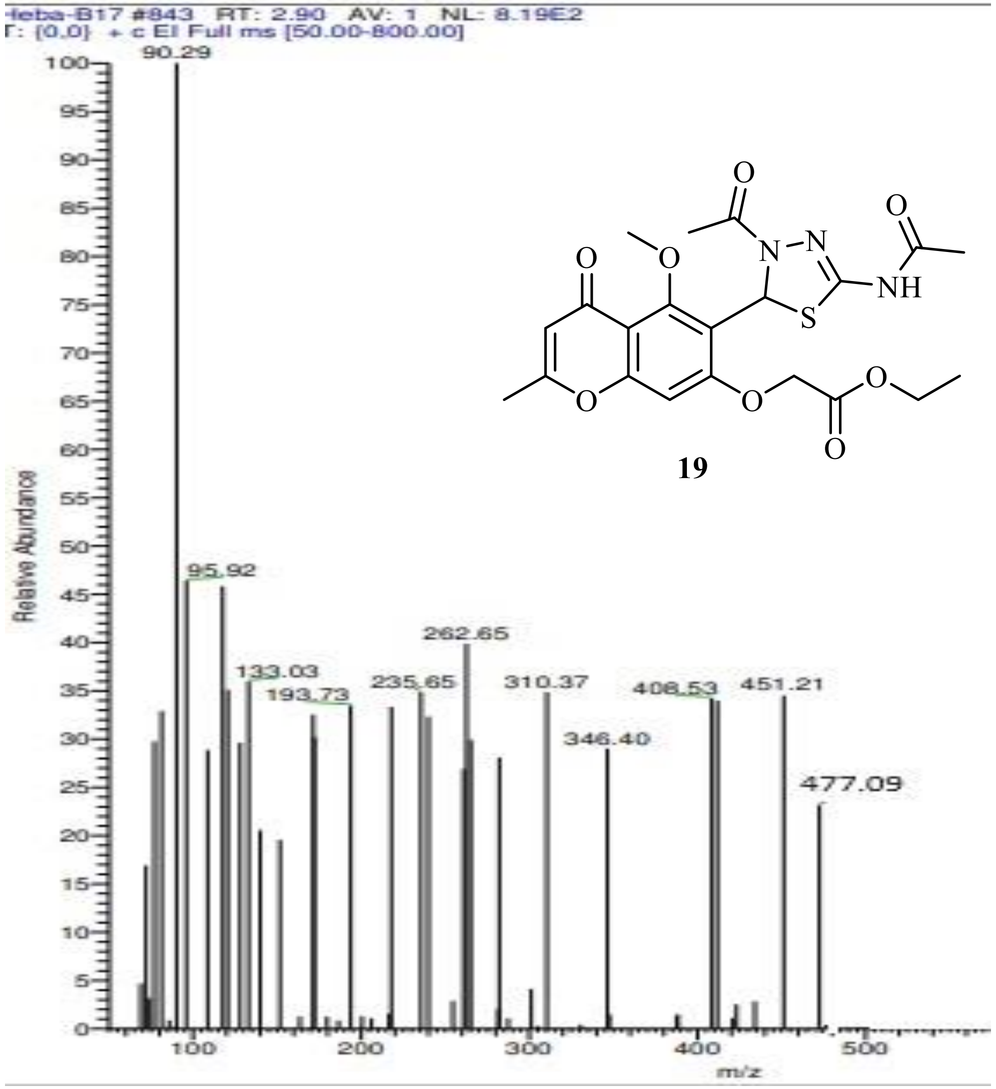

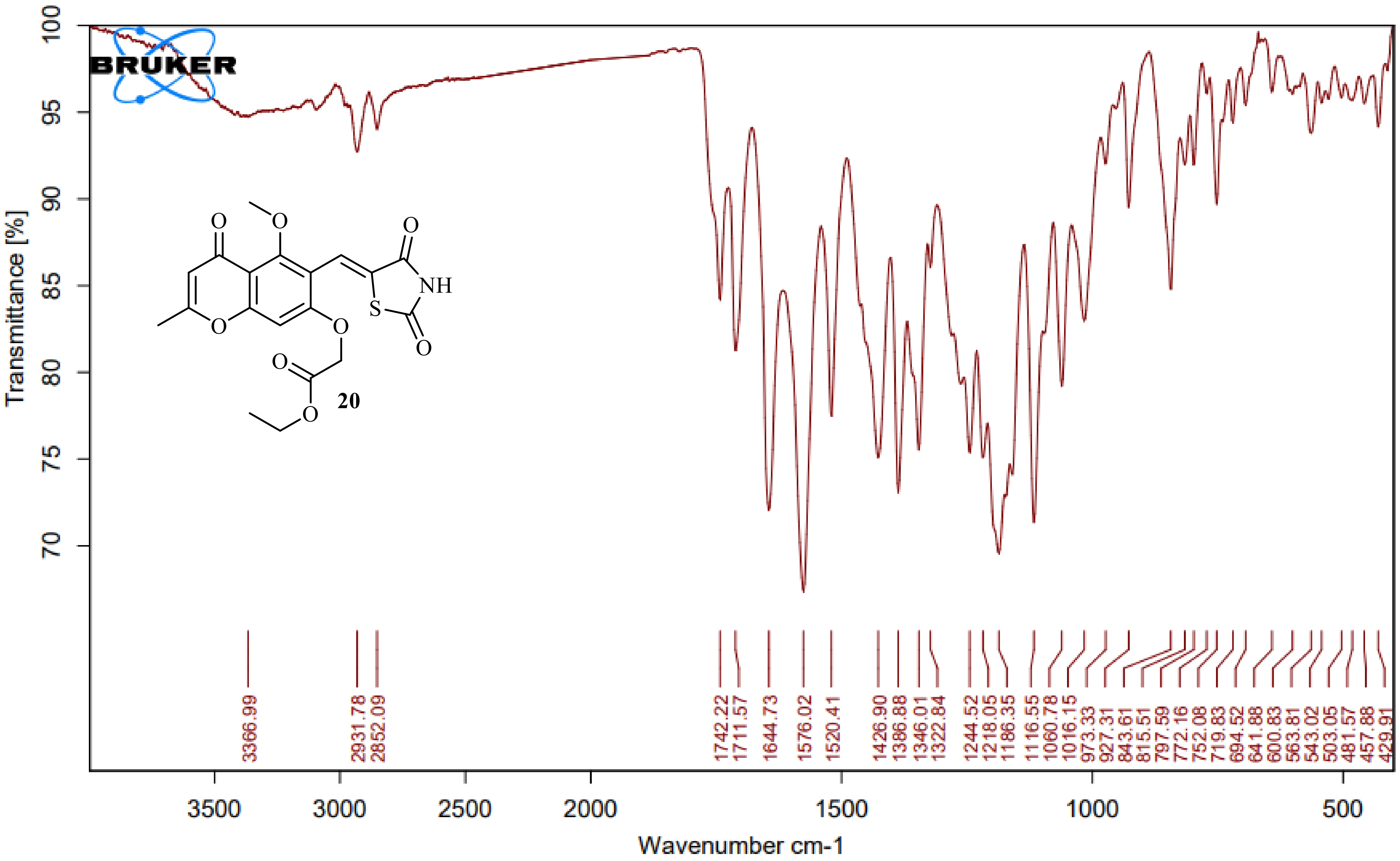

Figure s64: IR spectrum for compound **20**

Figure s65: ^{1}HNMR (DMSO) spectrum for compound **20**

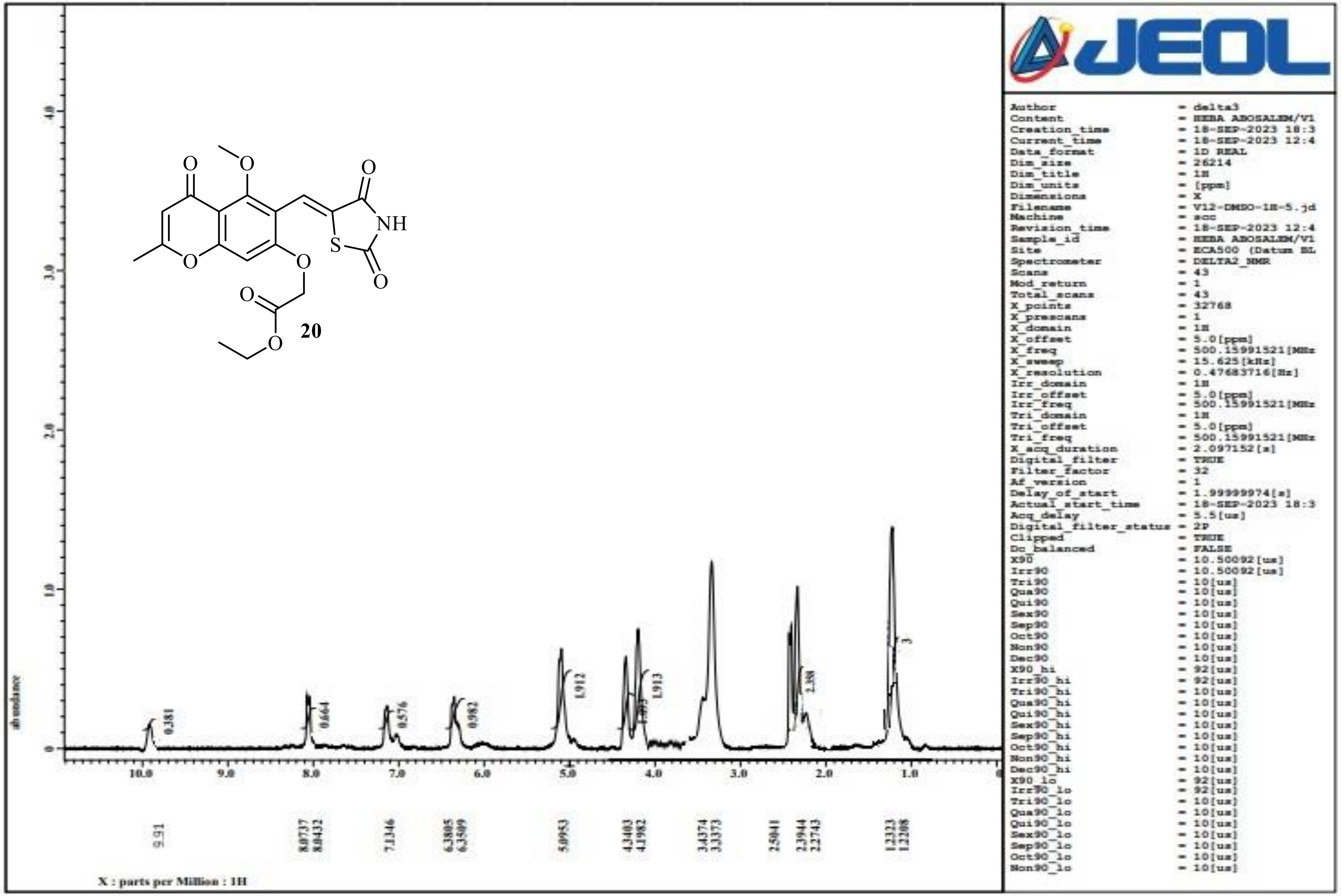

Figure s66: ^{13}CNMR (DMSO) spectrum for compound **20**

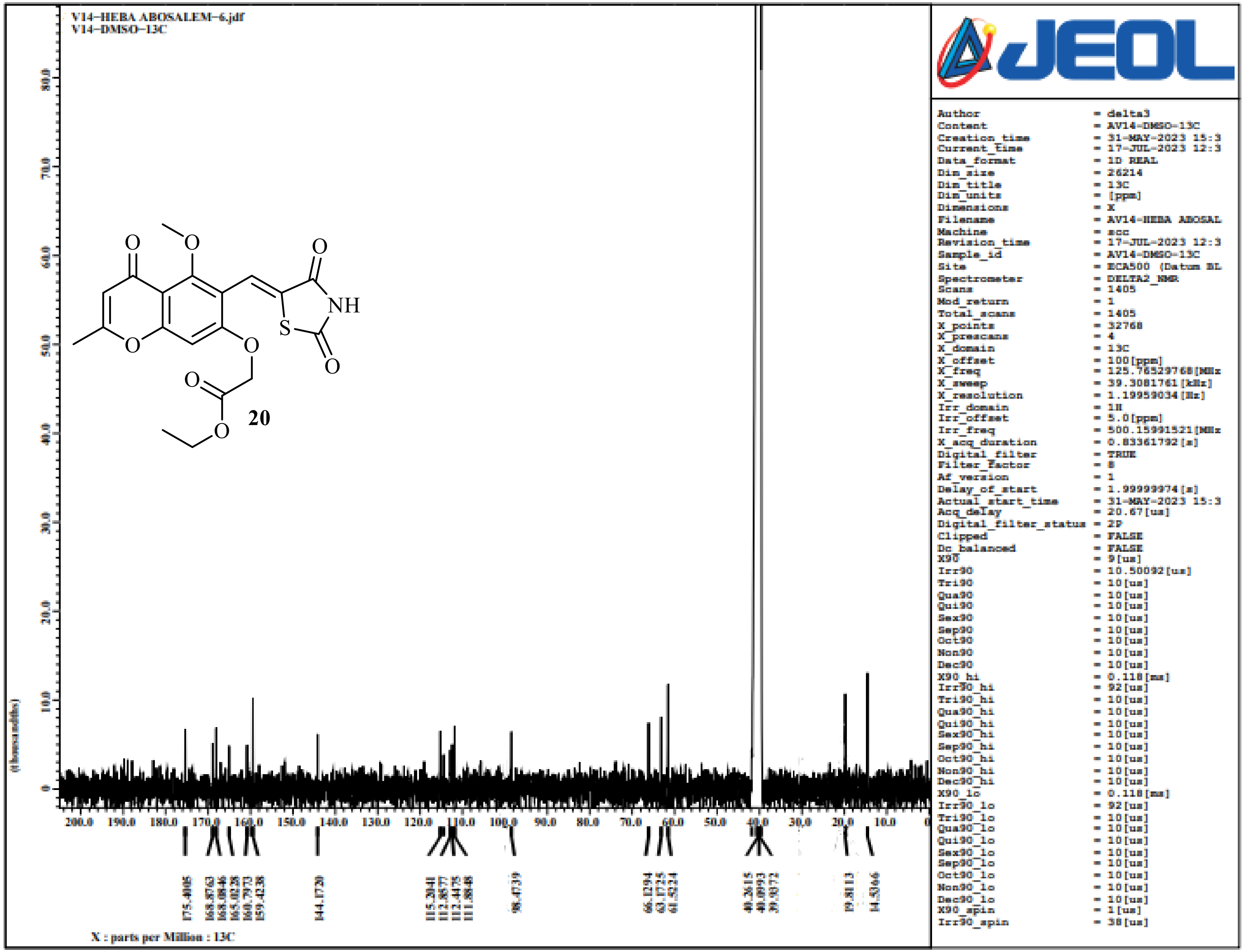

Figure s67: Mass spectrum for compound **20**

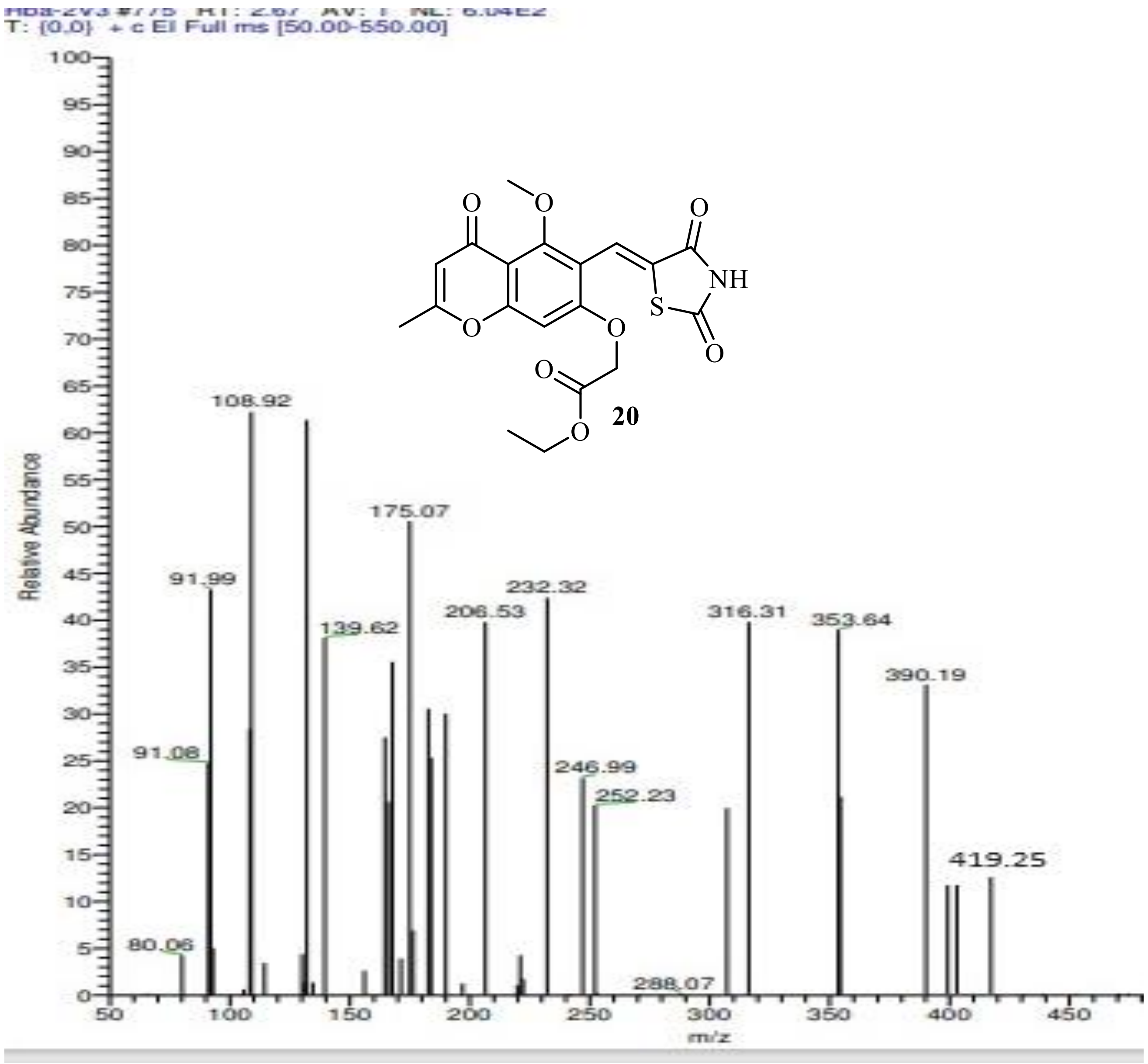

Supplement: Supplementary file 1 — Supplementary Figures. [file 41598_2024_59606_MOESM1_ESM.pdf]
